# 4-Dialkylamino-2,5-dihydroimidazol-1-oxyls with Functional Groups at the Position 2 and at the Exocyclic Nitrogen: The pH-Sensitive Spin Labels

**DOI:** 10.3390/gels8010011

**Published:** 2021-12-23

**Authors:** Dmitrii G. Trofimov, Yuri I. Glazachev, Artem A. Gorodetsky, Denis A. Komarov, Tatyana V. Rybalova, Igor A. Kirilyuk

**Affiliations:** 1 N. N. Vorozhtsov Novosibirsk Institute of Organic Chemistry SB RAS, 630090 Novosibirsk, Russia; ditrof@nioch.nsc.ru (D.G.T.); gorodaa@nioch.nsc.ru (A.A.G.); dkomarov@nioch.nsc.ru (D.A.K.); rybalova@nioch.nsc.ru (T.V.R.); 2 Voevodsky Institute of Chemical Kinetics and Combustion SB RAS, 630090 Novosibirsk, Russia; glaza@kinetics.nsc.ru

**Keywords:** nitroxide, spin label, spin probe, EPR, local pH, surface electrostatics, near-surface layer

## Abstract

Local acidity and electrostatic interactions are associated both with catalytic properties and the adsorption activity of various materials, and with the vital functions of biomolecules. The observation of acid–base equilibria in stable free radicals using EPR spectroscopy represents a convenient method for monitoring pH changes and the investigation of surface electrostatics, the advantages of which are especially evident in opaque and turbid samples and in porous materials such as xerogels. Imidazoline nitroxides are the most commonly used pH-sensitive spin probes and labels due to the high sensitivity of the parameters of the EPR spectra to pH changes, their small size, and their well-developed chemistry. In this work, several new derivatives of 4-(*N*,*N*-dialkylamino)-2,5-dihydrioimidazol-1-oxyl, with functional groups suitable for specific binding, were synthesized. The dependence of the parameters of their EPR spectra on pH was studied. Several showed a pK_a_ close to 7.4, following the pH changes in a normal physiological range, and some demonstrated a monotonous change of the hyperfine coupling constant by 0.14 mT upon pH variation by four units.

## 1. Introduction

Interfacial phenomena and local protonation effects play an important role in biophysics, biochemistry, and in the chemistry of heterogeneous systems [1]. Catalytic and sorption properties of various materials are dependent on the local acidity and electrostatic interactions inside the pores [2]. Measurements of the local acidity and electrostatic potential of the inner pore surfaces represent a problem of great practical interest. Several methods have been developed for the characterization of the acid–base properties of different surface locations [3].

EPR spectroscopy of ionizable nitroxides is a convenient method for the investigation of the above-mentioned phenomena [2,4,5], and is fully applicable to opaque or turbid materials [5,6]. Nitroxide spin probes are small enough to penetrate directly into the pores and to be adsorbed onto the surface of the material under study. The protonation of basic centers in specially designed spin probes affects the hyperfine coupling ***A***-tensor and ***g***-factor matrix, as well as the rotational dynamics of the nitroxide molecule in the proximity of charged surfaces, and this is reflected in the EPR spectra [7]. An analysis of these data gives information about the acidic centers in the material and the local surface electrostatic potential. Recently, EPR studies using pH-sensitive spin probes were successfully used for the investigation of binary TiO_2_-SiO_2_ xerogels [8].

**Scheme 1:**
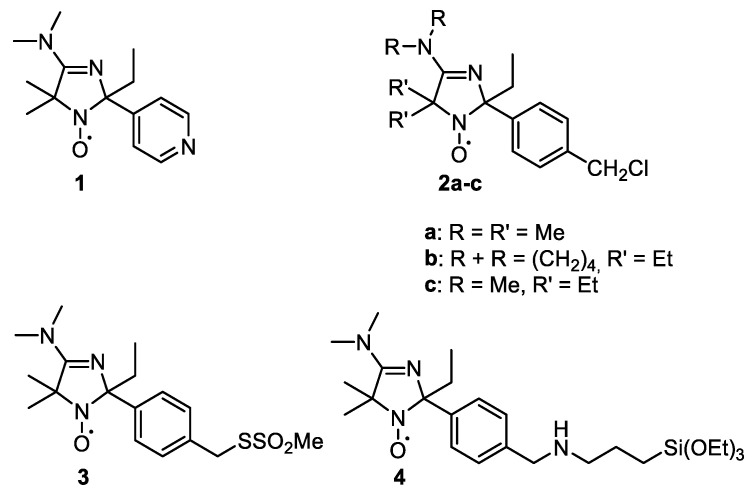
Structure of the nitroxides **1–4**.

Imidazoline nitroxides are the most commonly used pH-sensitive spin probes and labels due to the high sensitivity of the parameters of the EPR spectra to pH changes, their small size, and their well-developed chemistry. A large number of pH-sensitive nitroxides of the imidazoline series have been prepared [1,4,9]. Some of them are highly sensitive to pH changes in physiologically important regions. The development of a convenient method for the synthesis of 4-(*N*,*N*-dialkylamino)-2,5-dihydrioimidazol-1-oxyls from 4*H*-imidazole-3-oxides [10] allowed for easy variation of the substituents in position two of the heterocycle to prepare useful spin probes. Examples illustrating the benefits of this strategy include the synthesis of nitroxides with two pK_a_ values showing high sensitivity in a broad range of pH [11,12], e.g., Scheme 1, label **1**, and pH-sensitive alkylating spin, labels **2a–c** [13,14,15], which were used to prepare the hydrophilic spin probes from glutathione [13,14], thiol-specific pH-sensitive spin, label **3**, for site-directed labeling of proteins and lipids [16,17], and siloxane-derived spin, label **4**, capable of binding to silica or alumina surfaces [7]. Despite the significant advances in this area, the broad variety of potential research objects produces a request for new pH-sensitive spin labels capable of specific attachment. Here we describe a new set of pH-sensitive imidazoline nitroxides with various functional groups in the side chain. Some of them may find an application in material science or in biophysics.

## 2. Results and Discussion

**Scheme 2:**
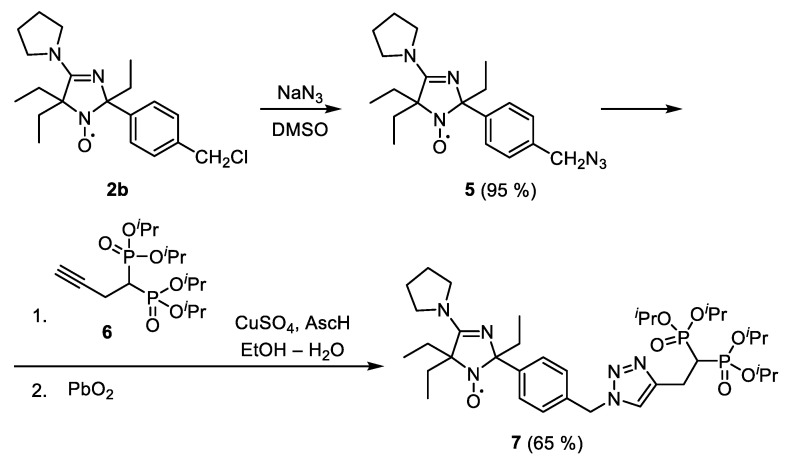
Synthesis of the nitroxide **7**.

The high reactivity of **2** in nucleophilic substitution reactions offers easy access to new functional derivatives. Expectedly, **2b** readily reacts with sodium azide to produce **5** with a nearly quantitative yield. Nitroxide azides can be used for the spin labeling of acetylene-modified molecules via Huisgen 1,3-dipolar cycloaddition [18,19]. In analogy to the literature [20], a reaction of **5** with tetraisopropyl but-3-yne-1,1-diyldiphosphonate **6** in the presence of Cu(II) salt and ascorbic acid after subsequent re-oxidation produced **7** (Scheme 2).

**Scheme 3:**
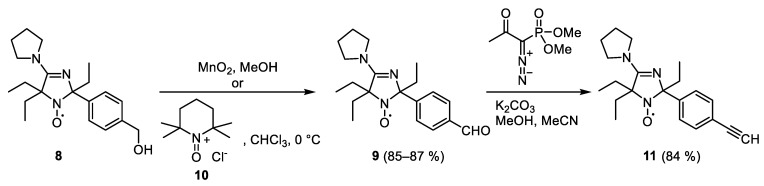
Synthesis of the nitroxide **11**.

The addition of nitroxides with a terminal acetylene group to azide-modified biomolecules, e.g., nucleic acids, is another way to use the Huisgen-click reaction for spin labeling. Terminal acetylenes can also be attached via Pd-catalyzed coupling [21]. A spin label with a terminal acetylene group was prepared from **8** in two steps (Scheme 3). The oxidation of benzyl alcohol **8** with the activated manganese dioxide in methanol smoothly led to the formation of the corresponding aldehyde **9**, with the nitroxyl group and the amidine moiety being unaffected. Alternatively, **8** can be oxidized to the aldehyde **9** with 1-oxo-2,2,6,6-tetramethylpiperidinium chloride **10** with similar yield. The aldehyde **9** readily reacts with Bestmann-Ohira reagent to produce **11** with a yield of 84% [22]. The structure of **11** was confirmed with X-ray analysis data (Figure S1).

**Scheme 4:**
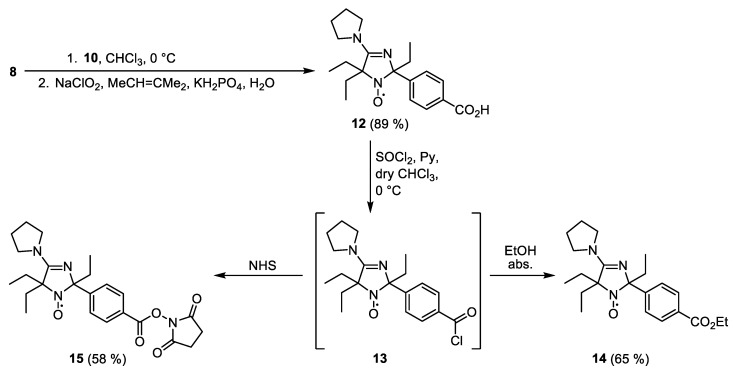
Synthesis and esterification of **12**.

Unless the attachment of a pH-sensitive nitroxide to a primary amino group is successfully performed via alkylation with **2b** [7], the acylation reaction is more selective, allowing for the binding of a single nitroxide to the site. Carboxylic acids can be easily prepared from **8**. Here we used a convenient one-pot process, where the reaction of **8** with an oxoammonium salt **10** was followed by the Lindgren-Kraus-Pinnick procedure [23] (Scheme 4). The carboxylic acid **12** was isolated with a 95% yield. To activate carboxylic group for acylation, the nitroxide **12** was treated with SOCl_2_ in the presence of pyridine. The chloroanhydride formed readily reacted with ethanol to give ester **14**. The reaction of **13** with *N*-hydroxysuccinimide (NHS) produced spin label **15**.

**Scheme 5:**
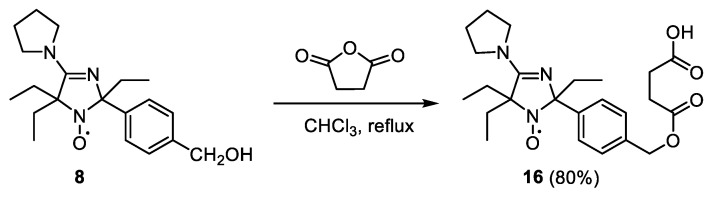
A reaction of **8** with succinic anhydride.

Another nitroxide with a carboxylic group on a longer spacer was prepared in one step from **8** via acylation with succinic anhydride (Scheme 5).

The EPR spectra of nitroxides **7**, **11**, **12**, **15**, and **16** are strongly pH-dependent with Δ*a*_N_ > 0.1 mT, and a pK_a_ between 6 and 6.7 (Table 1). The titration curves demonstrate optimal sensitivity in slightly acidic media [14,15], but the sensitivity is not optimal in the normal physiological range of 7.35–7.45 [24]. Similar structures without aromatic substituents are known to show higher pK_a_ values [11].

**Scheme 6:**
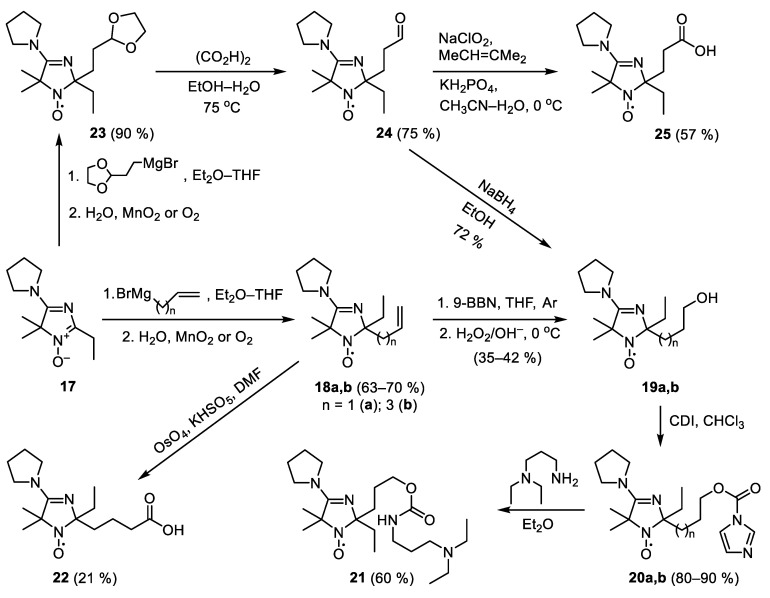
Synthesis of spin labels and spin probes from **17**.

To prepare 2-functionalized nitroxides with a pK_a_ above 7, the reaction of 4*H*-imidazol-3-oxide **17** with Grignard reagents was used (Scheme 6). The treatment of **17** with alkenylmagnesium bromides produced nitroxides with a terminal ethylene bond **18a,b**. The hydroboration of **18a,b** with 9-BBN, followed by oxidation with hydrogen peroxide, was performed using the protocol developed by Hideg for 2-allyl pyrrolidine nitroxides [25]. The reaction produced alcohols **19a,b**, which were then treated with carbonyldiimidazole (CDI) to give **20a,b**. To demonstrate the feasibility of the carbonylimidazole pH-sensitive spin labels for binding to primary amino groups, **20b** was allowed to react with *N*,*N*-diethyl-1,3-diaminopropane.

The nitroxide **21** was isolated with a 60% yield. The oxidative cleavage of the terminal double carbon–carbon bond in **18b** with osmium tetraoxide–oxone system yielded carboxylic acid **22**.

**Figure 1:**
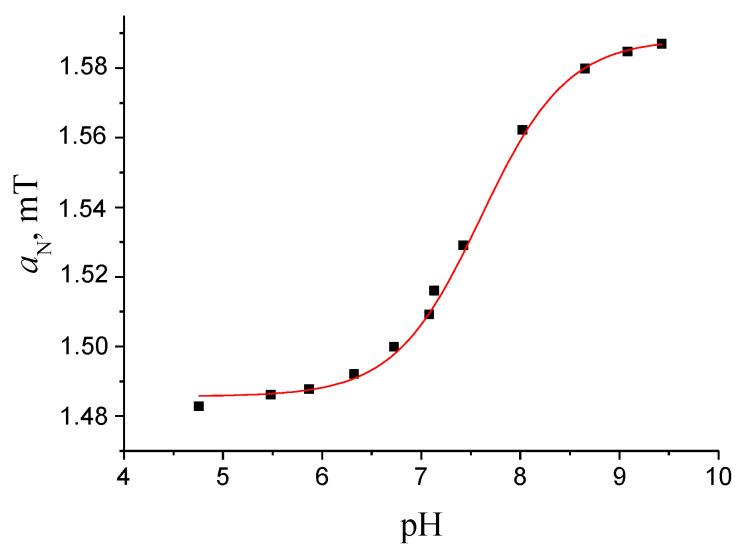
Titration curve of nitroxide **25**.

The addition of 2-(1,3-dioxolan-2-yl) ethylmagnesium bromide to **17** is another convenient way to create 2-functionalized pH-sensitive spin labels. The reaction produced nitroxide **23** with a high yield. The dioxolane protection group in **23** was readily removed under relatively mild conditions to give the corresponding aldehyde **24**, which can be either oxidized to carboxylic acid **25** using the Lindgren-Kraus-Pinnick procedure, or reduced with sodium borohydride to **19a**. The sequence **17** → **23** → **24** → **19a** gives a remarkably higher yield of the target nitroxide than the addition of allylmagnesium bromide with subsequent hydroboration. A titration of the nitroxides **20**, **22**, and **25** showed that they may be valuable spin labels and probes with high sensitivities to changes of pH within the physiological range (see Table 1, Figure 1 and Supplementary Materials).

An investigation of the surfaces of many inorganic and organo-inorganic materials (catalysts, sorbents, etc.) requires nitroxides with a high sensitivity to acidity changes within a broad range of pH. A good example of such a spin probe is two-pK_a_ nitroxide **1**, which was successfully used in numerous studies [8,26,27,28,29,30,31]. A covalent attachment of similar nitroxides to the surface of a catalyst or a sorbent may provide a useful method for studies of the near-surface layer in these materials. Here we designed analogs of **1** with a functional group in a substituent at the exocyclic nitrogen atom of the amidine moiety.

**Scheme 7:**
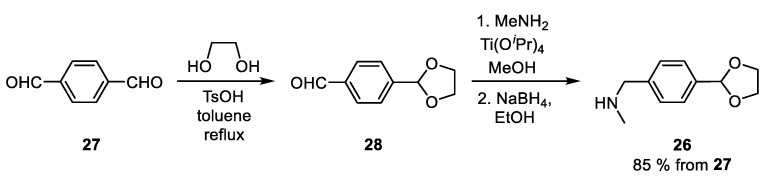
Synthesis of *N*-(4-(1,3-dioxolan-2-yl)benzyl)-*N*-methylamine (**26**).

*N*-(4-(1,3-dioxolan-2-yl)benzyl)-*N*-methylamine **26** was prepared in two steps from tereftaldicarboxaldehyde **27** (Scheme 7).

**Scheme 8:**
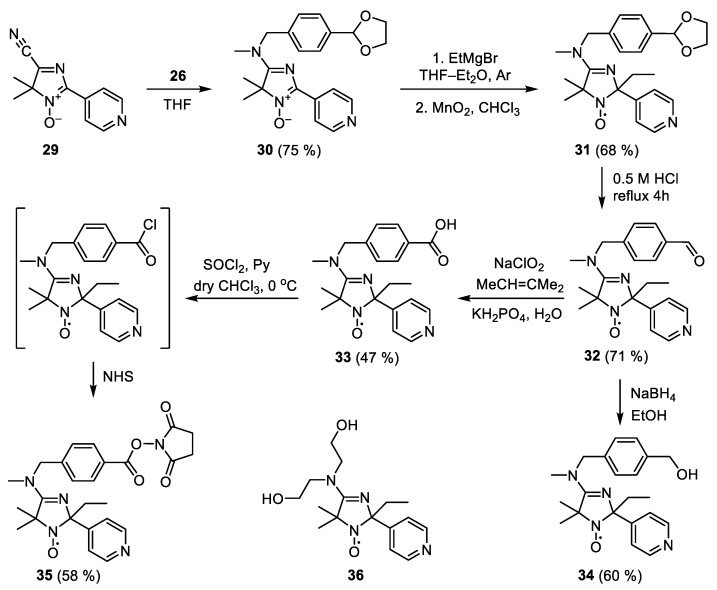
Synthesis of two-pK_a_ nitroxides. Structure of the nitroxide **36**.

A reaction of the 5-cyano-4*H-*imidazole-3-oxide **29** with **26** resulted in cyanide substitution with the formation of **30**, and the latter was treated with an excess of ethylmagnesium bromide (Scheme 8). The nitroxide **31** was isolated after a quenching of the reaction mixture with water and oxidation. To hydrolyze the dioxolane ring, **31** was heated to reflux in 0.5 M aqueous HCl. The resulting aldehyde **32** was reduced with sodium borohydride to the corresponding alcohol **34**, or oxidized with sodium chlorite to carboxylic acid **33** as described above for **23**. Similarly to **11**, the nitroxide **33** was converted into succinimidyl ester **35** via a reaction of in situ generated chloroanhydride with NHS.

**Figure 2:**
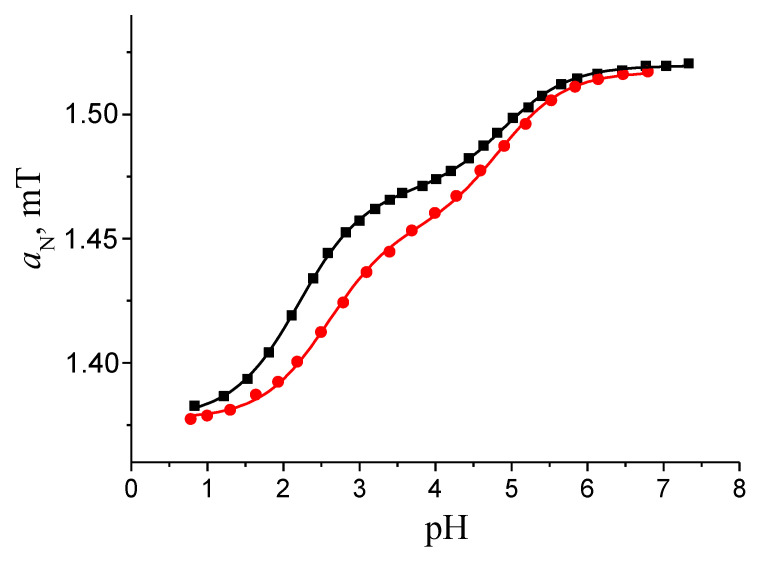
Titration curves of nitroxides **34** (•) and **35** (▪).

Titration of the nitroxides **32–35** showed a gradual monotonous increase of HFC on the nitroxide nitrogen atom by ca. 0.14 mT upon a pH change from 1.5 to 5.5 (see Figure 2, Table 2, and Supplementary Materials). The shape of the titration curve perfectly corresponded to a two-step acid–base equilibrium, and fitting with the Henderson-Hassellbalch function (Equation (2), see experimental part) gave two pK_a_ values for each nitroxide (Table 1), corresponding to the sequential protonation of the basic centers, amidine group and pyridine nitrogen.

In accordance with the general concept of basicity, the pK_a_ value of the amidine fragment should be higher than that of the pyridine one. However, according to the simulation, the protonation of the center with a more acidic pK_a_ is accompanied by a change in the hyperfine constant by 0.077–0.092 mT, which is typical of the amidine group in 4-amino-2,5-dihydroimidazol-1-oxyls, while the higher pK_a_ (4.73–4.89) corresponds to a smaller change in the hyperfine constant (0.05–0.063 mT), which may correspond to pyridine moiety protonation. Moreover, the basic pK_a_ showed minor dependence on the nature of the substituent at the exocyclic nitrogen, while the acidic pK_a_ varies from 2.58 for **34** to 2.19 for **35**. Meanwhile, a comparison of **34** and **35** shows that an increase in the electron-withdrawing character of the substituent at the exocyclic nitrogen leads to an increase of Δ*a*_N_ in the more acidic region, and a decrease of that correspondent to higher pK_a_. A comparison of the titration data for **1** and **36** [11] gives similar results. Data in the literature show that pK_a_ values for 4-amino-2,5-dihydroimidazol-1-oxyls are strongly dependent on substituents at exocyclic nitrogen and can go below four [32,33]. Thus, it is obvious that the pyridine nitrogen and amidine group in **31**–**35** have similar basicity and the contribution of different monoprotonated forms is varying depending on the electronic effect of the substituents at the exocyclic nitrogen.

## 3. Conclusions

In this paper feasibility of the approach to synthesis of pH-sensitive spin labels of 4-amino-2,5-dihydrioimidazol-1-oxyl series was once again demonstrated. We have showed that various functional groups can be easily placed in the substituents both in position 2 and to exocyclic nitrogen to make the spin probe suitable for a specific purpose. The potential of this synthetic scheme is still far from exhaustion.

## 4. Materials and Methods

### 4.1. General Information

The nitroxides and 4*H*-imidazol-3-oxides **2b**, **8**, **17** and **29** were prepared according to the literature protocols [10,11,14]. 1,1′-Carbonyldiimidazole and Ohira-Bestmann Reagent (dimethyl (1-diazo-2-oxopropyl)phosphonate, 10% solution in acetonitrile) were purchased from TCI Europe N.V. (Zwijndrecht, Belgium); 2-(2-bromoethyl)-1,3-dioxolan and 9-BBN 0.5 M solution in THF were purchased from Acros Organics B.V.B.A. (Geel, Belgium).

The IR spectra were recorded on a Bruker Vector 22 FT-IR spectrometer (Bruker, Billerica, MA, USA) in KBr pellets (1:150 ratio) or in neat samples (for oily compounds). UV spectra were acquired on a HP Agilent 8453 spectrometer (Agilent Technologies, Santa Clara, CA, USA) in ethanol solutions (concentration ~10^−^^4^ M). NMR spectra ^1^H and ^13^C were recorded on a Bruker AV-300 (300.132 and 75.467 MHz), AV-400 (400.134 and 100.614 MHz). ^1^H and ^13^C chemical shifts (δ) were internally referenced to the residual solvent peak. The nitroxides were reduced to diamagnetic compounds with PhSH [34], N_2_D_4_ [35], Zn/CF_3_COOH [36], or Zn/ND_4_Cl/D_2_O [37] prior to recording the ^1^H NMR spectra. HRMS analyses were performed with High Resolution Mass Spectrometer DFS (Thermo Electron, Brehmen, Germany). Reactions were monitored by TLC carried out using UV light 254 nm or 1% aqueous permanganate. Column chromatography was performed on silica gel 60 (70−230 mesh).

The X-ray diffraction experiment was carried out on a Bruker KAPPA APEX II (Bruker, Billerica, MA, USA) diffractometer (graphite-monochromated Mo Kα radiation). Reflection intensities were corrected for absorption by SADABS-2016 program [38]. The structure of compound **11** was solved by direct methods using the SHELXT-2014 program [39] and refined by anisotropic (isotropic for all H atoms) full-matrix least-squares method against *F*^2^ of all reflections by SHELXL-2018 [40]. The positions of the hydrogen atoms were calculated geometrically and refined in riding model. One of the geminal ethyl groups is disordered due to thermal motion at approximate ratio 3:2. Crystallographic data for **11** have been deposited at the Cambridge Crystallographic Data Centre as supplementary publication no. CCDC 2124865. Copy of the data can be obtained, free of charge, by application to CCDC, 12 Union Road, Cambridge CB21EZ, UK (Fax: +44-122-3336033 or e-mail: deposit@ccdc.cam.ac.uk; internet: www.ccdc.cam.ac.uk (accessed on 29 November 2021)). The details are shown in Supplementary Materials.

### 4.2. Synthesis

*2-(4-(Azidomethyl)phenyl)-2,5,5-triethyl-4-pyrrolidino-2,5-dihydro-1H-imidazol-1-oxyl* (**5**)

A mixture of **2b** hydrochloride [15] (320 mg, 0.82 mmol), sodium hydrocarbonate (250 mg, 3 mmol), diethyl ether (30 mL), and water (20 mL) was vigorously stirred until powder of 2b completely dissolved. The ether solution was separated and concentrated in vacuum without heating. The residue was dissolved in DMSO (5 mL), a solution of NaN_3_ (0.5 g, 7.7 mmol) was added, and the mixture was stirred at 60 °C for 10 h. The mixture was diluted with water (20 mL) and saturated solution of NaCl (50 mL) and extracted with diethyl ether. The extract was washed with saturated solution of NaCl and dried with Na_2_CO_3_, concentrated in vacuum, and the residue was separated using column chromatography on silica gel, eluent chloroform, to give **5**, yield 290 mg (95%), yellow crystals, m.p. 63–65 °C (hexane). Elemental analysis, found: C, 65.33; H, 8.01; N, 22.47; calcd. for C_20_H_29_N_6_O: C, 65.01; H, 7.91; N, 22.75%. IR (KBr) ν_max_: 2091 (N_3_), 1597 and 1574 (C=N, C=C).

*Tetraisopropyl but-3-yne-1,1-diyldiphosphonate* (**6**)

(In analogy to procedure by C. Li and C. Yuan [41]) Tetraisopropyl methylenediphosphonate (5 g, 14.5 mmol) was added dropwise to a stirred suspension of NaH (0.8 g 50% content, 16.7 mmol) in dry THF (50 mL) under argon. After hydrogen evolution ceased, propargyl bromide (1.1 mL, 14.5 mmol) was added dropwise under argon to the stirred suspension. The mixture was stirred for 3 h, then the mixture was diluted with water (50 mL) and pH was adjusted to neutral with hydrochloric acid. The mixture was extracted with diethyl ether, the extract was dried with Na_2_CO_3_ and concentrated in vacuum. The residue was separated using column chromatography on silica gel, eluent chloroform, to give **6**, yield 1.4 g (25%), colorless liquid. Elemental analysis, found: C, 50.35; H, 8.49; P, 16.10; calcd. for C_16_H_32_O_6_P_2_: C, 50.26; H, 8.44; P, 16.20%; IR (neat) ν_max_ (cm^−1^): 2122 (C≡C). ^1^H NMR (400 MHz; CDCl_3_, *δ*): 1.25 (24H, m, CH_3_), 1.93 (1H, t, *J* 2.3, ≡CH), 2.43 (1H, br tt, *J_t_*_1_ 24, *J_t_*_2_ 5.9, P−CH−P), 2.64 (2H, tdd, *J_t_* 16, *J*_d1_ 5.9, *J*_d2_ 2.3, CH_2_), 4.7 (4H, m, O−CH<); ^13^C{^1^H} NMR (150 MHz; CDCl_3_, δ): 15.87 (t, *J_P_* 4.8, CH_2_), 23.70 (dd, *J_P-1_* 5.8, *J_P-2_* 1.4, CH_3_), 24.05 (t, *J_P_* 3.5, CH_3_), 37.87 (t, *J_P_* 135.7, P_2_CH), 69.66 (s, ≡CH), 71.36 (dd, *J_P-1_* 5.7, *J_P-2_* 6.5, OCH), 81.60 (t, *J_P_* 9.7, –C≡).

*2-(4-((4-(2,2-Bis(diisopropoxyphosphoryl)ethyl)-1H-1,2,3-triazol-1-yl)methyl)phenyl)-2,5,5-triethyl-4-pyrrolidino-2,5-dihydro-1H-imidazol-1-oxyl* (**7**)

Ascorbic acid (140 mg, 0.79 mmol) was added to a mixture of **5** (241 mg, 0.65 mmol), 6 (259 mg, 0.67 mmol), EtOH (1.5 mL), H_2_O (1.5 mL), and saturated solution of CuSO_4_ in water (0.15 mL). The mixture was stirred for 2 h, then PbO_2_ (1 g, 4.17 mmol) was added, the mixture was stirred for 1 h, then the precipitate was filtered off and washed with ethanol. The combined solutions were evaporated in vacuum and the residue was separated using column chromatography on silica gel, eluent chloroform, to give **7**, yield 320 mg (65%), yellow oil. Elemental analysis, found: C, 57.23; H, 8.30; N, 10.98; P, 8.35; calcd. for C_36_H_61_N_6_O_7_P_2_: C, 57.51; H, 8.18; N, 11.18; P, 8.24%; IR (neat) ν_max_ (cm^−1^): 1595, 1576 (C=N, C=C). ^1^H NMR (300 MHz; CD_3_OD–CDCl_3_, reduced with Zn in ND_4_Cl/D_2_O, *δ*): 0.76 (3H, t, *J* 7, CH_3_), 0.90 (3H, t, *J* 7, CH_3_), 1.04 (3H, t, *J* 7, CH_3_), 1.26 (24H, m, C(CH_3_)_2_), 1.45 (2H, m, CH_2_Me), 1.83 (1H, m, CH_2_Me), 2.12 (7H, br m, CH_2_Me and C-CH_2_CH_2_-C), 2.86 (1H (partly exchanged), tt, *J_P_* 23, *J*_H_ 6, P–CH–P), 3.26 (2H, br t, *J_P_* 16.5, P_2_C−CH_2_−), 3.7 (4H, m, CH_2_−N−CH_2_), 4.69 (4H, septet, *J* 6, O−CH), 5.51 (2H, s, Ar−CH_2_), 7.32 (2H, d, *J* 8, CH Ar), 7.39 (1H, s, OH), 7.52 (2H, d, *J* 8, CH Ar), 7.69 (1H, s, N−CH=); ^1^H NMR (300 MHz; CD_3_OD–CDCl_3_, reduced with Zn/CF_3_COOH in CD_3_OD, 65 °C, *δ*): 0.85 (3H, t, *J* 7, CH_3_), 0.89 (3H, t, *J* 7, CH_3_), 1.04 (3H, t, *J* 7, CH_3_), 1.19 (6H, d, *J* 6, C(CH_3_)_2_), 1.27 (6H, d, *J* 6, C(CH_3_)_2_), 1.33 (12H, d, *J* 6, C(CH_3_)_2_), 1.49 (2H, m, CH_2_Me), 2.02 (8H, br m, CH_2_Me and C−CH_2_CH_2_−C), 2.99 (1H (partly exchanged), tt, *J_P_* 24, *J_H_* 6, P−CH-P), 3.26 (2H, m, P_2_C−CH_2_−), 3.7 (4H, m, CH_2_−N−CH_2_), 4.75 (4H, septet, *J* 6, O−CH), 5.56 (2H, s, Ar−CH_2_), 7.37 (2H, d, *J* 8, CH Ar) and 7.60 (2H, d, *J* 8, CH Ar), 7.75 (1H, s, N−CH=); ^31^P NMR (121.497 MHz; CD_3_OD-CDCl_3_, reduced with Zn in ND_4_Cl/D_2_O, *δ*): 20.44, 20.47.

*2,5,5-Triethyl-2-(4-formylphenyl)-4-pyrrolidino-2,5-dihydro-1H-imidazol-1-oxyl* (**9**)

Method A

Activated manganese dioxide (4 g, 46 mmol) was added to a stirred solution of **8** (0.4 g, 1.16 mmol) in methanol (50 mL). The mixture was stirred for 4 h, manganese oxides were filtered off through celite 281, the solvent was distilled off in vacuum and the residue was separated using column chromatography on silica gel, eluent chloroform, to give **9**, yield 340 mg (85%), yellow crystals, m.p. 84–85 °C dec. (chroloform-hexane). Elemental analysis, found: C, 70.20; H, 8.15; N, 12.31; calcd. for C_20_H_28_N_3_O_2_: C, 70.14; H, 8.24; N, 12.27%. IR (KBr) ν_max_ (cm^−1^): 1697 (C=O); 1593, 1570 (C=N, C=C); UV (EtOH) λ_max_ (log ε): 229 (4.22), 253 (4.26).

Method B

A powder of 2,2,6,6-tetramethylpiperidinium chloride (0.4 g, 2.03 mmol) was added to a solution of **8** (0.5 g, 1.5 mmol) in chloroform (10 mL) and the solution was stirred for 2 h at room temperature. The solvent was distilled off in vacuum, the residue was separated using column chromatography on silica gel, eluent chloroform, to give **9**, yield 440 mg (85%).

*2,5,5-Triethyl-2-(4-ethynylphenyl)-4-pyrrolidino-2,5-dihydro-1H-imidazol-1-oxyl* (**11**)

A solution of dimethyl (1-diazo-2-oxopropyl)phosphonate in acetonitrile (10%, 0.7 mL, 0.31 mmol) was added to a mixture of **8** (100 mg, 0.29 mmol), freshly annealed K_2_CO_3_ (84 mg, 0.61 mmol) and anhydrous methanol (5 mL). The mixture was stirred overnight, methanol was distilled off in vacuum, the residue was triturated with ethyl acetate, the precipitate was filtered off and washed with ethyl acetate, the combined solution was concentrated in vacuum and separated by column chromatography on silica gel, eluent diethyl ether–hexane 1:1 to give **11**, yield 82 mg (84%), orange crystals, m.p. 156–158 °C (hexane-ethyl acetate). Elemental analysis, found: C, 74.81; H, 7.97; N, 12.50; calcd. for C_21_H_28_N_3_O: C, 74.52; H, 8.34; N, 12.41%. IR (KBr) ν_max_ (cm^−1^): 3151 (≡C–H), 2094 (C≡C); 1587, 1554 (C=N, C=C).

*2-(4-Carboxyphenyl)-2,5,5-triethyl-4-pyrrolidino-2,5-dihydro-1H-imidazol-1-oxyl* (**12**)

A solution of **8** (0.5 g, 1.5 mmol) in CHCl_3_ (10 mL) was cooled to 0 °C and 2,2,6,6-tetramethyloxopiperidinium chloride (**10**) (0.4 g, 2.0 mmol) was added in one portion. The mixture was stirred for 2 h at 0 °C. Then 2-methylbut-2-ene (1.8 mL, 17.4 mmol) was added to reaction mixture followed by addition of a solution of NaClO_2_ (0.9 g, 9.8 mmol) and KH_2_PO_4_ (1.3 g, 9.8 mmol) in H_2_O (44 mL). The mixture was stirred for 2 h, the organic layer was separated, washed with saturated aqueous solution of Na_2_CO_3_ (3 × 20 mL) and concentrated in vacuum. The residue was separated by column chromatography on silica gel using CHCl_3_–EtOH mixture (100:16) as an eluent to give light-yellow crystals of **12**, yield 463 mg (89%), m.p. 205–207 °C (AcOEt—*i*-PrOH 10:1). Elemental analysis, found: C, 66.85; H, 7.87; N, 11.71; calcd. for C_20_H_28_N_3_O_3_: C, 67.01; H, 7.87; N, 11.72%. IR (KBr) ν_max_ (cm^−1^): 2974 (C–H), 1693 (C=O), 1591 (C=N), 1571 (C=C). UV (EtOH) λ_max_ (log ε): 232 (4.41). ^1^H NMR (400 MHz; CD_3_OD–CDCl_3_, reduced with PhSH, *δ*): 0.78 (3H, t, *J* 7.3, CH_3_), 0.85 (3H, t, *J* 7.3, CH_3_), 0.95 (3H, t, *J* 7.3, CH_3_), 1.00–1.12 (2H, m, CH_2_, Et), 1.36, 1.75 (2H, AB, CH_2_, Et), 1.85–1.99 (2H, m, CH_2_, Et), 2.00 (4H, m, 4CH_2_, Pyrr), 3.50–3.55 (4H, m, CH_2_−N−CH_2_, Pyrr), 7.66 (2H, d, *J* 8, CH Ar), 8.01 (2H, d, *J* 8, CH Ar).

*2-(4-(Ethoxycarbonyl)phenyl)-2,5,5-triethyl-4-pyrrolidino-2,5-dihydro-1H-imidazol-1-oxyl* (**14**)

Pyridine (340 μL, 4.2 mmol) was added to a suspension of **12** (0.5 g, 1.4 mmol) acid in dry CHCl_3_ (10 mL). The resulting solution was stirred at 0 °C, and SOCl_2_ (130 μL, 1.8 mmol) was added dropwise. The stirring continued for 3 h, then ethanol (1 mL, 17 mmol) was added in one portion. The mixture was stirred for 2 h, the solvent was removed in vacuum, and the residue was separated using column chromatography on silica gel, eluent CHCl_3_–EtOH 200:1, to give **14**, yield 352 mg (65%), yellow crystals, m.p. 85–90 °C (hexane). Elemental analysis, found: C, 68.60; H, 8.10; N, 10.80; calcd. for C_23_H_32_N_3_O_3_: C, 68.27; H, 8.35; N, 10.87). IR (KBr) ν_max_ (cm^−1^): 2970 (C–H), 1718 (C=O), 1593 (C=N), 1571 (C=C). UV (EtOH) λ_max_ (log ε): 231 (4.45). ^1^H NMR (300 MHz; CDCl_3_-CD_3_OD, reduced with Zn/CF_3_COOH in CD_3_OD, 65 °C, *δ*): 0.62 (3H, t, *J* 7.2, CH_3_), 0.68 (3H, t, *J* 7.4, CH_3_), 0.81 (3H, t, *J* 7.5, CH_3_), 0.88–1.12 (2H, m, CH_2_, Et), 1.17 (3H, t, *J* 7.1, CH_3_CH_2_O), 1.2, 1.60–1.84 (4H, m, CH_2_, Et_2_), 1.89 (4H, m, C−CH_2_CH_2_−C, Pyrr), 3.44 (4H, m, CH_2_–N–CH_2_, Pyrr), 4.15 (2H, q, *J* 7.1, CH_2_O), 7.44 (2H, d, *J* 8, CH Ar), 7.80 (2H, d, *J* 8, CH Ar).

*2-(4-((2,5-Dioxopyrrolidinooxy)carbonyl)phenyl)-2,5,5-triethyl-4-pyrrolidino-2,5-dihydro-1H-imidazol-1-oxyl* (**15**)

Pyridine (100 μL, 1.2 mmol) was added to a suspension of **12** (0.138 g, 0.39 mmol) in dry CHCl_3_ (5 mL), the resulting solution was stirred at 0 °C, and SOCl_2_ (30 μL, 0.4 mmol) was added dropwise. The reaction mixture was stirred for 3 h, then *N*-hydroxysuccinimide (44 mg, 0.39 mmol) was added in one portion. The mixture was stirred for 1 h, the solvent was removed in vacuum, and residue was separated using column chromatography on silica gel, eluent CHCl_3_–EtOH (100:1), to give **15**, yield 102 mg (58%), yellow crystals, m.p. 107–108 °C (Et_2_O—hexane 1:2). Elemental analysis, found: C, 63.09; H, 6.86; N, 11.92; calcd. for C_24_H_31_N_4_O_5_: C, 63.28; H, 6.86; N, 12.30%. IR (KBr) ν_max_ (cm^−1^): 2974 (C–H), 1774, 1743 (C=O), 1591 (C=N), 1571 (C=C). UV (EtOH) λ_max_ (log ε): 235 (4.41).

*2-(4-((3-Carboxypropanoyloxy)methyl)phenyl)-2,5,5-triethyl-4-pyrrolidino-2,5-dihydro-1H-imidazol-1-oxyl* (**16**)

Succinic anhydride (0.15 g, 1.5 mmol) was added to a solution of **8** (0.2 g, 0.6 mmol) in CHCl_3_ (10 mL) and the reaction mixture was heated to reflux for 2 h. The resulting solution was washed with H_2_O (10 mL), dried with Na_2_SO_4_, and the solvent was removed in vacuum. The solid residue was triturated with ether, the crystalline precipitate of **16** was filtered off and washed with diethyl ether, yield 213 mg (80%), yellow crystals, m.p. 168–169 °C dec. (Et_2_O). Elemental analysis, found: C, 64.37; H, 7.52; N, 9.48; calcd. for C_24_H_34_N_3_O_5_: C, 64.24; H, 7.71; N, 9.45%. IR (KBr) ν_max_ (cm^−1^): 2969 (C-H), 1731 (C=O ester), 1587 (C=N), 1569 (C=O carboxy). UV (EtOH) λ_max_ (log ε): 220 (4.03). ^1^H NMR (300 MHz; CDCl_3_-CD_3_OD, reduced with Zn/CF_3_COOH in CD_3_OD, 65 °C, *δ*): 0.66 (6H, m, CH_3_), 0.81 (3H, m, CH_3_), 1.00, 1.24 (2H, m, CH_2_, Et), 1.56–1.81 (4H, m, 2 × CH_2_, Et_2_), 1.89 (4H, m, C−CH_2_CH_2_−C, Pyrr), 2.44 (4H, m, CH_2_CH_2_CO_2_H), 3.44 (4H, m, CH_2_–N–CH_2_, Pyrr), 4.9 (2H, m, CH_2_O), 7.14 (2H, d, *J* 8, CH Ar), 7.31 (2H, d, *J* 8, CH Ar).

*2-Allyl-2-ethyl-5,5-dimethyl-4-(pyrrolidino)-2,5-dihydroimidazol-1-oxyl* (**18a**)

A solution of allylmagnesium bromide prepared from allyl bromide (1.69 mL, 20 mmol) and Mg (0.5 g, 20.5 mmol) in diethyl ether (15 mL) under argon was added dropwise to a stirred solution of nitrone **17** (0.83 g, 4.0 mmol) in THF (15 mL). The reaction mixture was stirred for 1 h, then water (30 mL) was added dropwise under vigorous stirring. Then manganese dioxide (5 g, 57 mmol) was added and the reaction mixture was stirred for 1 h. The manganese oxides were filtered off and the precipitate was washed with *tert*-butylmethyl ether. The organic layer was separated, the water solution was saturated with NaCl and extracted with *tert*-butylmethyl ether. The combined organic extracts were concentrated in vacuum and the residue was separated using column chromatography on Al_2_O_3_, eluent *tert*-butylmethyl ether–hexane (1:1) to give **18a**, yield 630 mg (63%), yellow crystals, m.p. 55–57 °C (hexane). Elemental analysis, found: C, 67.22; H, 10.23; N, 16.88; calcd. for C_14_H_24_N_3_O: C, 67.16; H, 9.66; N, 16.78%. IR (KBr) ν_max_ (cm^−1^): 2975 (C–H), 1645 (C=C), 1590 (C=N). UV (EtOH) λ_max_ (log ε): 225 (4.17). ^1^H NMR (400 MHz; CD_3_OD, reduced with N_2_D_4_, *δ*): 0.91 (3H, t, *J* 7, CH_3_), 1.47 (6H, d, *J* 2.2, CH_3_), 1.60–1.83 (4H, m, CH_2_, Et), 2.01 (4H, m, CH_2_–CH_2_ (pyrr)), 2.39–2.63 (2H, m, CH_2_–CH=CH_2_), 3.56 (4H, s, CH_2_–N–CH_2_), 5.08 (2H, m, CH_2_=CH), 5.97 (1H, tdd, *J_t_* 7, *J_d1_* 10.7, *J_d2_* 17.2, CH_2_=CH).

*2-Ethyl-5,5-dimethyl-2-(pent-4-enyl)-4-(pyrrolidino)-2,5-dihydroimidazol-1-oxyl* (**18b**)

A solution of pent-4-enylmagnesium bromide was prepared from 5-bromopentene (1.6 g, 12 mmol) and Mg (335 mg, 14 mmol) in THF (20 mL) under argon. This solution was added dropwise to a stirred solution of **17** (1 g, 4.8 mmol) in THF (20 mL). The reaction mixture was stirred overnight, then water (4 mL) was added dropwise under vigorous stirring. The reaction mixture was vigorously stirred in air for 1 h, then organic layer was separated, and the aqueous layer was extracted with Et_2_O–EtOH (100:1). The combined organic extracts were dried with Na_2_SO_4_, solvents were distilled off in vacuum, and the residue was separated by column chromatography on Al_2_O_3_ using hexane–CHCl_3_ mixture (2:1) as an eluent to give **18b**. Yield 931 mg (70%), yellow oil. Elemental analysis, found: C, 68.93; H, 9.80; N, 15.00; calcd. for C_16_H_28_N_3_O: C, 69.02; H, 10.14; N, 15.09%. IR (KBr) ν_max_ (cm^−1^): 2973 (C–H), 1639 (C=C), 1594 (C=N). UV (EtOH) λ_max_ (log ε): 225 (4.19). ^1^H NMR (300 MHz; CDCl_3_-CD_3_OD, reduced with Zn/CF_3_COOH in CD_3_OD, 65 °C, *δ*): 0.65 (3H, m, CH_3_, Et), 1.16 (2H, m, CH_2_, Et), 1.23 (2H, s, CH_3_), 1.32 (4H, s, CH_3_), 1.36–1.64 (4H, m, CH_2_–CH_2_–Allyl), 1.77 (2H, m, CH_2_–C=), 1.85 (4H, m, CH_2_–CH_2_–CH_2_–CH_2_), 3.23, 3.43 (4H, m, CH_2_–N–CH_2_), 4.60–4.76 (2H, m, CH_2_=), 5.48 (1H, tdd, =CH–, *J_t_* 7, *J_d1_* 10.3, *J_d2_* 17,1).

*2-Ethyl-2-(3-hydroxypropyl)-5,5-dimethyl-4-(pyrrolidino)-2,5-dihydroimidazol-1-oxyl* (**19a**)

Method A

A solution of 9-BBN in THF (0.5 M, 8 mL, 4.1 mmol) was added dropwise to a stirred solution of **18a** (400 mg, 1.6 mmol) in THF (10 mL) under argon. The reaction mixture was vigorously stirred for 4 h, then cooled to 0 °C and cold (0 °C) aqueous NaOH (20%, 10 mL) and cold (0 °C) H_2_O_2_ (30%, 3 mL) were added dropwise successively. The mixture was allowed to warm to room temperature upon stirring (ca. 2 h), organic layer was separated, dried with Na_2_CO_3,_ and the solvent was distilled off in vacuum. The residue was dissolved in CHCl_3_ (25 mL), anhydrous Na_2_CO_3_ (1 g) was added, and mixture was allowed to stand overnight in air. The solution was concentrated in vacuum and separated by column chromatography on silica gel using CHCl_3_–EtOH mixture (100:4) as an eluent to give **19a**. Yield 150 mg (35%), yellow oil. Elemental analysis, found: C, 62.53; H, 9.49; N, 15.45; calcd. for C_14_H_26_N_3_O_2_: C, 62.65; H, 9.76; N, 15.66%. IR (KBr) ν_max_ (cm^−1^): 3386 (br., OH), 1592 (C=N). UV (EtOH) λ_max_ (log ε): 225 (4.07). ^1^H NMR (400 MHz; CD_3_OD, reduced with N_2_D_4_, *δ*): 0.94 (3H, t, *J* 7.2, CH_3_, Et), 1.43 (6H, s, CH_3_), 1.53–1.92 (6H, m, CH_2_), 1.98 (4H, m, CH_2_-CH_2_-CH_2_-CH_2_), 3.52 (4H, s, CH_2_-N-CH_2_), 3.57 (2H, br. s, CH_2_O). *2-Ethyl-2-(5-hydroxypentyl)-5,5-dimethyl-4-(pyrrolidino)-2,5-dihydroimidazol-1-oxyl* (**19b**) was prepared similarly from **18b**. Yield 42%, yellow crystals, m.p. 68–73 °C (Et_2_O). Elemental analysis found: C, 65.17; H, 10.56; N, 14.08; calcd. for C_16_H_30_N_3_O_2_: C, 64.83; H, 10.20; N, 14.18%. IR (KBr) ν_max_ (cm^−1^): 3261 (br., OH), 1593 (C=N). UV (EtOH) λ_max_ (log ε): 225 (4.1). ^1^H NMR (300 MHz; CDCl_3_–CD_3_OD, reduced with Zn/CF_3_COOH in CD_3_OD, 65 °C, *δ*): 0.62 (3H, m, CH_3_, Et), 1.06 (4H, br. m, CH_2_–CH_2_–(CH_2_)_2_OH), 1.21–1.28 (8H, m, 2 × CH_3_, CH_2_, Et), 1.40 (2H, m, CH_2_–CH_2_OH), 1.57 (2H, m, >C(Et)–CH_2_), 1.79 (4H, br. m, C−CH_2_CH_2_−C, Pyrr), 3.20, 3.44 (4H, m, CH_2_–N–CH_2_, Pyrr), 3.26 (2H, t, *J* 6.5, CH_2_O).

Method B

Sodium borohydride (60 mg, 1.6 mmol) was added portionwise to a stirred solution of **24** (400 mg, 1.5 mmol) in EtOH (10 mL) at 0 °C. The reaction was controlled with TLC, Silufol UV-254, eluent CHCl_3_–EtOH (25:1). Inorganic residue was filtered off, the solution was distilled off in vacuum, and the residue separated by column chromatography as described above to give **19a**. Yield 309 mg (72%).

*2-(3-(1H-Imidazole-1-carbonyloxy)propyl)-2-ethyl-5,5-dimethyl-4-(pyrrolidino)-2,5-dihydro-1H-imidazol-1-oxyl* (**20a**)

Carbonyldiimidazole (80 mg, 0.49 mmol) was added to a solution of alcohol **19a** (114 mg, 0.43 mmol) in dry CHCl_3_ (5 mL) and the mixture was allowed to stand for 24 h. The solution was washed with brine, dried with Na_2_SO_4_, and concentrated in vacuum. The residue was separated by column chromatography on silica gel using CHCl_3_–EtOH mixture (100:2) as an eluent, producing **20a** as yellow oil. Yield 139 mg (90%). Elemental analysis, found: C, 59.69; H, 7.72; N, 19.45; calcd. for C_18_H_28_N_5_O_3_: C, 59.65; H, 7.79; N, 19.32%. IR (KBr) ν_max_ (cm^−1^): 1760 (C=O), 1592 (C=N). UV (EtOH) λ_max_ (log ε): 223 (4.17). *2-(5-(1H-Imidazole-1-carbonyloxy)pentyl)-2-ethyl-5,5-dimethyl-4-(pyrrolidino)-2,5-dihydro-1H-imidazol-1-oxyl* (**20b**) was prepared similarly, yield 80%, yellow oil. Elemental analysis, found: C, 61.30; H, 8.26; N, 17.70; cacld. for C_20_H_32_N_5_O_3_: C, 61.51; H, 8.26; N, 17.93%. IR (KBr) ν_max_ (cm^−1^): 1762 (C=O), 1593 (C=N). UV (EtOH) λ_max_ (log ε): 226 (3.99).

*2-(3-(3-(Diethylamino)propylcarbamoyloxy)propyl)-2-ethyl-5,5-dimethyl-4-(pyrrolidino)-2,5-dihydro-1H-imidazol-1-oxyl* (**21**)

**Figure 3:**
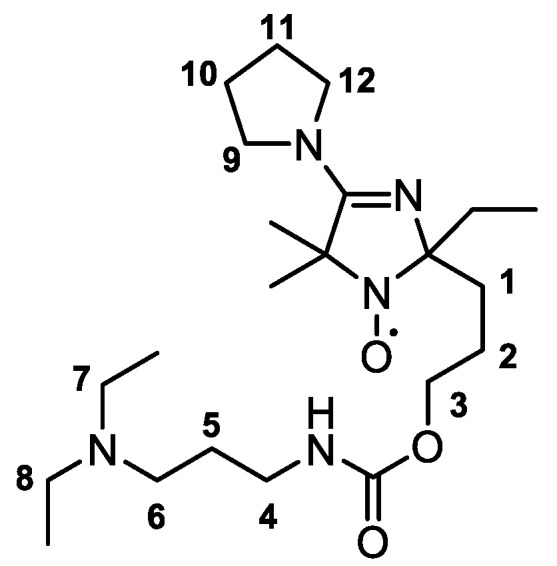
The structure of nitroxide **21**.

*N,N-*Diethyl-1,3-diaminopropane (50 mg, 0.38 mmol) was added to a solution of **20a** (126 mg, 0.35 mmol) in dry Et_2_O (5 mL), and mixture was allowed to stay for 24 h. The solution was concentrated in vacuum, and the residue was separated by column chromatography on Al_2_O_3_ using CHCl_3_ as an eluent to give **21** (Figure 3). Yield 82 mg (60%), yellow oil. Elemental analysis, found: C, 62.21; H, 10.01; N, 16.51; calcd. for C_22_H_42_N_5_O_3_: C, 62.23; H, 9.97; N, 16.49%. IR (KBr) ν_max_ (cm^−1^): 1718 (C=O), 1593 (C=N). UV (EtOH) λ_max_ (log ε): 225 (4.19). ^1^H NMR (300 MHz; CDCl_3_-CD_3_OD, reduced with Zn/CF_3_COOH in CD_3_OD, 65 °C, *δ*): 0.75 (3H, t, *J* 7.2, CH_3_), 1.11 (6H, t, *J* 7.3, 2 × CH_3_) 1.33 (6H, br., CH_3_), 1.40 (6H, br., CH_3_), 1.43–1.79 (8H, m, CH_3_CH_2_C, ^1^CH_2_, ^2^CH_2_, ^5^CH_2_,), 1.90 (4H, br. m, ^10^CH_2_, ^11^CH_2_), 2.87–3.03 (8H, m, ^4^CH_2_, ^6^CH_2_, ^7^CH_2_, ^8^CH_2_), 3.36, 3.52 (4H, m, ^9^CH_2_, ^12^CH_2_), 3.84 (2H, m, CH_2_O).

*2-(3-Carboxypropyl)-2-ethyl-5,5-dimethyl-4-(pyrrolidino)-2,5-dihydro-1H-imidazol-1-oxyl* (**22**)

Osmium tetroxide (30 mg, 0.4 mmol) and oxone (1.77 g, 5.8 mmol) were added successively to a solution of **18b** (400 mg, 1.4 mmol) in DMF (20 mL) and the mixture was stirred for 3 h. A powder of Na_2_SO_3_ (10 g, 63 mmol) was added in one portion. Inorganic precipitate was filtered off and washed with EtOH, the combined solution was evaporated to dryness in vacuum and the residue was separated by column chromatography on silica gel using EtOH as an eluent to give **22**, yield 90 mg (21%), yellow oil. M^+^ (cacld./found) 296.1969/296.1972. IR (neat) ν_max_ (cm^−1^): 2977 (C-H), 1664 (C=O), 1592 (C=N). λ_max_ (EtOH)/nm: 225 (lgε 4.19).

*2-(2-(1,3-Dioxolan-2-yl)ethyl)-2-ethyl-5,5-dimethyl-4-(pyrrolidin-1-yl)-2,5-dihydro-1H-imidazol-1-oxyl* (**23**)

A solution of 2-(1,3-dioxolan-2-yl)ethylmagnesium bromide was prepared from 2-(2-bromoethyl)-1,3-dioxolan (4.3 g, 24 mmol) and Mg (670 mg, 28 mmol) in 20 mL THF under a stream of argon. This solution was added dropwise to a stirred solution of nitrone **17** (850 mg, 4 mmol) in 20 mL Et_2_O and 6 mL THF. The reaction mixture was stirred overnight, then water (5 mL) was added dropwise under vigorous stirring. The reaction mixture was allowed to air for 1 h, then organic layer was separated, inorganic residue was quenched with Et_2_O–EtOH (100:1). An isolated organic layer was dried over Na_2_SO_4_, solvents were removed in vacuum. The residue was separated using column chromatography on Al_2_O_3_ using CHCl_3_ as an eluent to give **23**, yield 1.13 g (90%), yellow oil. Elemental analysis, found: C, 62.08; H, 9.21; N, 13.43; calcd for C_16_H_28_N_3_O_3_: C, 61.91; H, 9.09; N, 13.54%. IR (KBr) ν_max_ (cm^−1^): 2972 (C-H), 1593 (C=N), 1143 (C–O). λ_max_ (EtOH)/nm: 225 (lgε 3.90).

*2-Ethyl-5,5-dimethyl-2-(3-oxopropyl)-4-(pyrrolidin-1-yl)-2,5-dihydro-1H-imidazol-1-oxyl* (**24**)

A solution of oxalic acid (180 mg, 2 mmol) in water (6 mL) was added to a solution of nitroxide 23 (250 mg, 0.8 mmol) in EtOH (4 mL). The reaction mixture was stirred for 3 h under reflux, then ethanol was removed in vacuum, saturated aqueous KHCO_3_ (10 mL) was added to a residue. The product was extracted with CHCl_3_–*i*-PrOH mixture (50:1) (3 × 15 mL). An isolated organic layer was dried over Na_2_SO_4_, the solvents were removed in vacuum, the residue was separated using column chromatography on silica gel using CHCl_3_-EtOH mixture (50:1) as an eluent to give **24**, yield 161 mg (75%), yellow oil. Elemental analysis, found: %: C, 63.08; H, 9.18; N, 15.63; calcd. for C_14_H_24_N_3_O_2_: C, 63.13; H, 9.08; N, 15.78. IR (neat) ν_max_ (cm^−1^): 2972 (C-H), 1720 (C=O), 1593 (C=N). λ_max_ (EtOH)/nm: 225 (lgε 4.16).

*2-(2-Carboxyethyl)-2-ethyl-5,5-dimethyl-4-(pyrrolidin-1-yl)-2,5-dihydro-1H-imidazol-1-oxyl* (**25**)

Trimethylethylene (1 mL, 9.0 mmol) was added to a cooled (0 °C) solution of aldehyde **24** (200 mg, 0.8 mmol) in 10 mL CH_3_CN followed by addition of a solution of NaClO_2_ (480 mg, 5.3 mmol) and KH_2_PO_4_ (710 mg, 5.3 mmol) in H_2_O (20 mL). Progress of the reaction was monitored by TLC (silica gel, CHCl_3_–EtOH (50:1), developing with 1% aq KMnO_4_). CH_3_CN was removed in vacuum, the product was extracted from water by CHCl_3_–*i*-PrOH mixture (100:1) (5 × 15 mL). An isolated organic layer was dried over Na_2_SO_4_, the solvents were removed in vacuum, the residue was separated using column chromatography on silica gel using CHCl_3_–EtOH mixture (5:2) as an eluent to give **25**, yield 121 mg (57%), yellow oil, M^+^ (calcd./found) 282.1812/282.1811. IR (neat) ν_max_ (cm^−1^): 2973 (C–H), 1729 (C=O), 1591 (C=N). λ_max_ (EtOH)/nm: 223 (lgε 4.04). ^1^H NMR (300 MHz; CDCl_3_-CD_3_OD, reduced with Zn/CF_3_COOH in CD_3_OD, 65 °C, *δ*) 0.75 (3H, m, CH_3_, Et), 0.98–1.14 (2H, m, CH_2_, Et), 1.34, 1.40 (6H, m, 2 × CH_3_), 1.47–1.61 (2H, m, CH_2_CH_2_CO_2_H), 1.91 (4H, m, CH_2_–CH_2_–CH_2_–CH_2_, Pyrr), 2.14–2.27 (2H, m, CH_2_CH_2_CO_2_H), 3.36, 3.55 (4H, m, CH_2_–N–CH_2_, Pyrr).

*1-(4-(1,3-Dioxolan-2-yl)phenyl)-N-methylmethanamine* (**26**)

p-Toluene sulfonic acid monohydrate (0.5 g, 74.6 mmol) was added to a solution of terephthalic aldehyde **27** (10 g, 74.6 mmol) in 175 mL PhCH_3_. Water was distilled off with Dean-Stark tube. The reaction mixture was then quenched with aqueous NaHCO_3_, dried over Na_2_CO_3_, the solvent was remove in vacuum, and residue was dissolved in methanol saturated with methylamine (20 mL). The resulting solution was added to the previously maintained under vigorous stirring for 10 min in a solution of Ti(O*i*-Pr)_4_ (14 mL, 47 mmol) in methanol saturated with methylamine (30 mL). The mixture was stirred for 5 h, then NaBH_4_ (1.34 g, 33.6 mmol) was added portionwise, and mixture was stirred for 2 h. Water (7 mL) was added dropwise, solvents were removed in vacuum, brine was added to a residue, and the product was extracted by ether. Organic layer was dried over NaOH. Residue was separated using column chromatography on silica gel using Et_2_O–EtOH mixture (10:1) as an eluent, yield 12.24 g (85%), colorless oil. Elemental analysis, found: C, 67.84; H, 7.91; N, 6.94; calcd for C_11_H_15_NO_2_: C, 68.37; H, 7.82; N, 7.25%. ^1^H NMR (300 MHz; CDCl_3_, *δ*): 2.38 (3H, s, CH_3_), 3.70 (2H, s, N–CH_2_), 3.95–4.09 (4H, m, –O–CH_2_–CH_2_–O–), 5.75 (1H, s, O–CH–O), 7.29, 7.38 (4H, AA’BB’, C_6_H_4_)), ^13^C NMR (75 MHz; CDCl_3_, *δ*): 35.77 (N–CH_3_), 55.56 (N–CH_2_), 65.06 (O–CH_2_–CH_2_–O), 103.44 (O–CH–O), 126.31 (CH–C–CH_2_NHCH_3_), 127.89 (CH–C–CH), 136.32 (C–CH_2_NHCH_3_), 141.10 (C–CH). IR (neat) ν_max_ (cm^−1^): 3325 (N–H), 1082 (O–C–O). λ_max_ (EtOH)/nm: 210 (logε 3.94), 260 (logε 2.36).

*5-((4-(1,3-Dioxolan-2-yl)benzyl)(methyl)amino)-4,4-dimethyl-2-(pyridin-4-yl)-4H-imidazole 3-oxide* (**30**)

1-(4-(1,3-Dioxolan-2-yl)phenyl)-*N*-methylmethanamine **26** (6.72 g, 34.8 mmol) was added to a solution of 5-cyano-4,4-dimethyl-2-(pyridin-4-yl)-4*H*-imidazole 3-oxide **29** (2.98 g, 13.9 mmol) in THF (25 mL) and the mixture was allowed to stand at r.t. for 24 h. The solvent was removed in vacuum, residue was triturated with ether and crystallization from CH_3_CN to give **30**, yield 3.97 g (75%), dirty-yellow crystals, m.p. 160 °C (dec.). Elemental analysis, found: C, 65.81; H, 6.25; N, 14.41; calcd for C_21_H_24_N_4_O_3_: C, 66.30; H, 6.36; N, 14.73%. ^1^H NMR (400 MHz; CDCl_3_, *δ*) 1.66 (6H, s, 2 × CH_3_), 3.06 (3H, s, N–CH_3_), 3.86–4.16 (4H, m, O–CH_2_–CH_2_–O), 4.77 (2H, br. s, N–CH_2_–Ar), 5.75 (1H, s, O–CH–O), 7.26, 7.46 (4H, AA’BB’, C_6_H_4_)), 8.46, 8.70 (4H, AA’BB’, Py). ^13^C NMR (75 MHz; CDCl_3_, *δ*) 21.50 (2 × Me), 35.22 (N–CH_3_), 53.11 (N–CH_2_), 65.00 (O–CH_2_–CH_2_–O), 75.73 (Me_2_C), 102.82 (O–CH–O), 121.00 (3,5–Py), 126.77 (br., CH (C_6_H_4_)), 133.86 (Py, *i*), 136.36 (C–CH_2_NCH_3_), 137.47 (C–CH), 144.67 (C=N→O), 149.82 (2,6-Py), 172.33 (C=N). IR (KBr) ν_max_ (cm^−1^): 1597 (C=N), 1082 (O–C–O). λ_max_ (EtOH)/nm: 263 (logε 4.30), 389 (logε 3.78).

*4-((4-(1,3-Dioxolan-2-yl)benzyl)(methyl)amino)-2-ethyl-5,5-dimethyl-2-(pyridin-4-yl)-2,5-dihydro-1H-imidazol-1-oxyl* (**31**)

A solution of ethylmagnesium bromide was prepared from ethyl bromide (2.73 g, 25 mmol) and Mg (630 mg, 26 mmol) in 35 mL Et_2_O under a stream of argon. This solution was added dropwise to a stirred solution of nitrone **27** (1 g, 2.6 mmol) in 15 mL THF. The reaction mixture was allowed to stand for 1 h. Then water (3 mL) was added dropwise under vigorous stirring followed by MnO_2_ (3 g, 34.5 mmol) addition. Progress of the reaction was monitored by TLC (silica gel, CHCl_3_–EtOH (100:3), developing with 1% aq. KMnO_4_). The mixture was stirred vigorously for 2 h, the oxidant was filtered off and the residue was washed by CHCl_3_ and MeOH. The solvent from filtrate was removed in vacuum and the residue was separated by column chromatography on silica gel using CHCl_3_-EtOH (100:3) as an eluent. The product **31** was isolated as a hydrochloride. Yield 797 mg (68%), yellow oil. Elemental analysis, found: C, 62.18; H, 6.83; N, 12.48; Cl, 6.70; calcd. for C_23_H_30_ClN_4_O_3_: C, 61.94; H, 6.78; N, 12.56; Cl, 6.95%. IR (neat) ν_max_ (cm^−1^): 1593 (C=N), 1082 (O–C–O). λ_max_ (EtOH)/nm: 216 (logε 4.34).

*2-Ethyl-4-((4-formylbenzyl)(methyl)amino)-5,5-dimethyl-2-(pyridin-4-yl)-2,5-dihydro-1H-imidazol-1-oxyl* (**32**)

A solution of nitroxide **30** (1.8 g, 4.4 mmol) in 15 mL 0.5 M aq. HCl was refluxed for 4 h, then Na_2_CO_3_ added to the end of gas evolution. A product was extracted by mixture of 20 mL CHCl_3_ + 1 mL *i*-PrOH three times, organic layer was dried over Na_2_CO_3_, the solvents were removed in vacuum, and the nitroxide **29** was isolated from the residue by column chromatography on silica gelusing CHCl_3_ as an eluent. Yield 1.14 g (71%), yellow oil. Elemental analysis, found: C, 68.73; H, 6.88; N, 14.92; calcd. for C_21_H_25_N_4_O_2_: C, 69.02; H, 6.90; N, 15.33%. IR (KBr) ν_max_ (cm^−1^): 1701 (C=O), 1593 (C=N). λ_max_ (EtOH)/nm: 252 (logε 4.29).

*4-((4-Carboxybenzyl)(methyl)amino)-2-ethyl-5,5-dimethyl-2-(pyridin-4-yl)-2,5-dihydro-1H-imidazol-1-oxyl* (**33**)

Trimethylethylene (1.33 g, 19.2 mmol) was added to a cooled (0 °C) solution of aldehyde **29** (583 mg, 1.6 mmol) in 20 mL CHCl_3_ followed by addition of a solution of NaClO_2_ (1.02 g, 11.2 mmol) and KH_2_PO_4_ (1.5 g, 11.2 mmol) in H_2_O (50 mL). Progress of the reaction was monitored by TLC (silica gel, CHCl_3_–EtOH (50:1), developing with 1% aq. KMnO_4_). The organic layer was separated, the product was extracted from water by CHCl_3_—*i*-PrOH mixture (20:1) (2 × 20 mL). A combined organic extracts were washed with brine, dried over Na_2_SO_4_, the solvents were removed in vacuum, the residue was separated using column chromatography on silica gel using AcOEt–EtOH mixture (10:1) as an eluent. Yield 285 mg (47%), yellow crystals, compound **33** was isolated as a crystal solvate 3 (**33**) × 2 EtOH (ether–EtOH 100:2), m.p. 204 °C (dec.). Elemental analysis, found: C, 65.12; H, 6.42; N, 13.12; calcd. for C_67_H_87_N_12_O_11_: C, 65.08; H, 7.09; N, 13.59%. IR (KBr) ν_max_ (cm^−1^): 2474 (O–H), 1708 (C=O), 1597 (C=N), λ_max_ (EtOH)/nm: 242 (logε 4.20).

*2-Ethyl-4-((4-(hydroxymethyl)benzyl)(methyl)amino)-5,5-dimethyl-2-(pyridin-4-yl)-2,5-dihydro-1H-imidazol-1-oxyl* (**34**)

NaBH_4_ (54 mg, 1.4 mmol) was added portionwise to a cooled (0 °C) solution of aldehyde **32** (511 mg, 1.4 mmol) in EtOH (20 mL). The reaction mixture was stirred until the reaction was complete (TLC, Silufol UV-254, eluent AcOEt). The solvent was removed in vacuum, the residue was separated using column chromatography on silica gel using AcOEt as an eluent. Yield 308 mg (60%), yellow crystals, compound **34** was isolated as a crystal solvate 2 (**34**) × 3 H_2_O (ether), m.p. 147–148 °C. Elemental analysis, found: C, 66.31; H, 7.12; N, 14.55; calcd. for C_63_H_85_N_12_O_8_: C, 66.47; H, 7.53; N, 14.76%. IR (KBr) ν_max_ (cm^−1^): 3178 (O-H), 1595 (C=N). λ_max_ (EtOH)/nm: 220 (logε 4.30). ^1^H NMR (400 MHz; CD_3_OD–CDCl_3_, reduced with Zn/CF_3_COOH in CD_3_OD, 65 °C, *δ*): 1.03 (3H, t, *J* 7.2, CH_3_ Et_2_), 1.29 (3H, br s, CH_3_), 1.79 (2H, q, *J* 7.2, CH_2_), 1.90 (3H, s, CH_3_), 3.20 (3H, br s, NCH_3_), 4.65 (2H, s CH_2_OH), 4.97 (2H, br s, N–CH_2_), 7.26 (2H, m, Ar), 7.46 (2H, m, Ar), 7.94 (2H, d, *J* 6.5, Py), 8.73 (2H, d, *J* 6.5, Py)

*4-((4-(((2,5-Dioxopyrrolidin-1-yl)oxy)carbonyl)benzyl)(methyl)amino)-2-ethyl-5,5-dimethyl-2-(pyridin-4-yl)-2,5-dihydro-1H-imidazol-1-oxyl* (**35**)

Pyridine (240 μL, 3 mmol) was added to a cooled (0 °C) suspension of acid **33** (228 mg, 0.6 mmol) in 10 mL of dry CHCl_3_ followed by addition of SOCl_2_ (90 μL, 1.2 mmol). The reaction mixture was vigorously stirred for 3 h, then *N*-hydroxysuccinimide (138 mg, 1.2 mmol) was added and the mixture was allowed to stand for 24 h. The solvents were then removed in vacuum, residue was separated using column chromatography on silica gel using CHCl_3_–EtOH mixture (100:2) as an eluent to give **35**, yield 123 mg (40%), yellow crystals, compound **35** was isolated as a hydrochloride (hexane), m.p. 58 °C (dec.). Elemental analysis, found: C, 58.42; H, 5.47; N, 13.25; Cl, 6.56; calcd for C_25_H_29_ClN_5_O_5_: C, 58.31; H, 5.68; N, 13.60; Cl, 6.88%. IR (KBr) ν_max_ (cm^−1^): 2976 (C-H), 1770 (O=C-N-C=O), 1741 (C=O), 1593 (C=N). λ_max_ (EtOH)/nm: 239 (logε 4.25)

### 4.3. EPR Experiments

EPR experiments were performed on X-band EPR (9.8 GHz) spectrometer Bruker ER-200D. Titrations of the radicals (~0.2 mM) were performed in a buffer mixture of acetate-phosphate-borate (0.5 mM of each) in a pH range of 2–10 starting from the acidic value. Small aliquots of NaOH solution were used for titration to a higher pH. The observed hfi constants were measured as a distance between low field and central lines of nitroxide triplet spectra.

Single proton exchange:

$$R\cdot +H^{+}⇄R\cdot H^{+};\;pK_{a}$$

was fitted with single pK_a_ titration curve function:

$$a_{N\;}\left(pH\right)=\frac{a_{N}\left(R\cdot \right)+a_{N}\left(R\cdot H^{+}\right)\times 10^{pK_{a}-pH}}{1+10^{pK_{a}-pH}}$$

Double proton exchange implies the serial protonation,

$$R\cdot +H^{+}⇄R\cdot H^{+};\;pK_{a1}$$

$$R\cdot H^{+}+H^{+}⇄\;R\cdot H_{2}^{2+};\;pK_{a2}$$

which results in the double pK_a_ titration curve function:

$$a_{N\;}\left(pH\right)=\frac{a_{N}\left(R\cdot \right)+a_{N}\left(R\cdot H^{+}\right)\times 10^{pK_{a1}-pH}+a_{N}\left(R\cdot H_{2}{}^{2+}\right)\times 10^{pK_{a1}-pH}\times 10^{pK_{a2}-pH}}{1+10^{pK_{a1}-pH}+10^{pK_{a1}-pH}\times 10^{pK_{a2}-pH}}$$

EPR settings. Microwave power, 5 mW; modulation amplitude, 0.08–0.12 mT.

SD for pK_a_, 0.05; for hfc, 0.005 mT.

## Figures and Tables

**Table 1:** Titration data for the pH-sensitive niroxides: HFC constants for protonated and unprotonated forms, changes in hyperfine splitting between these forms, Δ*a*_N,_, and pK_a_ values.

Nitroxide	*a*_N_, mT		Δ*a*_N_, mT	pK_a_
	R·H^+^	R·		
**5**	1.434	1.539	0.105	6.42
**7**	1.429	1.531	0.102	6.24
**8**	1.424	1.529	0.105	6.54
**11**	1.436	1.539	0.103	6.21
**12**	1.417	1.522	0.105	6.50
**15**	1.422	1.531	0.109	6.49
**16**	1.466	1.575	0.109	6.64
**18a**	1.479	1.586	0.107	7.14
**18b**	1.468	1.565	0.097	7.20
**19a**	1.493	1.589	0.096	7.25
**19b**	1.472	1.567	0.095	7.28
**20a**	1.459	1.555	0.096	6.95
**20b**	1.448	1.545	0.097	7.24 ± 0.05
**21**	1.472	1.565	0.093	7.19
**22**	1.468	1.562	0.094	7.64
**23**	1.471	1.569	0.098	7.09
**24**	1.434	1.529	0.095	6.81 ± 0.05
**25**	1.482	1.587	0.105	7.50

**Table 2:** Titration data for the pH-sensitive niroxides: HFC constants for double protonated, monoprotonated, and unprotonated forms, changes in hfi splitting, Δ*a*_N_, between these forms, pK values.

Nitroxide	*a*_N_, mT			Δ*a*_N_, mT	pK
	R·H_2_^2+^	R·H^+^	R		
**32**	1.376	1.460	1.514	0.084 0.054	2.24 ± 0.02 4.73 ± 0.02
**33**	1.377	1.457	1.516	0.080 0.059	2.36 ± 0.02 4.86 ± 0.02
**34**	1.377	1.454	1.517	0.077 0.063	2.58 ± 0.02 4.85 ± 0.03
**35**	1.377	1.469	1.519	0.092 0.050	2.20 ± 0.01 4.89 ± 0.01

## Data Availability

Data are shown within the article or in supplementary materials. Crystallographic data for **11** have been deposited at the Cambridge Crystallographic Data Centre as supplementary publication no. CCDC 2124865. Copy of the data can be obtained, free of charge, by application to CCDC, 12 Union Road, Cambridge CB21EZ, UK (fax: +44-122-3336033 or e-mail: deposit@ccdc.cam.ac.uk; internet: www.ccdc.cam.ac.uk (accessed on 29 November 2021)).

## Supplementary Materials

The following are available online at https://www.mdpi.com/article/10.3390/gels8010011/s1, Figure S1: “The molecular structure of 2,5,5-triethyl-2-(4-ethynylphenyl)-4-pyrrolidino-2,5-dihydro-1*H*-imidazol-1-oxyl (**11**)”, IR and NMR spectra of synthesized compounds, titration curves of pH-sensitive nitroxides.

## References

[1] Voinov M.A., Smirnov A.I. (2010). Spin labels and spin probes for measurements of local pH and electrostatics by EPR. Electron Paramagn. Reson..

[2] Kovaleva E., Molochnikov L., Likhtenshtein G.I., Yamauchi J., Nakatsuji S., Smirnov A., Tamura R. (2008). pH-Sensitive Nitroxide Radicals for Studying Inorganic and Organo-Inorganic Materials and Systems. Nitroxides: Applications in Chemistry, Biomedicine and Material Science.

[3] Védrine J.C. (2015). Acid–base characterization of heterogeneous catalysts: An up-to-date overview. Res. Chem. Intermed..

[4] Khramtsov V.V., Volodarsky L.B., Berliner L.J. (1998). Use of imidazoline nitroxides in studies of chemical reactions: ESR measurements of the concentration and reactivity of protons, thiols and nitric oxide. Biological Magnetic Resonance.

[5] Smirnova T.I., Voinov M.A., Smirnov A. (2009). Spin Probes and Spin Labels. Encyclopedia of Analytical Chemistry: Applications, Theory and Instrumentation.

[6] Khramtsov V., Marsh D., Weiner L., Reznikov V. (1992). The application of pH-sensitive spin labels to studies of surface potential and polarity of phospholipid membranes and proteins. Biochim. Biophys. Acta.

[7] Kovaleva E.G., Molochnikov L.S., Tambasova D., Marek A., Chestnut M., Osipova V.A., Antonov D.O., Kirilyuk I.A., Smirnov A. (2020). Electrostatic Properties of Inner Nanopore Surfaces of Anodic Aluminum Oxide Membranes upon High Temperature Annealing Revealed by EPR of pH-sensitive Spin Probes and Labels. J. Membr. Sci..

[8] Antonov D.O., Tambasova D.P., Shishmakov A.B., Kirilyuk I.A., Kovaleva E.G. (2021). Acidic and Electrosurface Properties of Binary TiO_2_–SiO_2_ Xerogels Using EPR of pH-Sensitive Nitroxides. Gels.

[9] Khramtsov V.V., Vainer L.M. (1988). Photon Transfer Reactions in Free Radicals. Spin pH Probes. Russ. Chem. Rev..

[10] Kirilyuk I.A., Shevelev T.G., Morozov D.A., Khromovskih E.L., Skuridin N.G., Khramtsov V.V., Grigor’ev I.A. (2003). Grignard Reagent Addition to 5-Alkylamino-4H-Imidazole 3-Oxides: Synthesis of New pH-Sensitive Spin Probes. Synthesis.

[11] Kirilyuk I.A., Bobko A.A., Khramtsov V.V., Grigor’ev I.A. (2005). Nitroxides with two pK values—Useful spin probes for pH monitoring within a broad range. Org. Biomol. Chem..

[12] Potapenko D.I., Foster M.A., Lurie D.J., Kirilyuk I.A., Hutchison J.M.S., Grigor’ev I.A., Bagryanskaya E.G., Khramtsov V.V. (2006). Real-time monitoring of drug-induced changes in the stomach acidity of living rats using improved pH-sensitive nitroxides and low-field EPR techniques. J. Magn. Reson..

[13] Woldman Y.Y., Semenov S.V., Bobko A.A., Kirilyuk I.A., Polienko J.F., Voinov M.A., Bagryanskaya E.G., Khramtsov V.V. (2009). Design of liposome-based pH sensitive nanoSPIN probes: Nano-sized particles with incorporated nitroxides. Analyst.

[14] Bobko A.A., Eubank T.D., Voorhees J.L., Efimova O.V., Kirilyuk I.A., Petryakov S., Trofimov D.G., Marsh C.B., Zweier J.L., Grigor’ev I.A. (2012). In Vivo Monitoring of pH, Redox Status, and Glutathione Using L-Band EPR for Assessment of Therapeutic Effectiveness in Solid Tumors. Magn. Reson. Med..

[15] Kovaleva E.G., Molochnikov L.S., Stepanova D.P., Pestov A.V., Trofimov D.G., Kirilyuk I.A., Smirnov A.I. (2017). Interfacial electrostatic properties of hydrated mesoporous and nanostructured alumina powders by spin labeling EPR. Cell BioChem. Biophys..

[16] Smirnov A., Voinov M., Kirilyuk I. (2009). Spin-labeled pH-sensitive phospholipids for interfacial pKa determination: Synthesis and characterization in aqueous and micellar solutions. J. Phys. Chem. B..

[17] Voinov M.A., Scheid C.T., Kirilyuk I.A., Trofimov D.G., Smirnov A.I. (2017). IKMTSL-PTE, a Phospholipid-Based EPR Probe for Surface Electrostatic Potential of Biological Interfaces at Neutral pH: Effects of Temperature and Effective Dielectric Constant of the Solvent. J. Phys. Chem. B.

[18] Kálai T., Hubbell W.L., Hideg K. (2009). Click Reactions with Nitroxides. Synthesis.

[19] Jakobsen U., Shelke S.A., Vogel S., Sigurdsson S.T. (2010). Site-Directed Spin-Labeling of Nucleic Acids by Click Chemistry: Detection of Abasic Sites in Duplex DNA by EPR Spectroscopy. J. Am. Chem. Soc..

[20] Skarpos H., Osipov S.N., Vorob’eva D.V., Odinets I.L., Lork E., Röschenthaler G.-V. (2007). Synthesis of functionalized bisphosphonates via click chemistry. Org. Biomol. Chem..

[21] Shelke S.A., Sigurdsson S.T. (2011). Site-Directed Nitroxide Spin Labeling of Biopolymers. Structural Information from Spin-Labels and Intrinsic Paramagnetic Centres in the Biosciences.

[22] Ur G., Kálai T., Balog M., Bognár B., Gulyás-Fekete G., Hideg K. (2015). Synthesis of New Pyrroline Nitroxides with Ethynyl Functional Group. Synthetic Commun..

[23] Bal B.S., Childers W.E., Pinnick H.W. (1981). Oxidation of α,β-unsaturated aldehydes. Tetrahedron.

[24] Hopkins E., Sanvictores T., Sharma S. (2021). Physiology, Acid Base Balance. StatPearls.

[25] Hideg K., Lex L. (1987). Synthesis of new 2-mono- and 2,5-di-functionalized pyrrolidin-1-oxyl spin labels. J. Chem. Soc. Perkin Trans..

[26] Kovaleva E.G., Molochnikov L.S., Golovkina E.L., Hartmann M., Kirilyuk I.A., Grigoriev I.A. (2015). Electrical potential near hydrated surface of ordered mesoporous molecular sieves assessed by EPR of molecular pH-probes. Micropor. Mesopor. Mater..

[27] Kovaleva E.G., Molochnikov L.S., Parshina E.V., Shishmakov A.B., Mikushina Y.u.V., Kirilyuk I.A., Grigor’ev I.A. (2014). Effect of the surface charge on the complexing and catalytic properties of Cu^2+^-containing composite materials based on zirconia and powdered cellulose. Russ. J. Phys. Chem. B.

[28] Kovaleva E.G., Molochnikov L.S., Golovkina E.L., Hartmann M., Kirilyuk I.A., Grigor’ev I.A. (2013). Dynamics of pH-sensitive nitroxide radicals in water adsorbed in ordered mesoporous molecular sieves by EPR Spectroscopy. Micropor. Mesopor. Mat..

[29] Mekhaev A.V., Pestov A.V., Molochnikov L.S., Kovaleva E.G., Pervova M.G., Yaltuk Y.u.G., Grigor’ev I.A., Kirilyuk I.A. (2011). Structure and Characteristics of Chitosan Cobalt-Containing Hybrid Systems, the Catalysts of Olefine Oxidation. Russ. J. Phys. Chem. A.

[30] Mekhaev A.V., Pestov A.V., Molochnikov L.S., Kovaleva E.G., Yatluk Y.u.G., Grigor’ev I.A., Kirilyuk I.A. (2011). Investigation of the Structure of Chitosan Hybrid Systems by pH-Sensitive Nitroxyl Radical. Russ. J. Phys. Chem. A.

[31] Parshina E.V., Molochnikov L.S., Kovaleva E.G., Shishmakov A.B., Mikushina Y.V., Kirilyuk I.A., Grigor’ev I.A. (2011). Medium acidity and catalytic properties of composite materials based on silica and titania and powder cellulose in the presence of Cu2+ ions. Russ. J. Phys. Chem. A.

[32] Polienko J.F., Schanding T., Gatilov Y.V., Grigor’ev I.A., Voinov M.A. (2008). Studies toward the synthesis of 4-(2-R-ethyl)amino-2,2,5,5-tetramethyl-3-imidazoline 1-oxyls. Nucleophilic substitution of bromide in the N-alkyl chain of the 1,2,4-oxadiazol-2-one precursor. J. Org. Chem..

[33] Voinov M.A., Polienko J.F., Schanding T., Bobko A.A., Khramtsov V.V., Gatilov Y.V., Rybalova T.V., Smirnov A.I., Grigor’ev I.A. (2005). Synthesis, structure, and X-band (9.5 GHz) EPR characterization of the new series of pH-sensitive spin probes: N,N-disubstituted 4-amino-2,2,5,5-tetramethyl-3-imidazoline 1-oxyls. J. Org. Chem..

[34] Kirilyuk I.A., Polienko Y.F., Krumkacheva O.A., Strizhakov R.K., Gatilov Y.V., Grigor’ev I.A., Bagryanskaya E.G. (2012). Synthesis of 2,5-bis(spirocyclohexane)-substituted Nitroxides of Pyrroline and Pyrrolidine series, including Thiol-specific spin label: An analogue of MTSSL with long relaxation time. J. Org. Chem..

[35] Polienko Y.F., Vinogradova V.I., Sagdullaev S.S., Abdullaev N.D., Gatilov Y.V., Grigor’ev I.A. (2013). First spin-labeled cytisine derivatives. Chem. Nat. Compd..

[36] Dobrynin S.A., Usatov M.S., Zhurko I.F., Morozov D.A., Polienko Y.F., Glazachev Y.I., Parkhomenko D.A., Tyumentsev M.A., Gatilov Y.V., Chernyak E.I. (2021). A Simple Method of Synthesis of 3-Carboxy-2,2,5,5-Tetraethylpyrrolidine-1-oxyl and Preparation of Reduction-Resistant Spin Labels and Probes of Pyrrolidine Series. Molecules.

[37] Antimonova A.N., Petrenko N.I., Shults E.E., Polienko Y.F., Shakirov M.M., Irtegova I.G., Pokrovskii M.A., Sherman K.M., Grigor’ev I.A., Pokrovskii A.G. (2013). Synthetic transformations of higher triterpenoids. XXX: Synthesis and cytotoxic activity of betulonic acid amides with fragments of nitroxyl radicals. Russ. J. Bioorg. Chem..

[38] Krause L., Herbst-Irmer R., Sheldrick G.M., Stalke D. (2015). Comparison of silver and molybdenum microfocus X-ray sources for single-crystal structure determination. J. Appl. Cryst..

[39] Sheldrick G.M. (2008). A short history of SHELX. Acta Crystallogr. Sect. A.

[40] Sheldrick G.M. (2015). Crystal structure refinement with SHELXL. Acta Crystallogr. Sect. C.

[41] Li C., Yuan C. (1993). Studies on organophosphorus compounds 81. A novel synthetic approach to substituted cyclopentane-1,1-diylbisphosphonates via Pd(0) catalyzed enyne cyclization. Heteroat. Chem..

