# Supplementary material for: 4-Dialkylamino-2,5-dihydroimidazol-1-oxyls with Functional Groups at the Position 2 and at the Exocyclic Nitrogen: The pH-Sensitive Spin Labels
Source: Gels. 2021 Dec 23;8(1):11. doi: 10.3390/gels8010011 (PMC8774874; doi:10.3390/gels8010011)
Supplement: Supplementary file 1 [file gels-08-00011-s001.zip › gels-1514739-supplementary.pdf]

gels

Supplementary Materials

4-Dialkylamino-2,5-dihydroimidazol-1-oxyls with Functional Groups at the Position 2 and at the Exocyclic Nitrogen: The pH-Sensitive Spin Labels

Dmitrii G. Trofimov [1], Yuri I. Glazachev [2], Artem A. Gorodetsky [1], Denis A. Komarov [1], Tatyana V. Rybalova [1] and Igor A. Kirilyuk [1,*]

Table of Contents

1. IR spectra **S4**

1.1. 2-(4-(Azidomethyl)phenyl)-2,5,5-triethyl-4-pyrrolidino-2,5-dihydro-1H-imidazol-1-oxyl (**5**) (KBr) S4

1.2. Tetraisopropyl but-3-yne-1,1-diyldiphosphonate (**6**) (neat) S4

1.3. 2-(4-((4-(2,2-Bis(diisopropoxyphosphoryl)ethyl)-1H-1,2,3-triazol-1-yl)methyl)phenyl)-2,5,5-triethyl-4-pyrrolidino-2,5-dihydro-1H-imidazol-1-oxyl(**7**) (neat).......... S4

1.4. 2,5,5-Triethyl-2-(4-formylphenyl)-4-pyrrolidino-2,5-dihydro-1H-imidazol-1-oxyl (**9**) (KBr) S5

1.5. 2,5,5-Triethyl-2-(4-ethynylphenyl)-4-pyrrolidino-2,5-dihydro-1H-imidazol-1-oxyl (**11**) (KBr).......... S5

1.6. 2-(4-Carboxyphenyl)-2,5,5-triethyl-4-pyrrolidino-2,5-dihydro-1H-imidazol-1-oxyl (**12**)(KBr) S5

1.7. 2-(4-(Ethoxycarbonyl)phenyl)-2,5,5-triethyl-4-pyrrolidino-2,5-dihydro-1H-imidazol-1-oxyl (**14**) (KBr) S6

1.8. 2-(4-((2,5-Dioxopyrrolidinooxy)carbonyl)phenyl)-2,5,5-triethyl-4-pyrrolidino-2,5-dihydro-1H-imidazol-1-oxyl (**15**) (KBr).......... S6

1.9. 2-(4-((3-Carboxypropanoyloxy)methyl)phenyl)-2,5,5-triethyl-4-pyrrolidino-2,5-dihydro-1H-imidazol-1-oxyl (**16**) (KBr) S6

1.10. 2-Allyl-2-ethyl-5,5-dimethyl-4-(pyrrolidino)-2,5-dihydroimidazol-1-oxyl (**18a**) (KBr).......... S7

1.11. 2-Ethyl-5,5-dimethyl-2-(pent-4-enyl)-4-(pyrrolidino)-2,5-dihydroimidazol-1-oxyl (**18b**) (KBr)...... S7

1.12. 2-Ethyl-2-(3-hydroxypropyl)-5,5-dimethyl-4-(pyrrolidino)-2,5-dihydroimidazol-1-oxyl (**19a**) (KBr)S8

1.13. 2-Ethyl-2-(5-hydroxypentyl)-5,5-dimethyl-4-(pyrrolidino)-2,5-dihydroimidazol-1-oxyl (**19b**) (KBr)S8

1.14. 2-(3-(1H-Imidazole-1-carbonyloxy)propyl)-2-ethyl-5,5-dimethyl-4-(pyrrolidino)-2,5-dihydro-1H-imidazol-1-oxyl (**20a**) (KBr) S8

1.15. 2-(5-(1H-Imidazole-1-carbonyloxy)pentyl)-2-ethyl-5,5-dimethyl-4-(pyrrolidino)-2,5-dihydro-1H-imidazol-1-oxyl (**20b**) (KBr).......... S9

1.16. 2-(3-(3-(Diethylamino)propylcarbamoyloxy)propyl)-2-ethyl-5,5-dimethyl-4-(pyrrolidino)-2,5-dihydro-1H-imidazol-1-oxyl (**21**) (KBr).......... S9

1.17. 2-(3-Carboxypropyl)-2-ethyl-5,5-dimethyl-4-(pyrrolidino)-2,5-dihydro-1H-imidazol-1-oxyl (**22**) (neat) S9

1.18. 2-(2-(1,3-Dioxolan-2-yl)ethyl)-2-ethyl-5,5-dimethyl-4-(pyrrolidin-1-yl)-2,5-dihydroimidazol-1-oxyl (**23**) (neat) S10

1.19. 2-ethyl-5,5-dimethyl-2-(3-oxopropyl)-4-(pyrrolidin-1-yl)-2,5-dihydro-1H-imidazol-1-oxyl (**24**) (neat) S10

1.20. 2-(2-carboxyethyl)-2-ethyl-5,5-dimethyl-4-(pyrrolidin-1-yl)-2,5-dihydro-1H-imidazol-1-oxyl (**25**) (neat) S10

1.21. 1-(4-(1,3-dioxolan-2-yl)phenyl)-N-methylmethanamine (**26**) (neat) S11

1.22. 5-((4-(1,3-dioxolan-2-yl)benzyl)(methyl)amino)-4,4-dimethyl-2-(pyridin-4-yl)-4H-imidazole 3-oxide (**30**) (KBr) S11

1.23. 4-((4-(1,3-dioxolan-2-yl)benzyl)(methyl)amino)-2-ethyl-5,5-dimethyl-2-(pyridin-4-yl)-2,5-dihydro-1H-imidazol-1-oxyl (**31**) (neat) S11

1.24. 2-ethyl-4-((4-formylbenzyl)(methyl)amino)-5,5-dimethyl-2-(pyridin-4-yl)-2,5-dihydro-1H-imidazol-1-oxyl (**32**) (KBr) S12

1.25. 4-((4-carboxybenzyl)(methyl)amino)-2-ethyl-5,5-dimethyl-2-(pyridin-4-yl)-2,5-dihydro-1H-imidazol-1-oxyl (**33**) (KBr) S12

1.26. 2-ethyl-4-((4-(hydroxymethyl)benzyl)(methyl)amino)-5,5-dimethyl-2-(pyridin-4-yl)-2,5-dihydro-1H-imidazol-1-oxyl (**34**) (KBr) S12

1.27. 4-((4-(((2,5-dioxopyrrolidin-1-yl)oxy)carbonyl)benzyl)(methyl)amino)-2-ethyl-5,5-dimethyl-2-(pyridin-4-yl)-2,5-dihydro-1H-imidazol-1-oxyl (**35**) (KBr) S13

2. ^{1}H NMR spectra. S13

2.1. Tetraisopropyl but-3-yne-1,1-diyldiphosphonate(**6**)(400 MHz, $CDCl_3$) S13

2.2. 2-(4-((4-(2,2-Bis(diisopropoxyphosphoryl)ethyl)-1H-1,2,3-triazol-1-yl)methyl)phenyl)-2,5,5-triethyl-4-pyrrolidino-2,5-dihydro-1H-imidazol-1-oxyl (**7**)(300 MHz; CD_3OD–$CDCl_3$, reduced with Zn in ND_4Cl/D_2O) S14

2.3. 2-(4-Carboxyphenyl)-2,5,5-triethyl-4-pyrrolidino-2,5-dihydro-1H-imidazol-1-oxyl (**12**) (400 MHz; CD_3OD–$CDCl_3$, reduced with PhSH) S14

2.4. 2-(4-(Ethoxycarbonyl)phenyl)-2,5,5-triethyl-4-pyrrolidino-2,5-dihydro-1H-imidazol-1-oxyl (**14**) (300 MHz; $CDCl_3$–CD_3OD, reduced with Zn/CF_3COOH in CD_3OD, 65 °C) S15

2.5. 2-(4-((3-Carboxypropanoyloxy)methyl)phenyl)-2,5,5-triethyl-4-pyrrolidino-2,5-dihydro-1H-imidazol-1-oxyl (**16**) (300 MHz; $CDCl_3$ – CD_3OD, reduced with Zn/CF_3COOH in CD_3OD, 65 °C) S15

2.6. 2-Allyl-2-ethyl-5,5-dimethyl-4-(pyrrolidino)-2,5-dihydroimidazol-1-oxyl (**18a**) (400 MHz; CD_3OD, reduced with N_2D_4) S16

2.7. 2-Ethyl-5,5-dimethyl-2-(pent-4-enyl)-4-(pyrrolidino)-2,5-dihydroimidazol-1-oxyl (**18b**) (300 MHz; $CDCl_3$ – CD_3OD, reduced with Zn/CF_3COOH in CD_3OD, 65 °C) S16

2.8. 2-Ethyl-2-(3-hydroxypropyl)-5,5-dimethyl-4-(pyrrolidino)-2,5-dihydroimidazol-1-oxyl (**19a**) (400 MHz; CD_3OD, reduced with N_2D_4) S17

2.9. 2-Ethyl-2-(5-hydroxypentyl)-5,5-dimethyl-4-(pyrrolidino)-2,5-dihydroimidazol-1-oxyl (**19b**) (300 MHz; $CDCl_3$–CD_3OD, reduced with Zn/CF_3COOH in CD_3OD, 65 °C) S17

2.10. 2-(3-(3-(Diethylamino)propylcarbamoyloxy)propyl)-2-ethyl-5,5-dimethyl-4-(pyrrolidino)-2,5-dihydro-1H-imidazol-1-oxyl (**21**) (300 MHz; $CDCl_3$ – CD_3OD, reduced with Zn/CF_3COOH in CD_3OD, 65 °C) S18

2.11. 2-(2-carboxyethyl)-2-ethyl-5,5-dimethyl-4-(pyrrolidin-1-yl)-2,5-dihydro-1H-imidazol-1-oxyl (**25**) (300 MHz; $CDCl_3$–CD_3OD, reduced with Zn/CF_3COOH in CD_3OD, 65 °C) S18

2.12. 1-(4-(1,3-dioxolan-2-yl)phenyl)-N-methylmethanamine (**26**) (300 MHz; $CDCl_3$) S19

2.13. 5-((4-(1,3-dioxolan-2-yl)benzyl)(methyl)amino)-4,4-dimethyl-2-(pyridin-4-yl)-4H-imidazole 3-oxide (**30**) (400 MHz; $CDCl_3$) S19

2.14. 2-Ethyl-4-((4-(hydroxymethyl)benzyl)(methyl)amino)-5,5-dimethyl-2-(pyridin-4-yl)-2,5-dihydroimidazol-1-oxyl (**34**) (400 MHz; $CDCl_3$, CD_3OD, reduced with Zn/CF_3COOH in CD_3OD) S20

3. ^{13}C NMR spectra S20

3.1. Tetraisopropyl but-3-yne-1,1-diyldiphosphonate(**6**)(75 MHz, $CDCl_3$)........ S20

3.2. 1-(4-(1,3-dioxolan-2-yl)phenyl)-N-methylmethanamine (**26**) (75 MHz; $CDCl_3$) S21

3.3. 5-((4-(1,3-dioxolan-2-yl)benzyl)(methyl)amino)-4,4-dimethyl-2-(pyridin-4-yl)-4H-imidazole 3-oxide (**30**) (75 MHz; $CDCl_3$) S21

4. Titration data. S22

4.1. 2-(4-(Azidomethyl)phenyl)-2,5,5-triethyl-4-pyrrolidino-2,5-dihydro-1H-imidazol-1-oxyl (**5**) S22

4.2. 2-(4-((4-(2,2-Bis(diisopropoxyphosphoryl)ethyl)-1H-1,2,3-triazol-1-yl)methyl)phenyl)-2,5,5-triethyl-4-pyrrolidino-2,5-dihydro-1H-imidazol-1-oxyl (**7**)........ S22

4.3. 2,5,5-Triethyl-2-(4-ethynylphenyl)-4-pyrrolidino-2,5-dihydro-1H-imidazol-1-oxyl (**11**) S23

4.4 2-(4-Carboxyphenyl)-2,5,5-triethyl-4-pyrrolidino-2,5-dihydro-1H-imidazol-1-oxyl (**12**) S23

4.5. 2-(4-((2,5-Dioxopyrrolidinooxy)carbonyl)phenyl)-2,5,5-triethyl-4-pyrrolidino-2,5-dihydro-1H-imidazol-1-oxyl (**15**) S24

4.6. 2-(4-((3-Carboxypropanoyloxy)methyl)phenyl)-2,5,5-triethyl-4-pyrrolidino-2,5-dihydro-1H-imidazol-1-oxyl (**16**) S24

4.7. 2-Allyl-2-ethyl-5,5-dimethyl-4-(pyrrolidino)-2,5-dihydroimidazol-1-oxyl (**18a**) S25

4.8. 2-Ethyl-5,5-dimethyl-2-(pent-4-enyl)-4-(pyrrolidino)-2,5-dihydroimidazol-1-oxyl (**18b**) S25

4.9. 2-Ethyl-2-(3-hydroxypropyl)-5,5-dimethyl-4-(pyrrolidino)-2,5-dihydroimidazol-1-oxyl (**19a**)........ S26

4.10. 2-Ethyl-2-(5-hydroxypentyl)-5,5-dimethyl-4-(pyrrolidino)-2,5-dihydroimidazol-1-oxyl (**19b**) S26

4.11. 2-(3-(1H-Imidazole-1-carbonyloxy)propyl)-2-ethyl-5,5-dimethyl-4-(pyrrolidino)-2,5-dihydro-1H-imidazol-1-oxyl (**20a**)........ S27

4.12. 2-(3-(3-(Diethylamino)propylcarbamoyloxy)propyl)-2-ethyl-5,5-dimethyl-4-(pyrrolidino)-2,5-dihydro-1H-imidazol-1-oxyl (**21**) S27

4.13. 2-(3-Carboxypropyl)-2-ethyl-5,5-dimethyl-4-(pyrrolidino)-2,5-dihydro-1H-imidazol-1-oxyl (**22**)S28

4.15. 2-(2-carboxyethyl)-2-ethyl-5,5-dimethyl-4-(pyrrolidin-1-yl)-2,5-dihydro-1H-imidazol-1-oxyl (**25**)S29

4.16. 2-ethyl-4-((4-formylbenzyl)(methyl)amino)-5,5-dimethyl-2-(pyridin-4-yl)-2,5-dihydro-1H-imidazol-1-oxyl (32) S29

4.17. 4-((4-carboxybenzyl)(methyl)amino)-2-ethyl-5,5-dimethyl-2-(pyridin-4-yl)-2,5-dihydro-1H-imidazol-1-oxyl (**33**) S29

5. X-Ray data for nitroxide 11. .... S30

1. IR Spectra

*1.1. 2-(4-(Azidomethyl)phenyl)-2,5,5-triethyl-4-pyrrolidino-2,5-dihydro-1H-imidazol-1-oxyl (**5**) (KBr)*

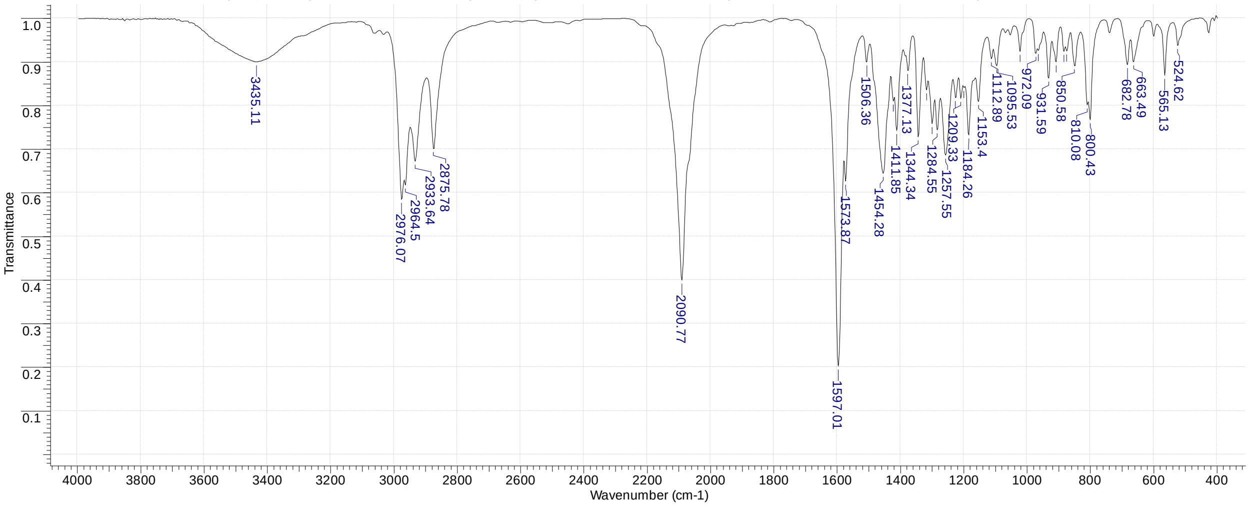

*1.2. Tetraisopropyl but-3-yne-1,1-diyldiphosphonate (**6**) (neat)*

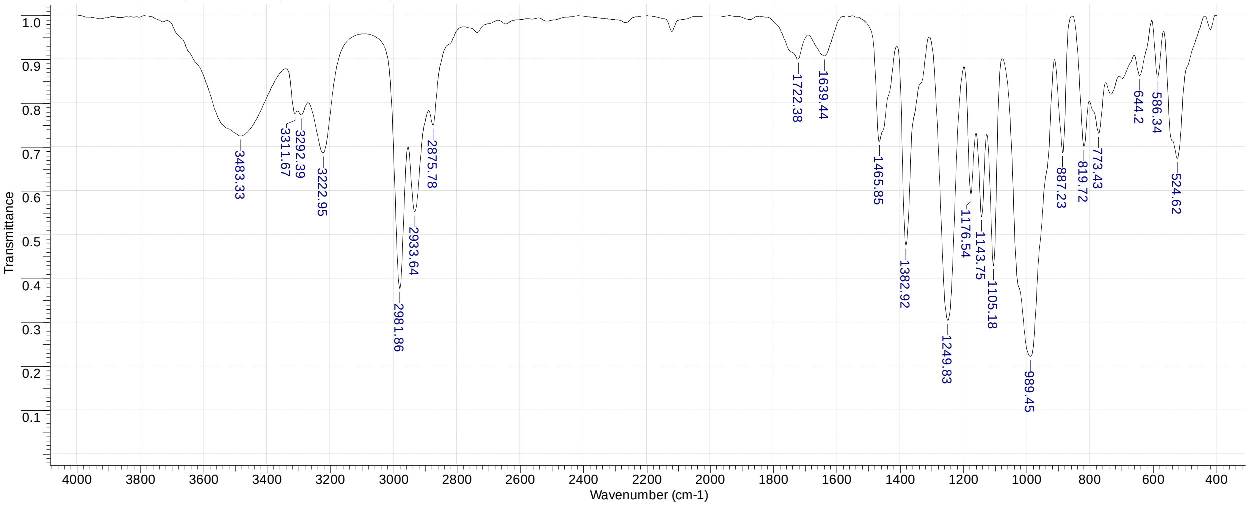

*1.3. 2-(4-((4-(2,2-Bis(diisopropoxyphosphoryl)ethyl)-1H-1,2,3-triazol-1-yl)methyl)phenyl)-2,5,5-triethyl-4-pyrrolidino-2,5-dihydro-1H-imidazol-1-oxyl(**7**) (neat)*

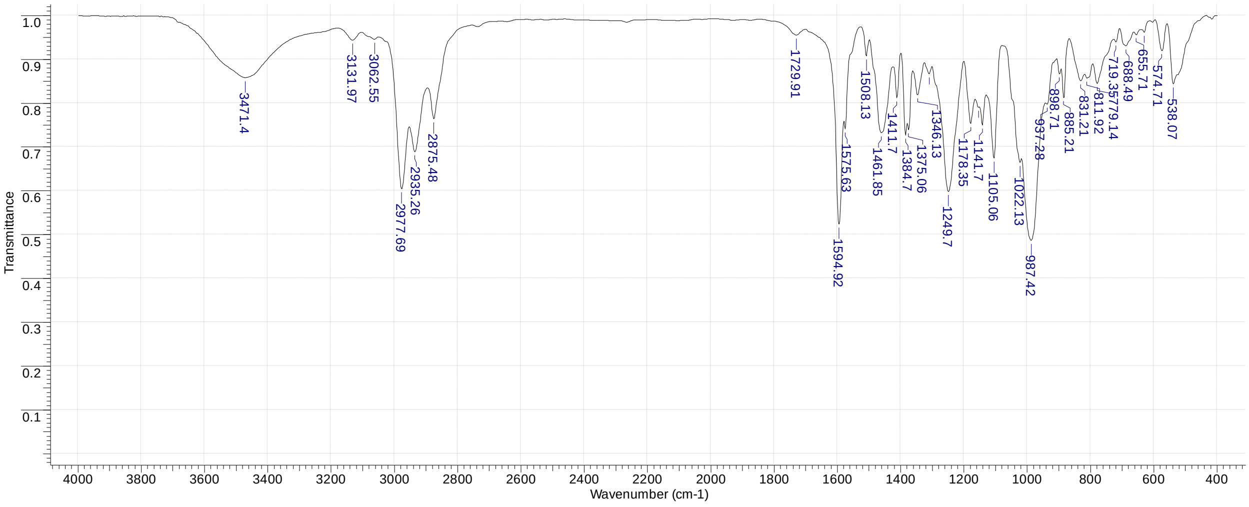

*1.4. 2,5,5-Triethyl-2-(4-formylphenyl)-4-pyrrolidino-2,5-dihydro-1H-imidazol-1-oxyl (**9**) (KBr)*

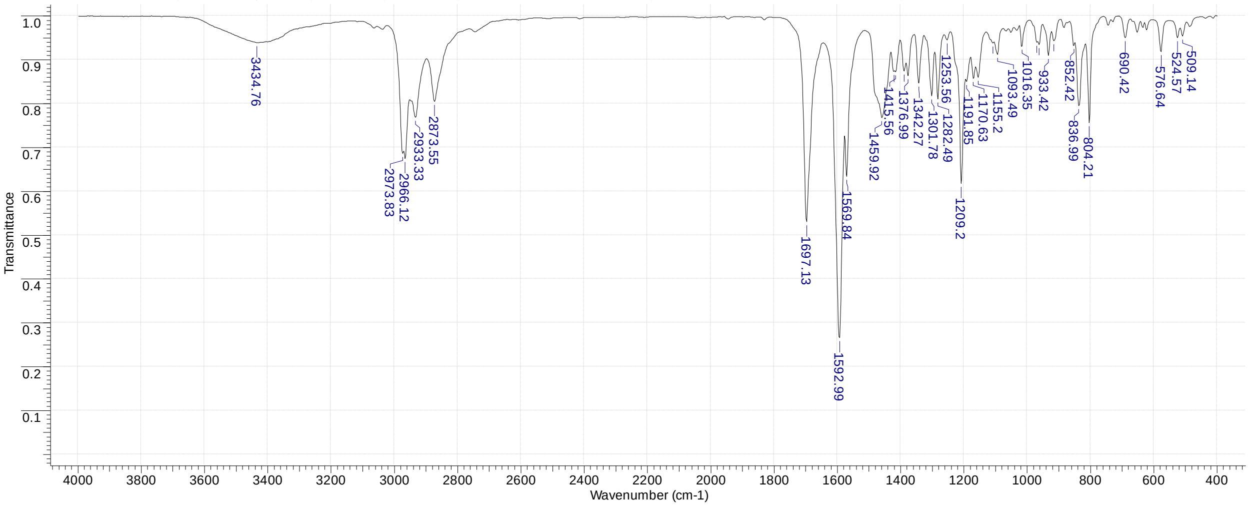

*1.5. 2,5,5-Triethyl-2-(4-ethynylphenyl)-4-pyrrolidino-2,5-dihydro-1H-imidazol-1-oxyl (**11**) (KBr)*

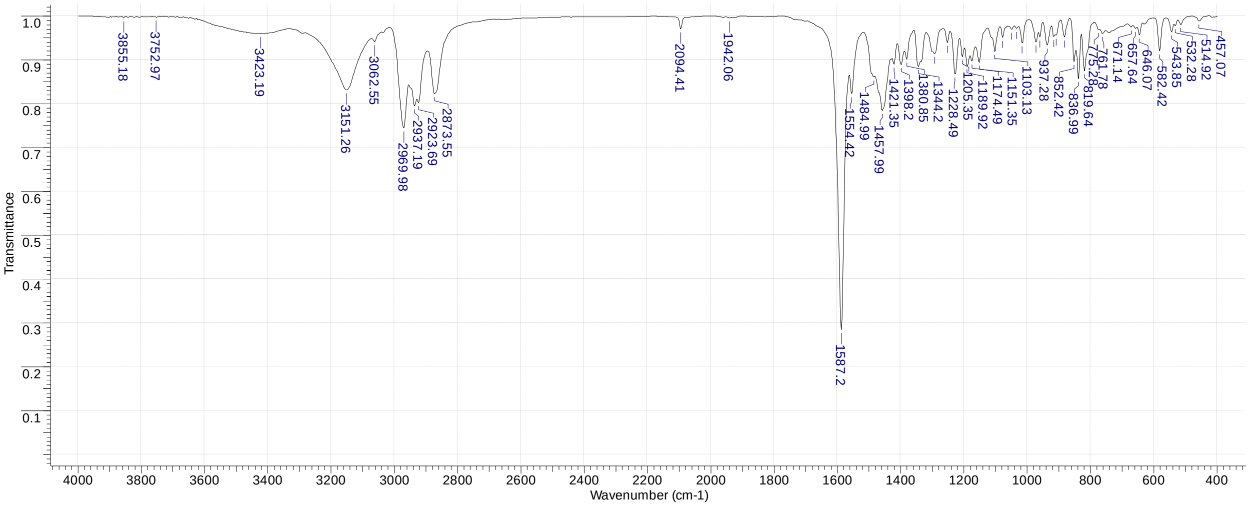

*1.6. 2-(4-Carboxyphenyl)-2,5,5-triethyl-4-pyrrolidino-2,5-dihydro-1H-imidazol-1-oxyl (**12**)(KBr)*

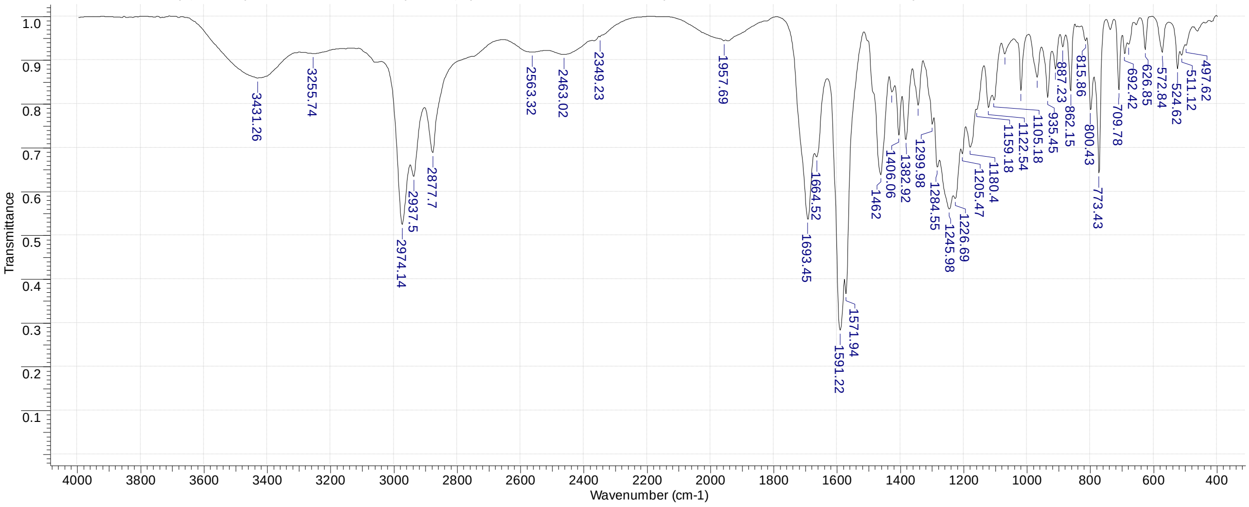

*1.7. 2-(4-(Ethoxycarbonyl)phenyl)-2,5,5-triethyl-4-pyrrolidino-2,5-dihydro-1H-imidazol-1-oxyl (**14**) (KBr)*

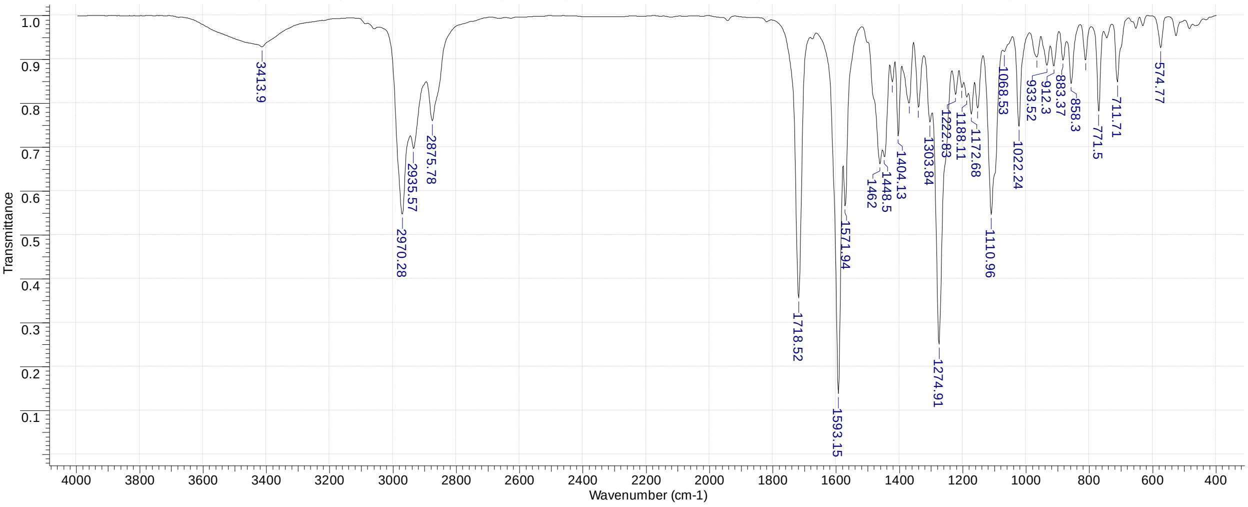

*1.8. 2-(4-((2,5-Dioxopyrrolidinooxy)carbonyl)phenyl)-2,5,5-triethyl-4-pyrrolidino-2,5-dihydro-1H-imidazol-1-oxyl (**15**) (KBr)*

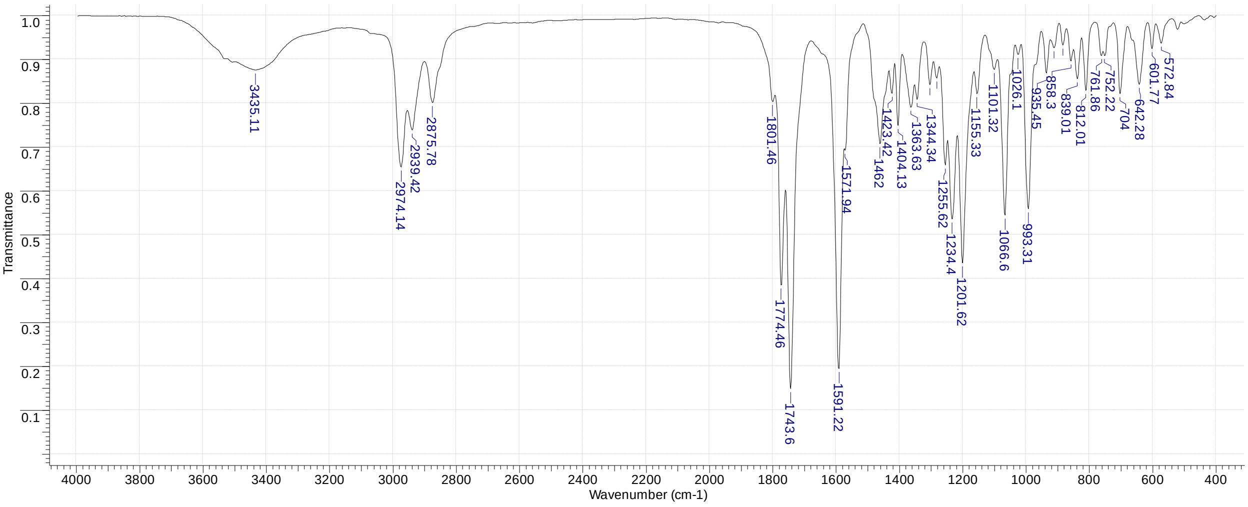

*1.9. 2-(4-((3-Carboxypropanoyloxy)methyl)phenyl)-2,5,5-triethyl-4-pyrrolidino-2,5-dihydro-1H-imidazol-1-oxyl (**16**) (KBr)*

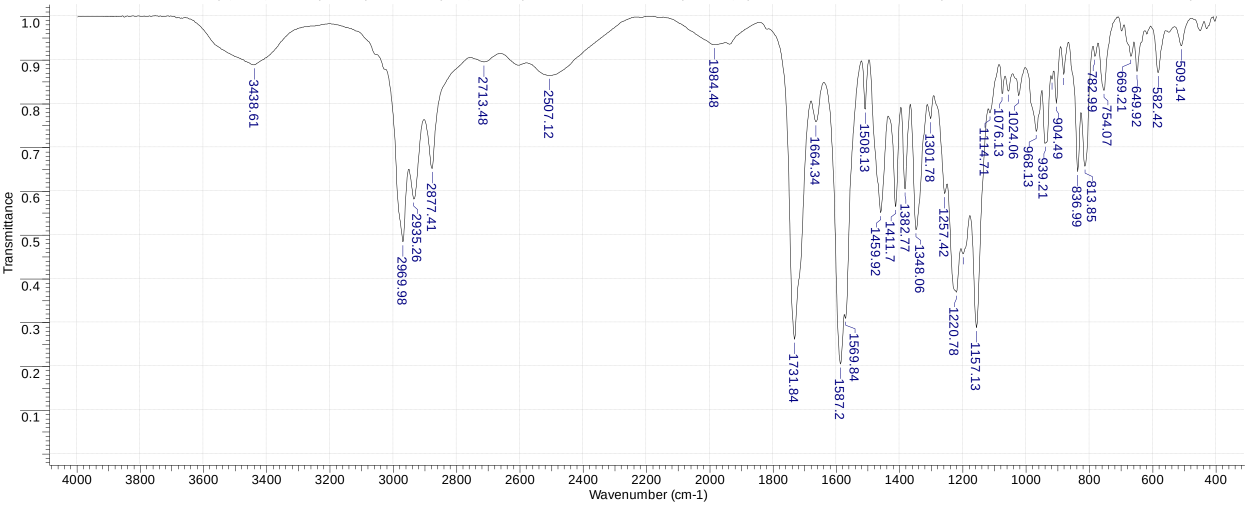

*1.10. 2-Allyl-2-ethyl-5,5-dimethyl-4-(pyrrolidino)-2,5-dihydroimidazol-1-oxyl (**18a**) (KBr)*

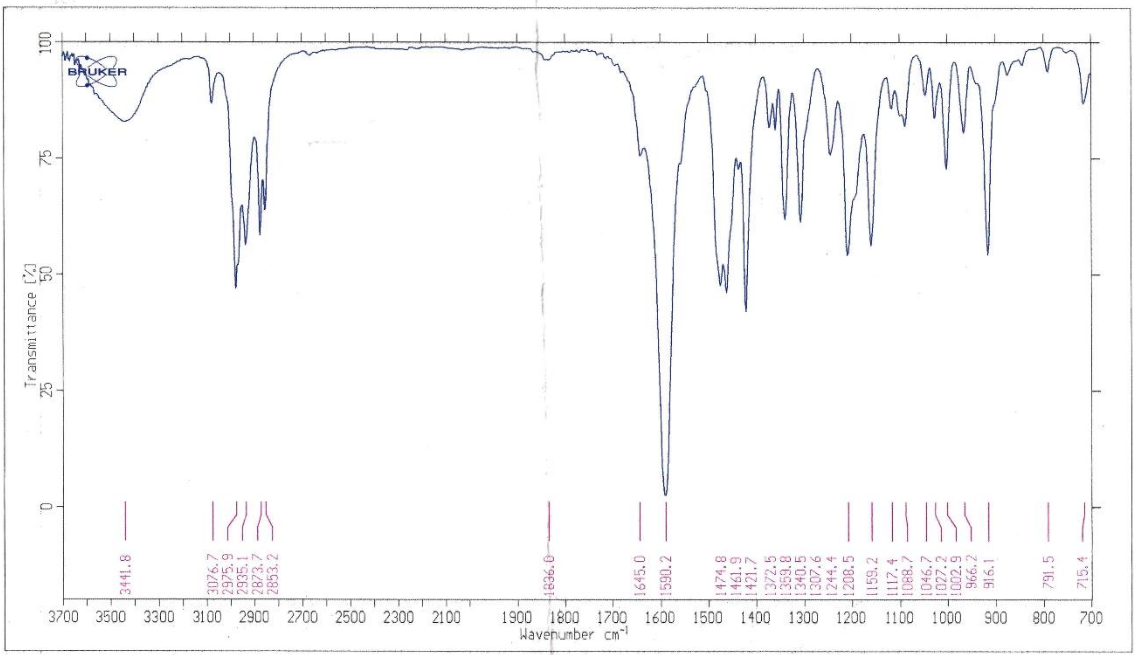

*1.11. 2-Ethyl-5,5-dimethyl-2-(pent-4-enyl)-4-(pyrrolidino)-2,5-dihydroimidazol-1-oxyl (**18b**) (KBr).*

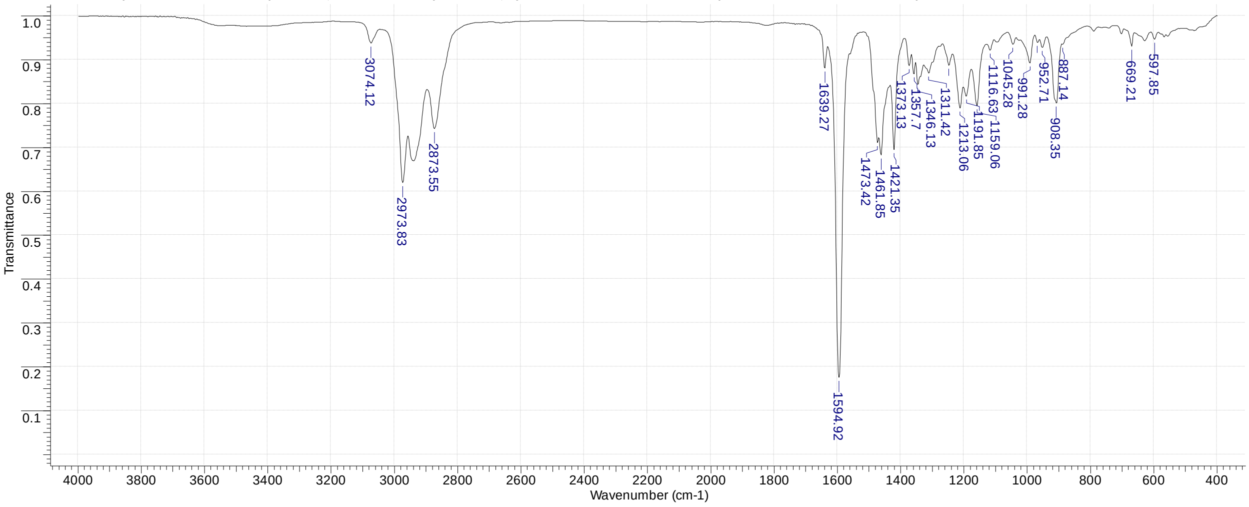

*1.12. 2-Ethyl-2-(3-hydroxypropyl)-5,5-dimethyl-4-(pyrrolidino)-2,5-dihydroimidazol-1-oxyl (**19a**) (KBr)*

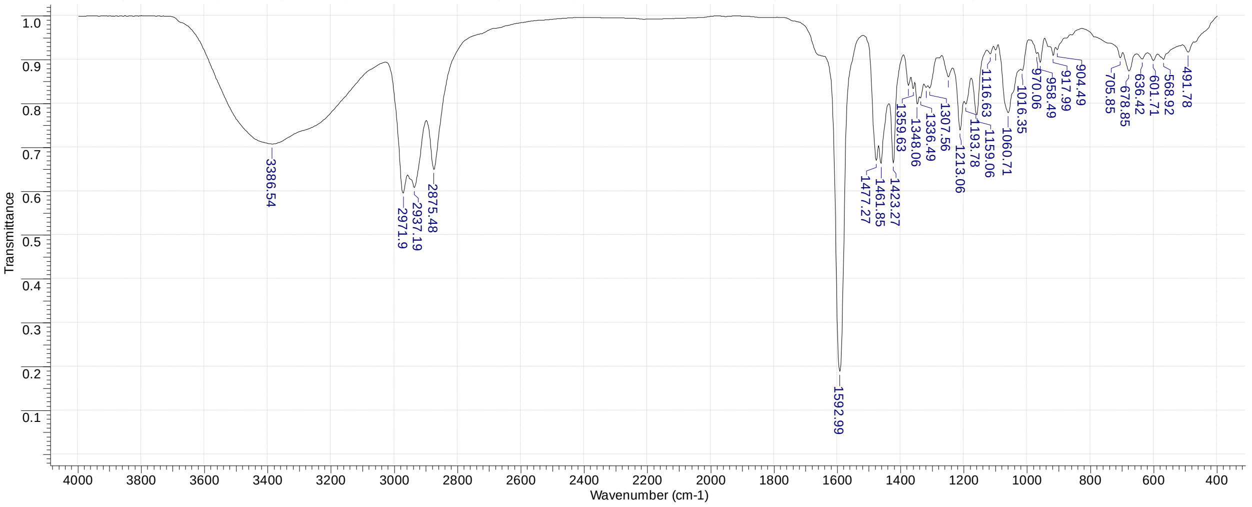

*1.13. 2-Ethyl-2-(5-hydroxypentyl)-5,5-dimethyl-4-(pyrrolidino)-2,5-dihydroimidazol-1-oxyl (**19b**) (KBr)*

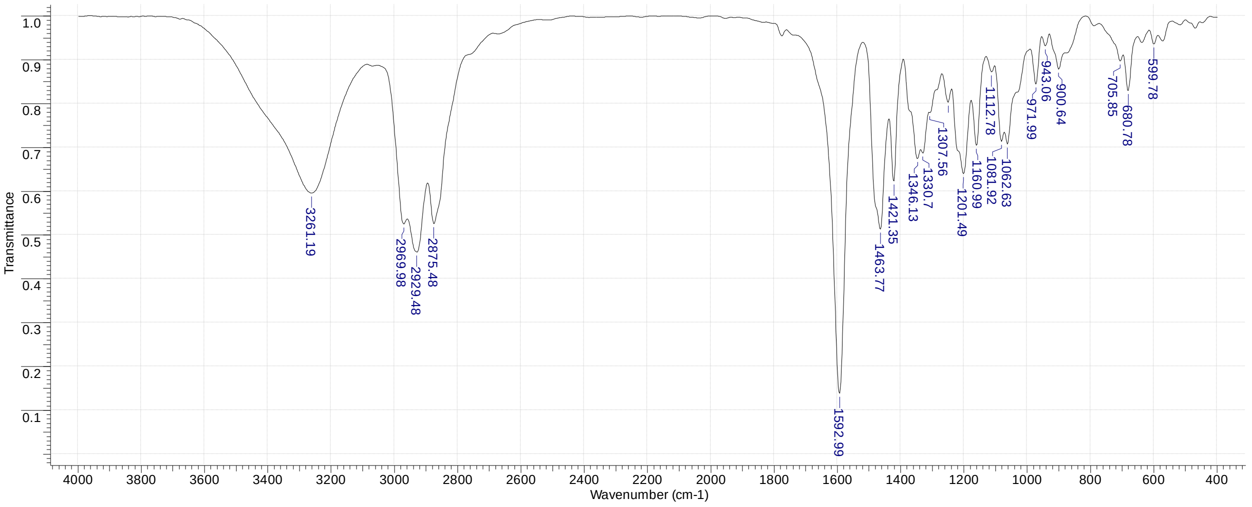

*1.14. 2-(3-(1H-Imidazole-1-carbonyloxy)propyl)-2-ethyl-5,5-dimethyl-4-(pyrrolidino)-2,5-dihydro-1H-imidazol-1-oxyl (**20a**) (KBr)*

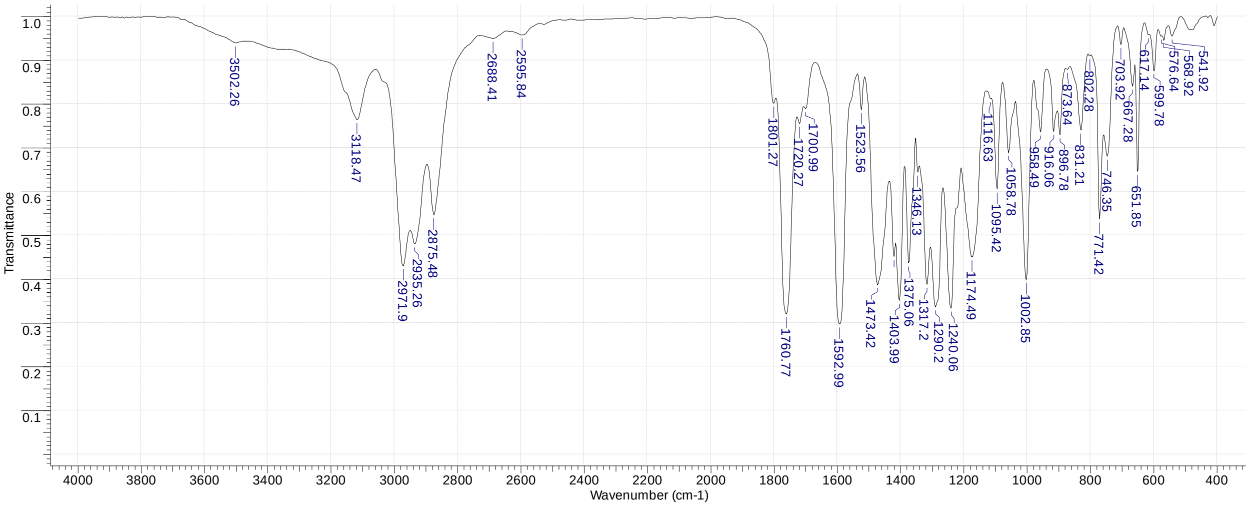

*1.15. 2-(5-(1H-Imidazole-1-carbonyloxy)pentyl)-2-ethyl-5,5-dimethyl-4-(pyrrolidino)-2,5-dihydro-1H-imidazol-1-oxyl (**20b**) (KBr)*

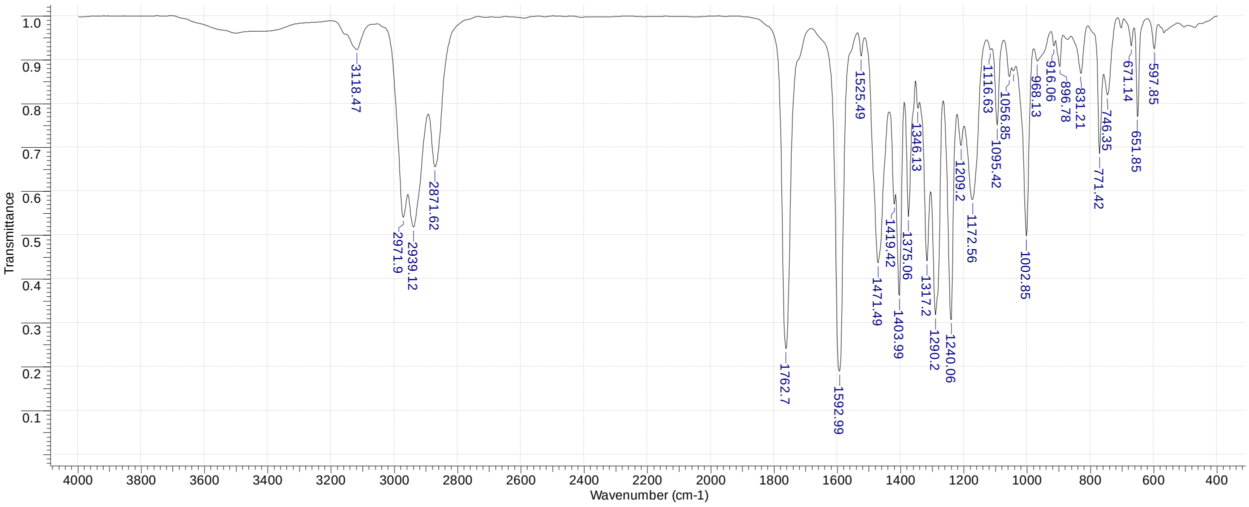

*1.16. 2-(3-(3-(Diethylamino)propylcarbamoyloxy)propyl)-2-ethyl-5,5-dimethyl-4-(pyrrolidino)-2,5-dihydro-1H-imidazol-1-oxyl (**21**) (KBr)*

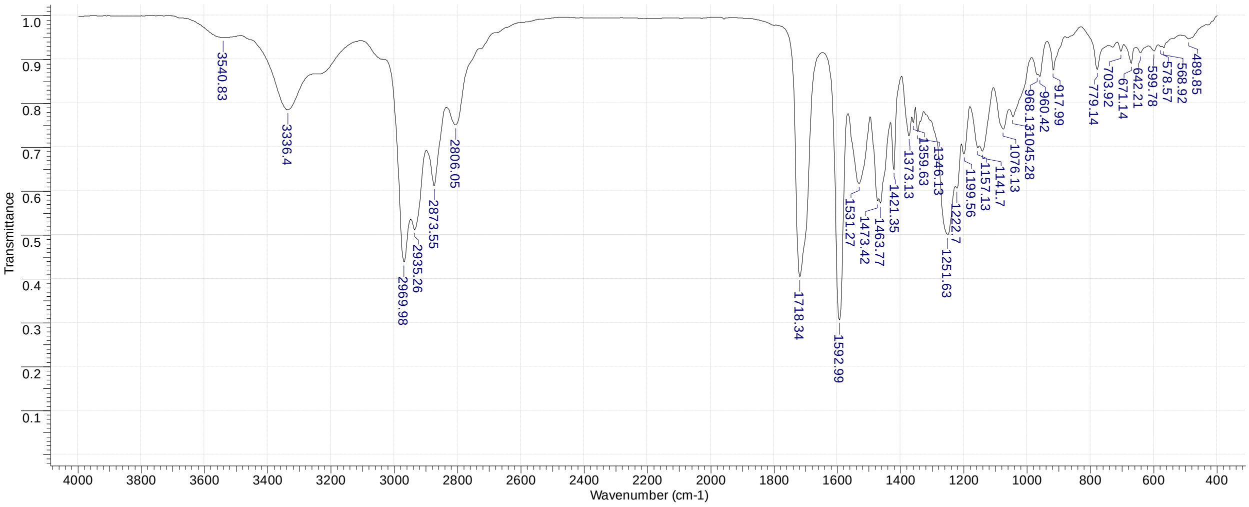

*1.17. 2-(3-Carboxypropyl)-2-ethyl-5,5-dimethyl-4-(pyrrolidino)-2,5-dihydro-1H-imidazol-1-oxyl (**22**) (neat)*

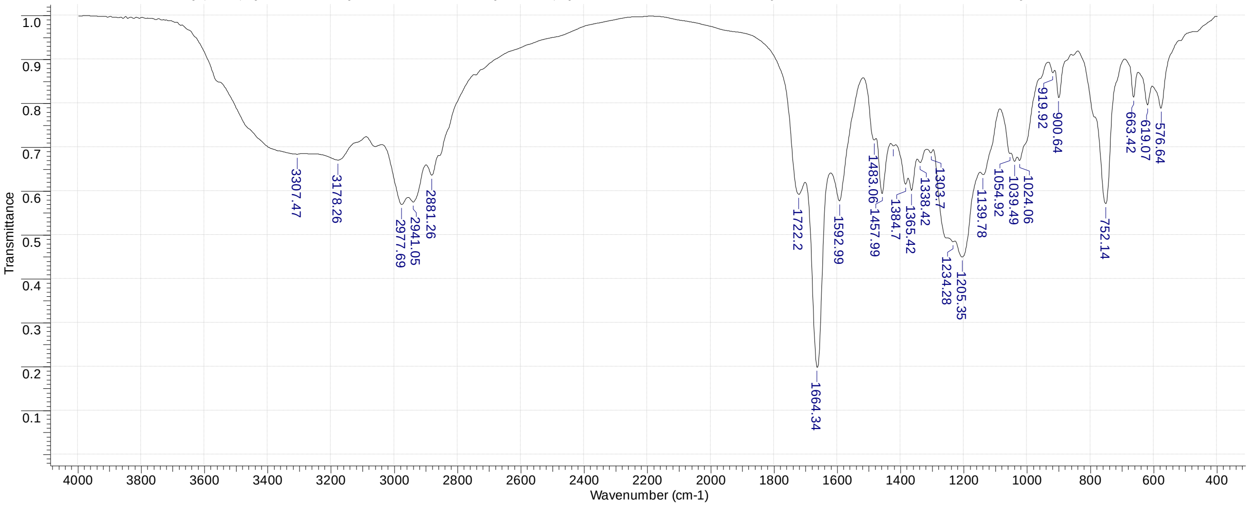

*1.18. 2-(2-(1,3-Dioxolan-2-yl)ethyl)-2-ethyl-5,5-dimethyl-4-(pyrrolidin-1-yl)-2,5-dihydroimidazol-1-oxyl (**23**) (neat)*

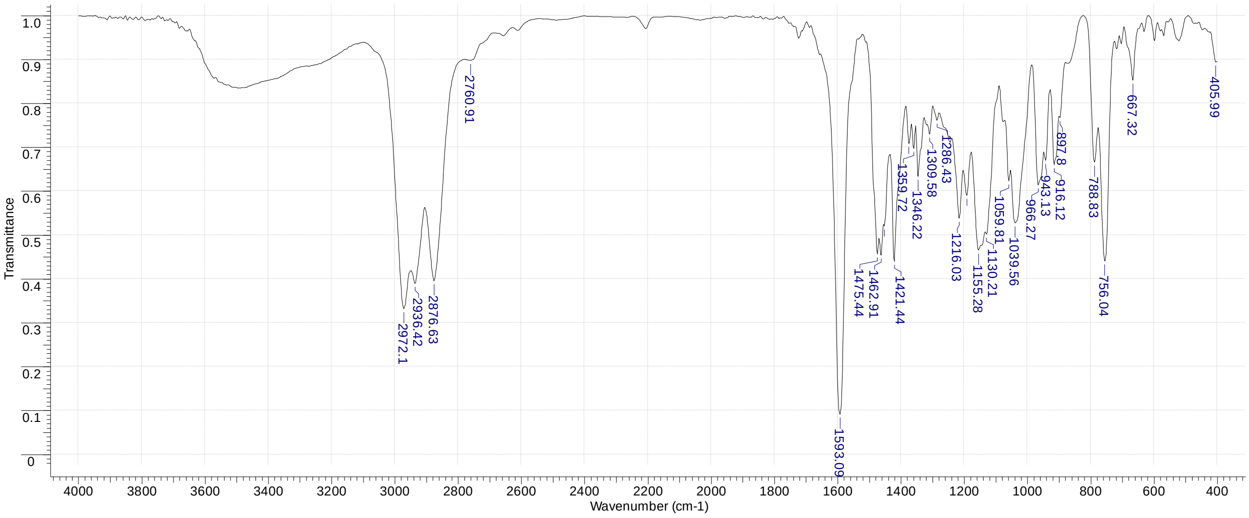

*1.19. 2-ethyl-5,5-dimethyl-2-(3-oxopropyl)-4-(pyrrolidin-1-yl)-2,5-dihydro-1H-imidazol-1-oxyl (**24**) (neat)*

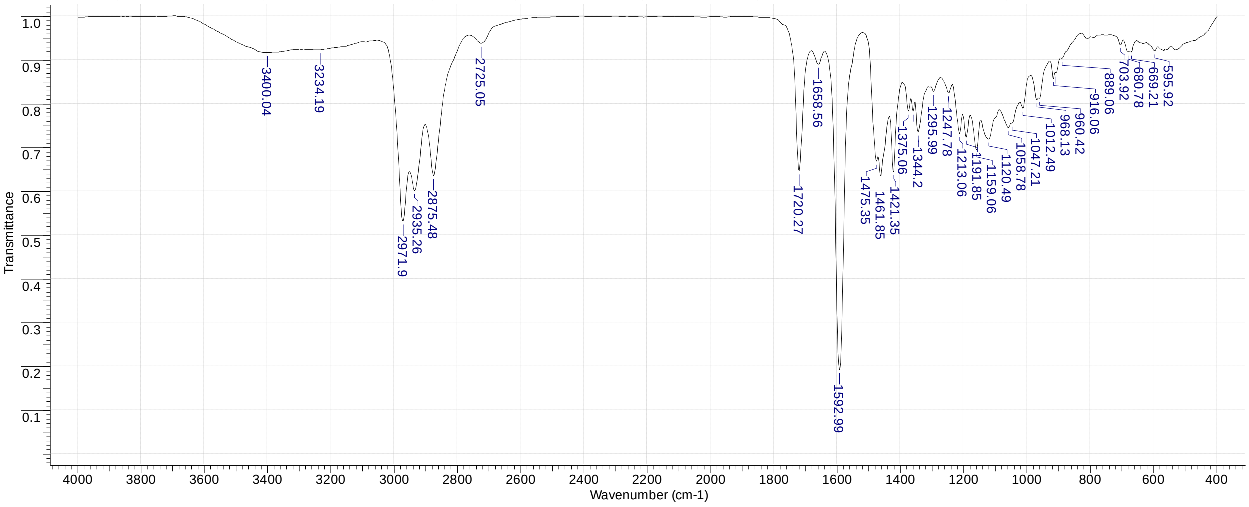

*1.20. 2-(2-carboxyethyl)-2-ethyl-5,5-dimethyl-4-(pyrrolidin-1-yl)-2,5-dihydro-1H-imidazol-1-oxyl (**25**) (neat)*

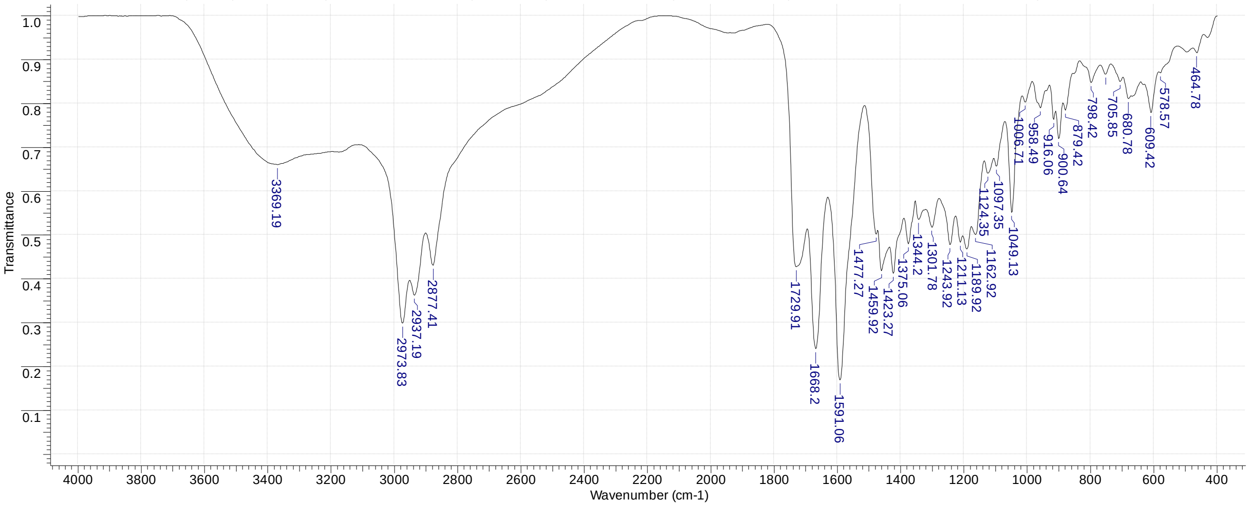

1.21. *1-(4-(1,3-dioxolan-2-yl)phenyl)-N-methylmethanamine (**26**) (neat)*

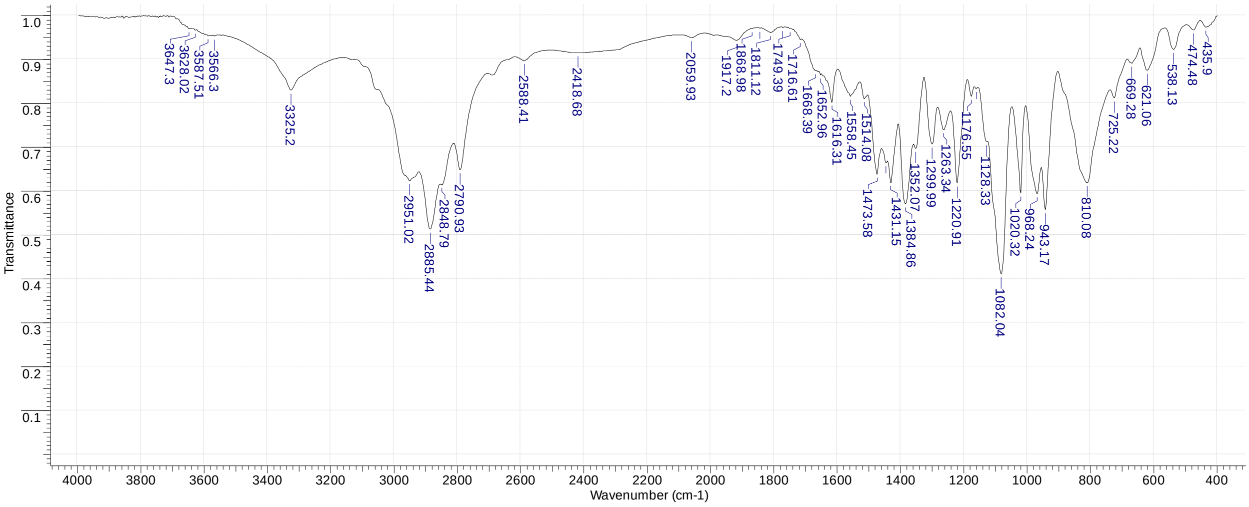

1.22. *5-((4-(1,3-dioxolan-2-yl)benzyl)(methyl)amino)-4,4-dimethyl-2-(pyridin-4-yl)-4H-imidazole 3-oxide (**30**) (KBr)*

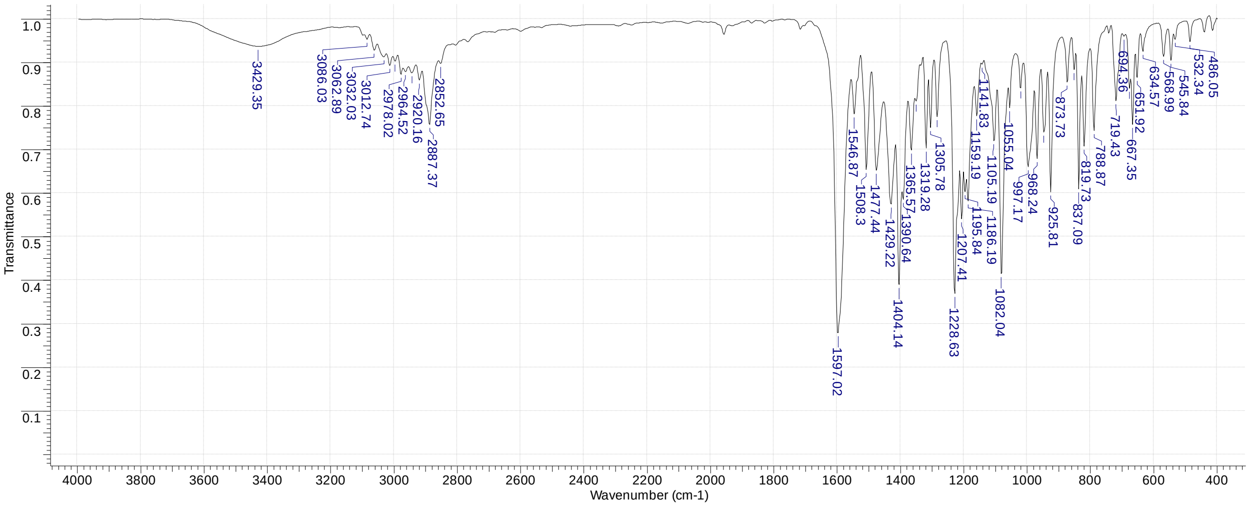

1.23. *4-((4-(1,3-dioxolan-2-yl)benzyl)(methyl)amino)-2-ethyl-5,5-dimethyl-2-(pyridin-4-yl)-2,5-dihydro-1H-imidazol-1-oxyl (**31**) (neat)*

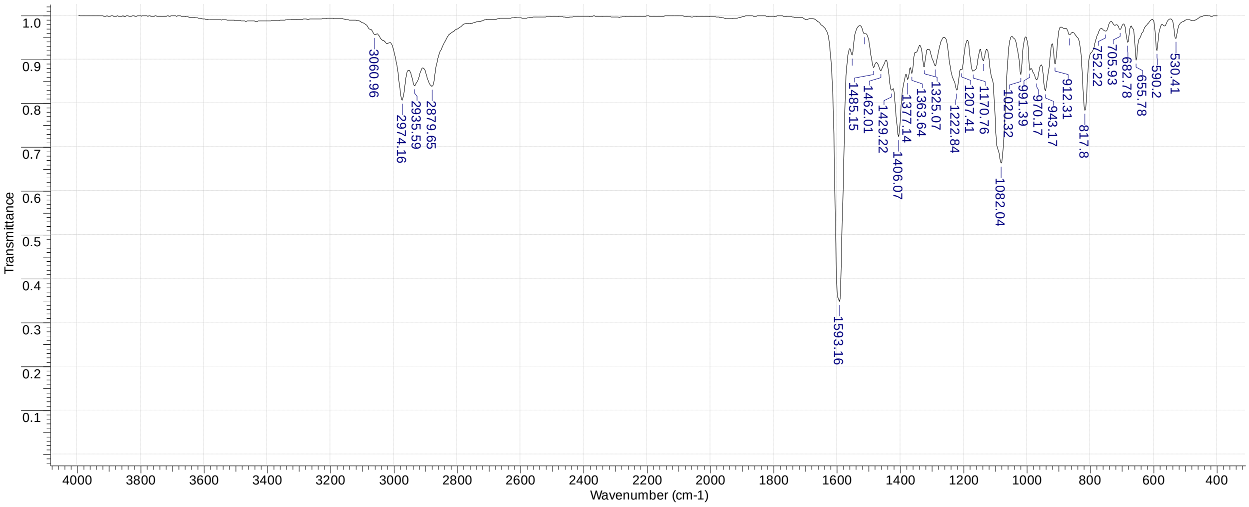

1.24. *2-ethyl-4-((4-formylbenzyl)(methyl)amino)-5,5-dimethyl-2-(pyridin-4-yl)-2,5-dihydro-1H-imidazol-1-oxyl (**32**) (KBr)*

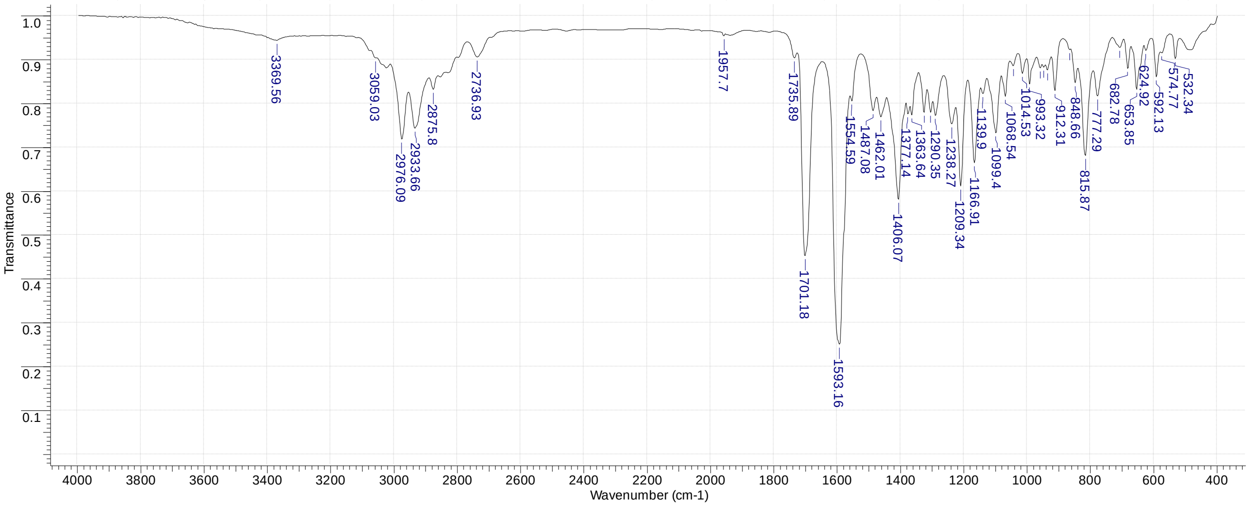

1.25. *4-((4-carboxybenzyl)(methyl)amino)-2-ethyl-5,5-dimethyl-2-(pyridin-4-yl)-2,5-dihydro-1H-imidazol-1-oxyl (**33**) (KBr)*

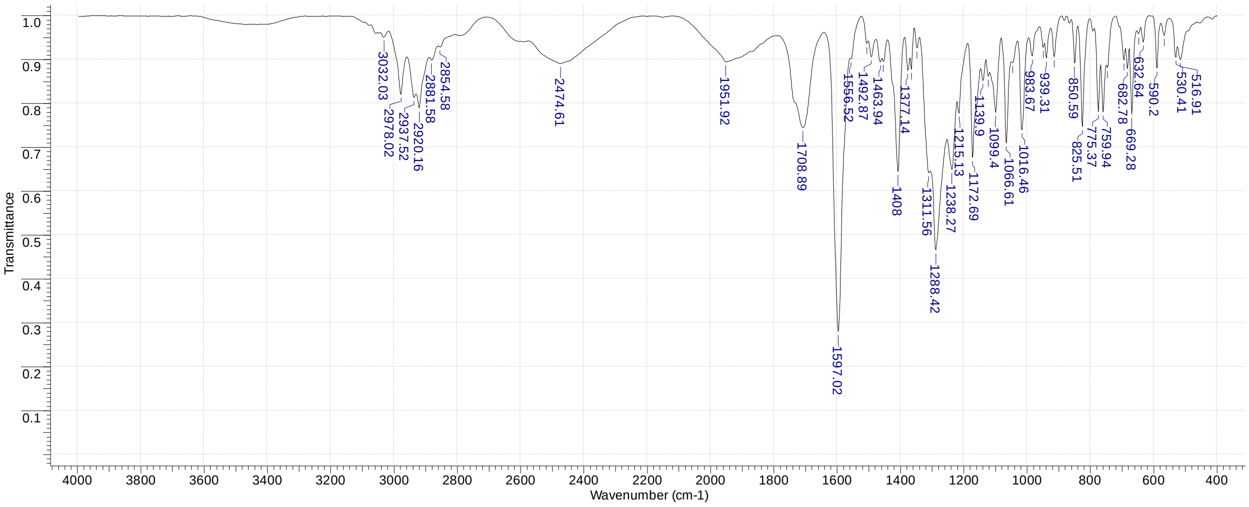

1.26. *2-ethyl-4-((4-(hydroxymethyl)benzyl)(methyl)amino)-5,5-dimethyl-2-(pyridin-4-yl)-2,5-dihydro-1H-imidazol-1-oxyl (**34**) (KBr)*

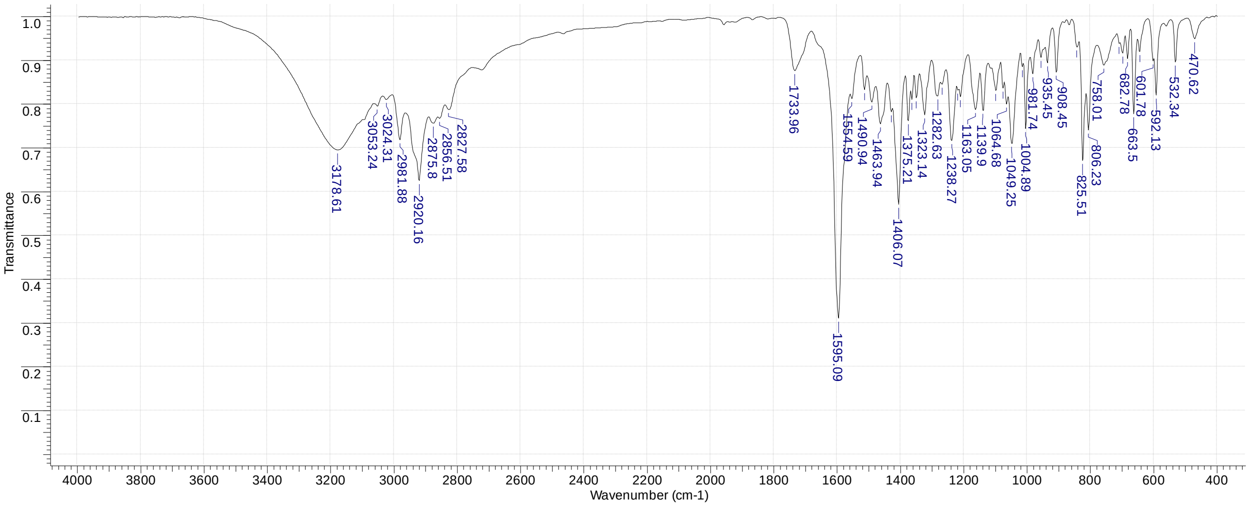

*1.27. 4-((4-(((2,5-dioxopyrrolidin-1-yl)oxy)carbonyl)benzyl)(methyl)amino)-2-ethyl-5,5-dimethyl-2-(pyridin-4-yl)-2,5-dihydro-1H-imidazol-1-oxyl (**35**) (KBr)*

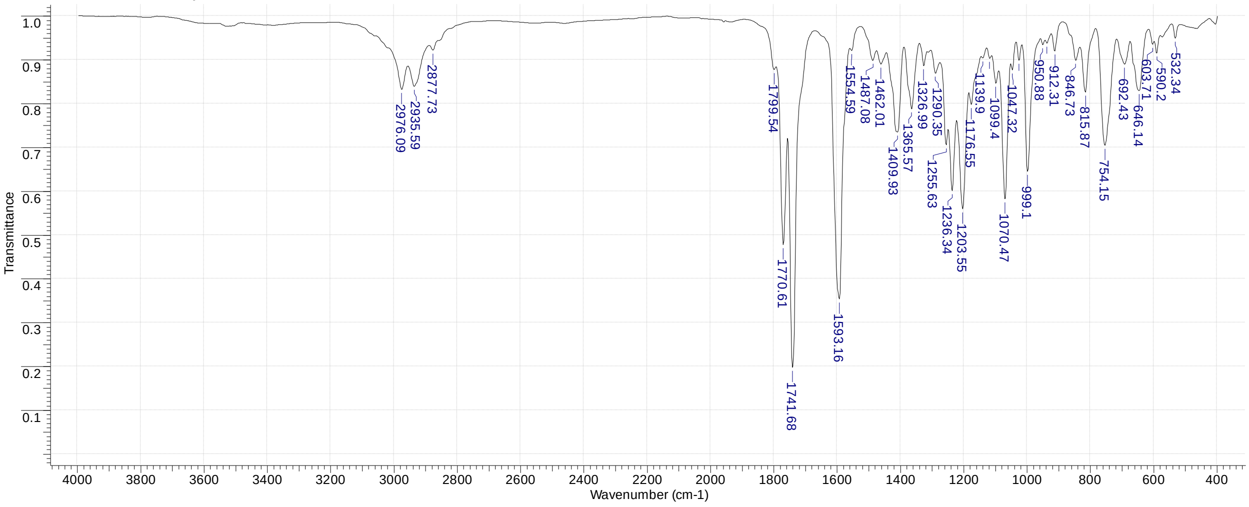

2. ^{1}H NMR Spectra.

*2.1. Tetraisopropyl but-3-yne-1,1-diyldiphosphonate(**6**)(400 MHz, $CDCl_3$)*

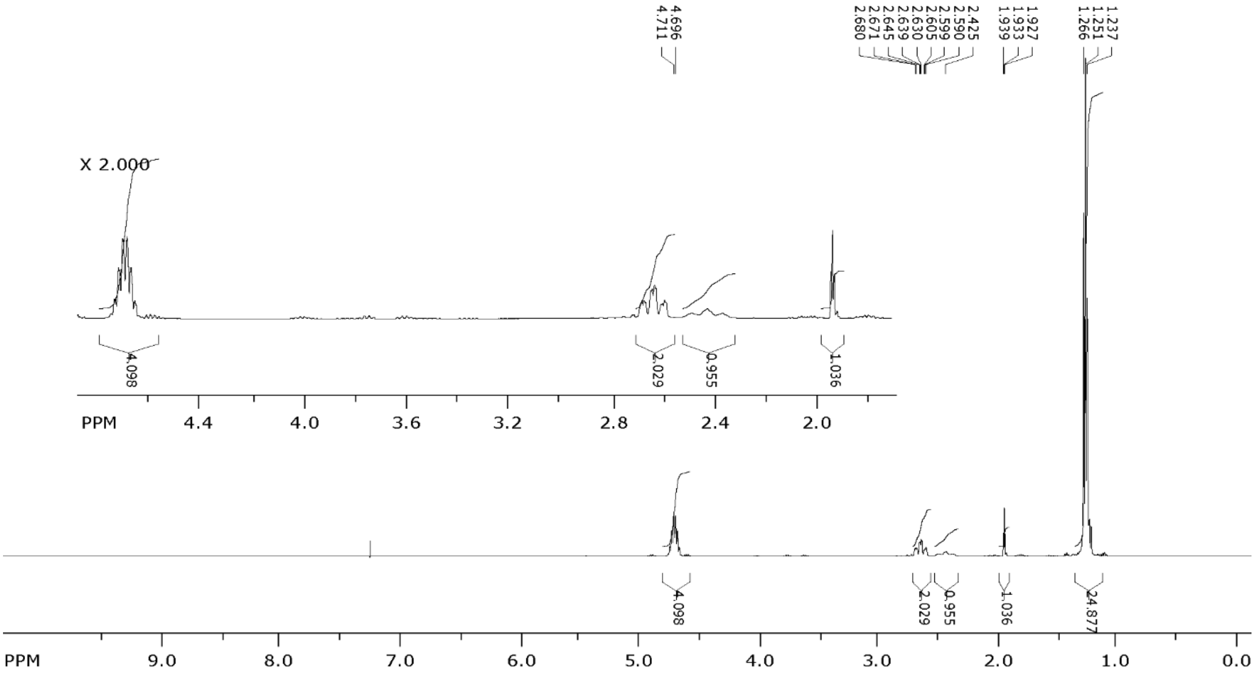

2.2. *2-(4-((4-(2,2-Bis(diisopropoxyphosphoryl)ethyl)-1H-1,2,3-triazol-1-yl)methyl)phenyl)-2,5,5-triethyl-4-pyrrolidino-2,5-dihydro-1H-imidazol-1-oxyl (**7**)(300 MHz; $CD_3OD–CDCl_3$, reduced with Zn in ND_4Cl/D_2O)*

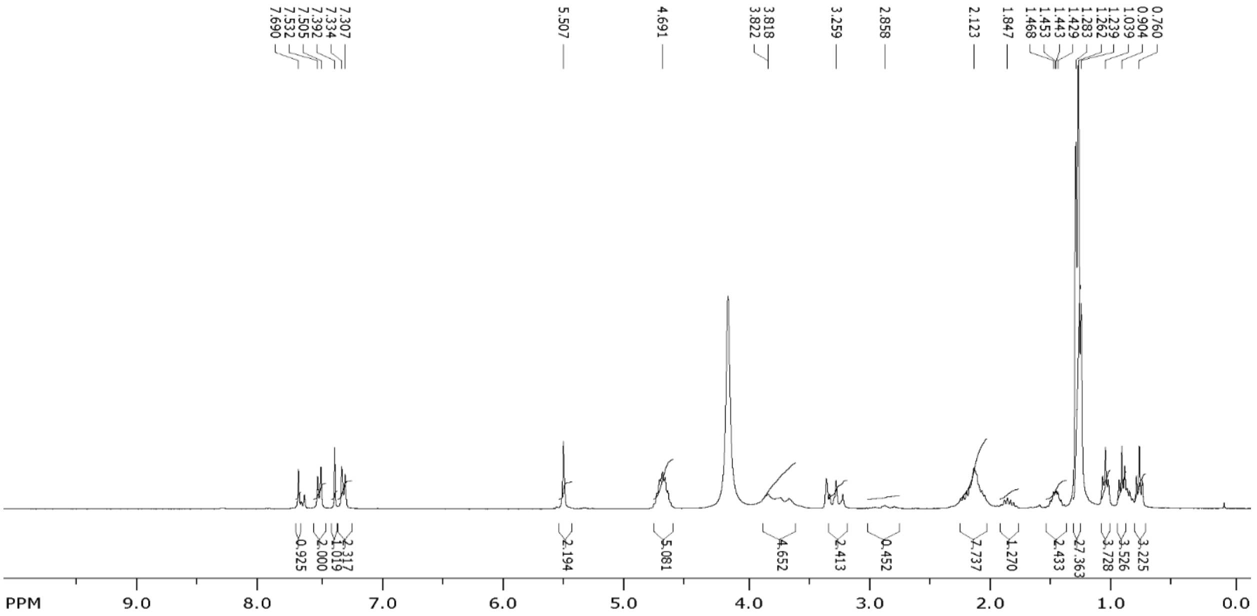

2.3. *2-(4-Carboxyphenyl)-2,5,5-triethyl-4-pyrrolidino-2,5-dihydro-1H-imidazol-1-oxyl (**12**) (400 MHz; $CD_3OD–CDCl_3$, reduced with PhSH)*

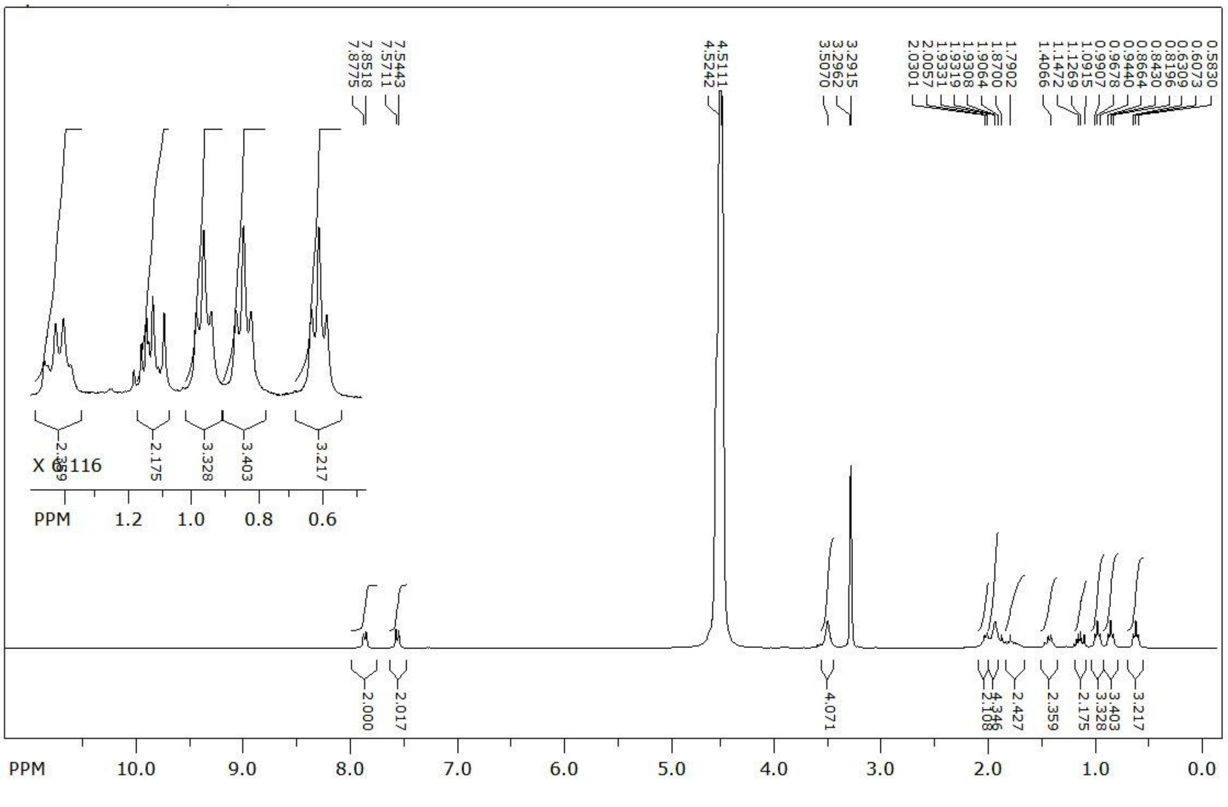

2.4. *2-(4-(Ethoxycarbonyl)phenyl)-2,5,5-triethyl-4-pyrrolidino-2,5-dihydro-1H-imidazol-1-oxyl* (**14**) *(300 MHz; $CDCl_3$–CD_3OD, reduced with Zn/CF_3COOH in CD_3OD, 65 °C)*

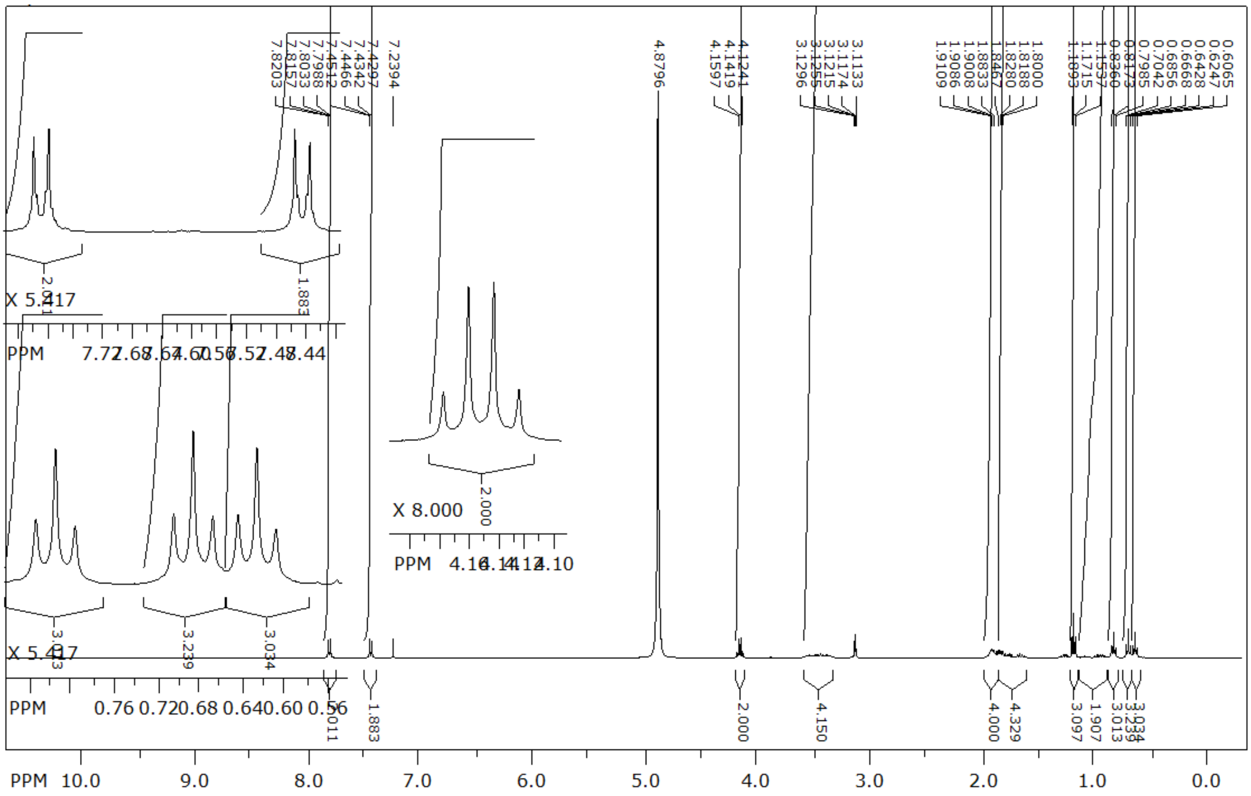

2.5. *2-(4-((3-Carboxypropanoyloxy)methyl)phenyl)-2,5,5-triethyl-4-pyrrolidino-2,5-dihydro-1H-imidazol-1-oxyl* (**16**) *(300 MHz; $CDCl_3$ – CD_3OD, reduced with Zn/CF_3COOH in CD_3OD, 65 °C)*

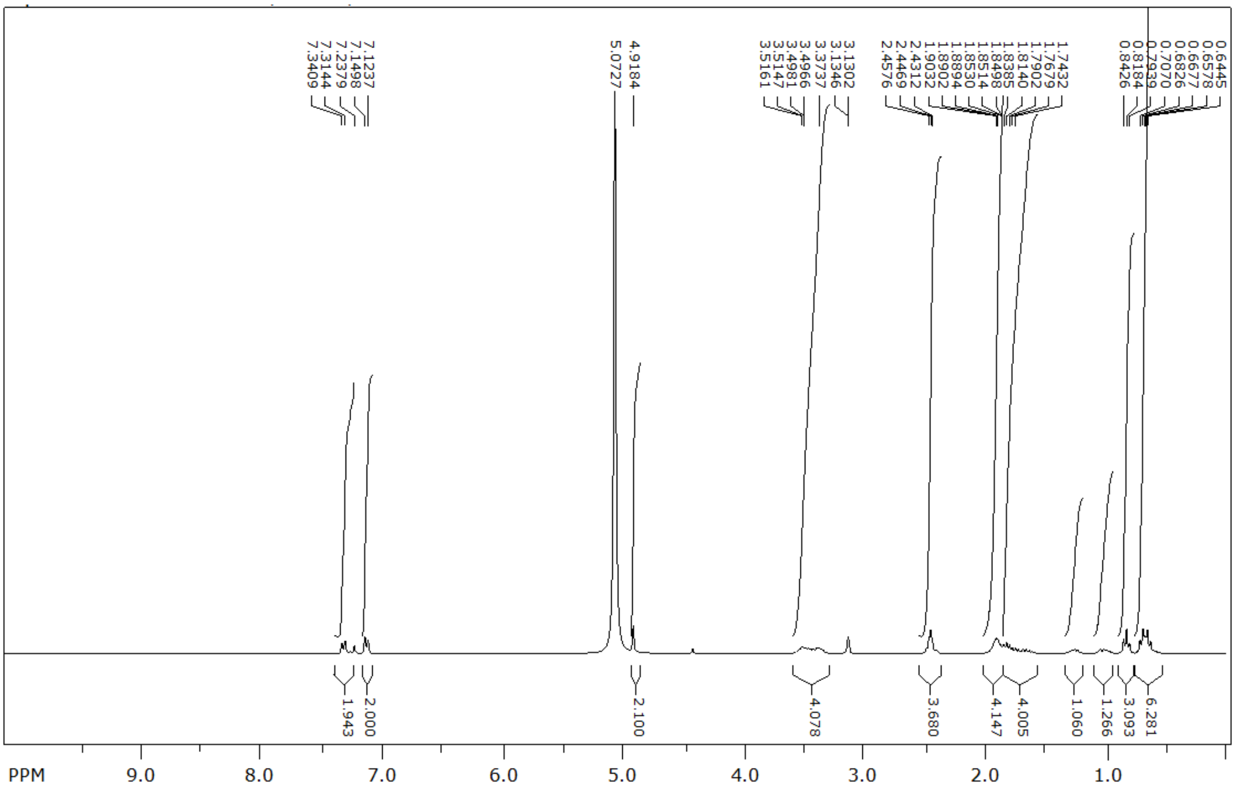

*2.6. 2-Allyl-2-ethyl-5,5-dimethyl-4-(pyrrolidino)-2,5-dihydroimidazol-1-oxyl (**18a**) (400 MHz; CD_3OD, reduced with N_2D_4)*

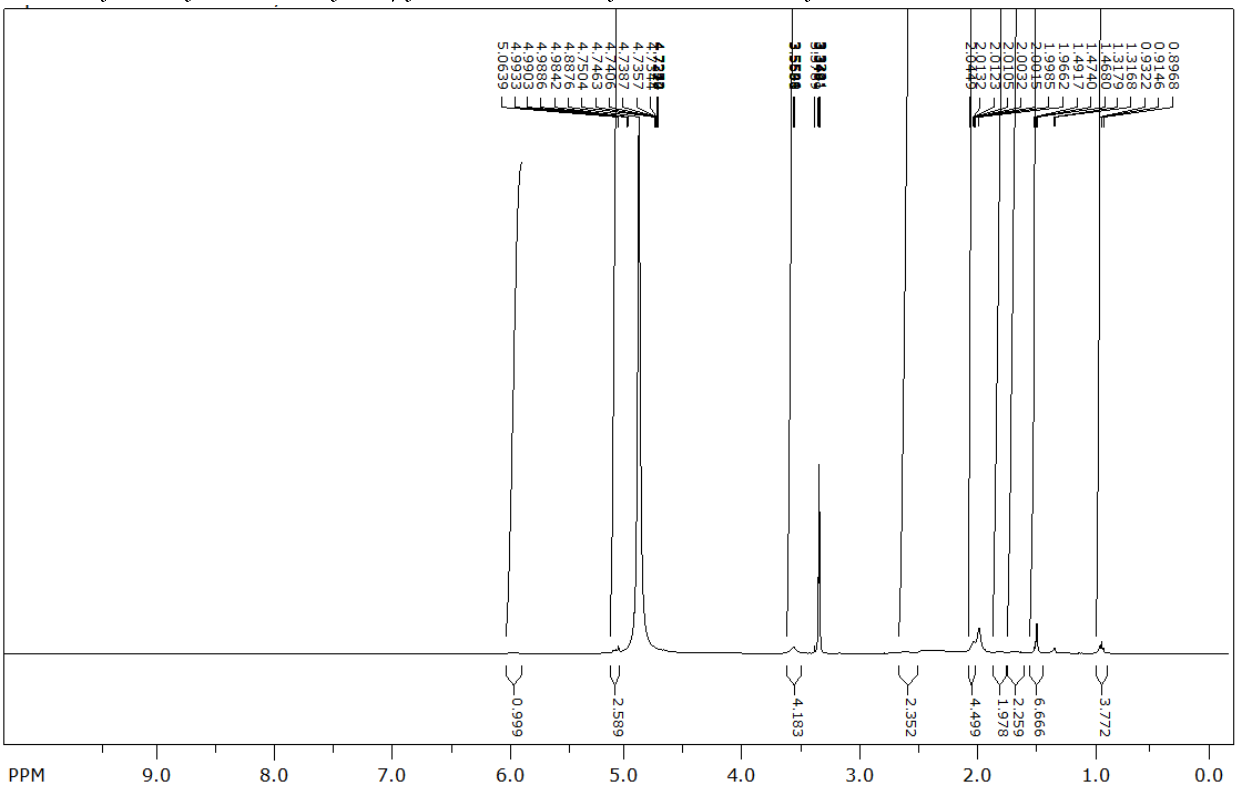

*2.7. 2-Ethyl-5,5-dimethyl-2-(pent-4-enyl)-4-(pyrrolidino)-2,5-dihydroimidazol-1-oxyl (**18b**) (300 MHz; $CDCl_3$ – CD_3OD, reduced with Zn/CF_3COOH in CD_3OD, 65 °C)*

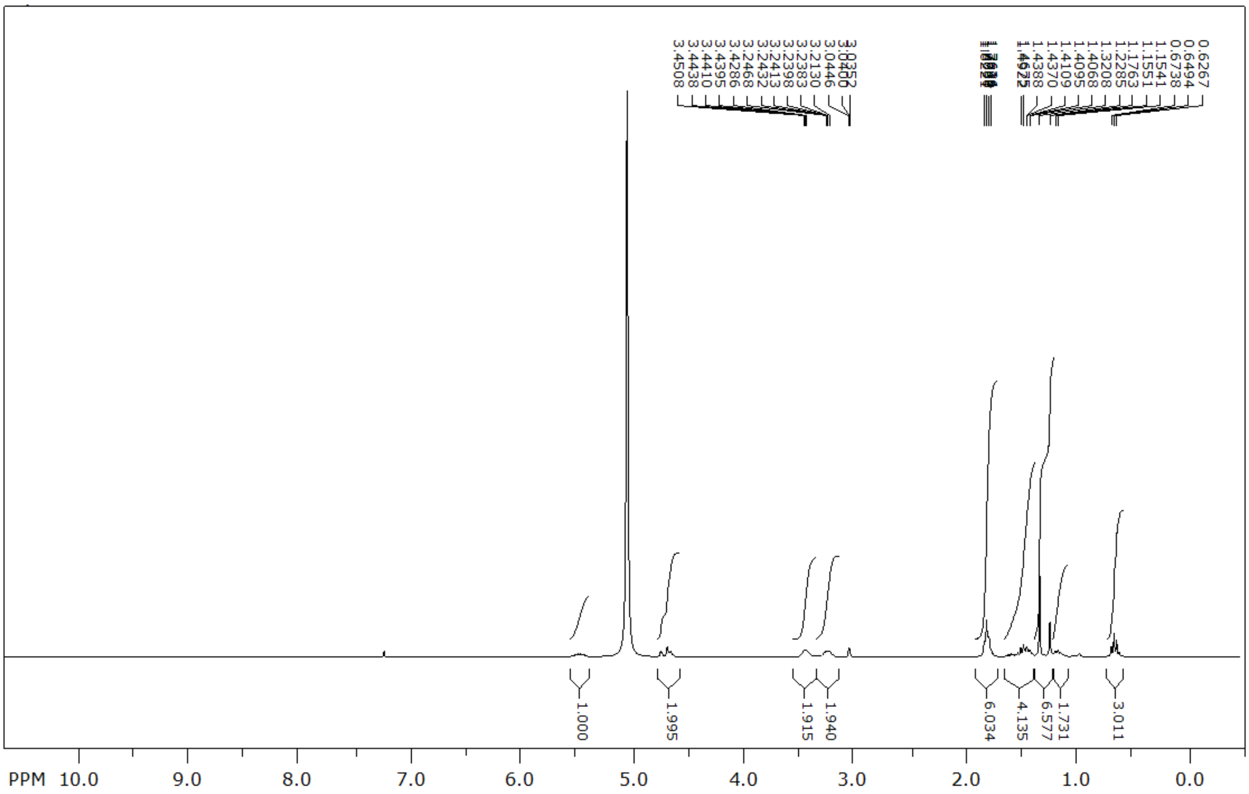

*2.8. 2-Ethyl-2-(3-hydroxypropyl)-5,5-dimethyl-4-(pyrrolidino)-2,5-dihydroimidazol-1-oxyl (**19a**) (400 MHz; CD_3OD, reduced with N_2D_4)*

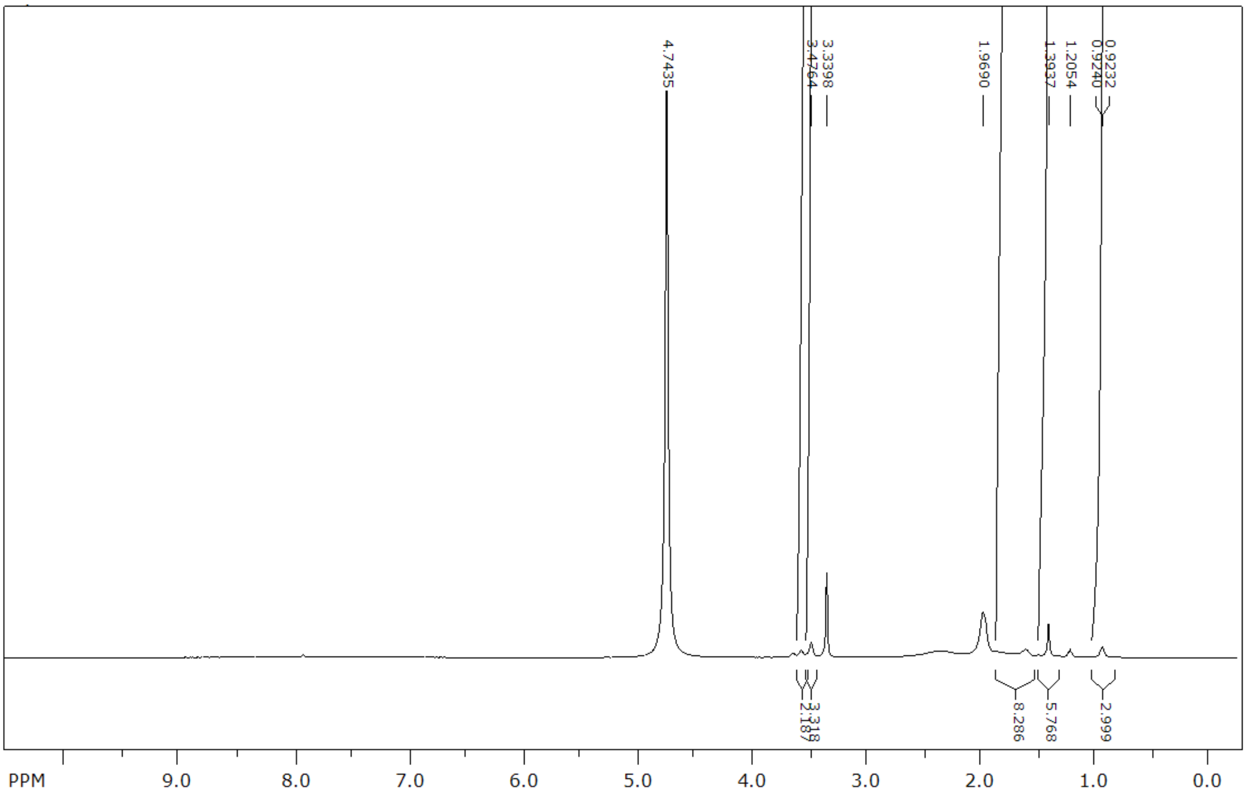

*2.9. 2-Ethyl-2-(5-hydroxypentyl)-5,5-dimethyl-4-(pyrrolidino)-2,5-dihydroimidazol-1-oxyl (**19b**) (300 MHz; $CDCl_3$–CD_3OD, reduced with Zn/CF_3COOH in CD_3OD, 65 °C)*

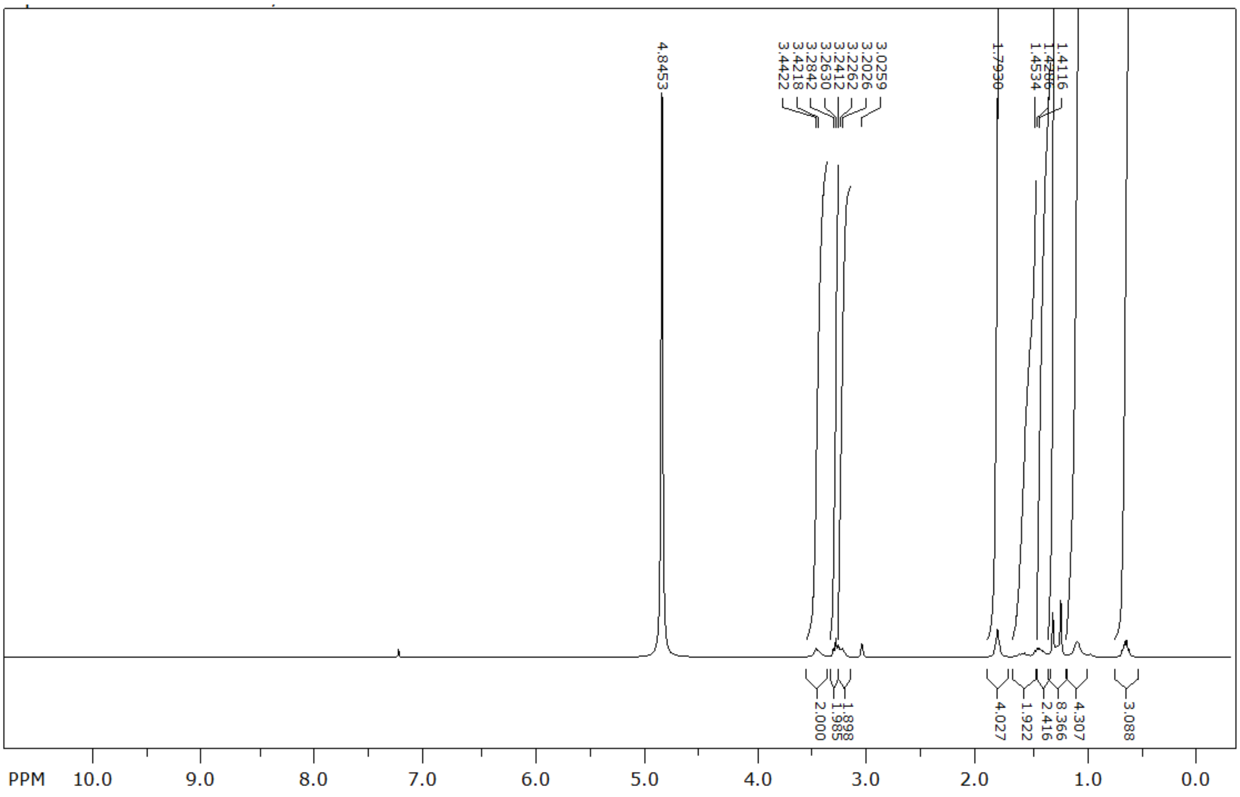

2.10. *2-(3-(3-(Diethylamino)propylcarbamoyloxy)propyl)-2-ethyl-5,5-dimethyl-4-(pyrrolidino)-2,5-dihydro-1H-imidazol-1-oxyl (**21**) (300 MHz; $CDCl_3$ – CD_3OD, reduced with Zn/CF_3COOH in CD_3OD, 65 °C)*

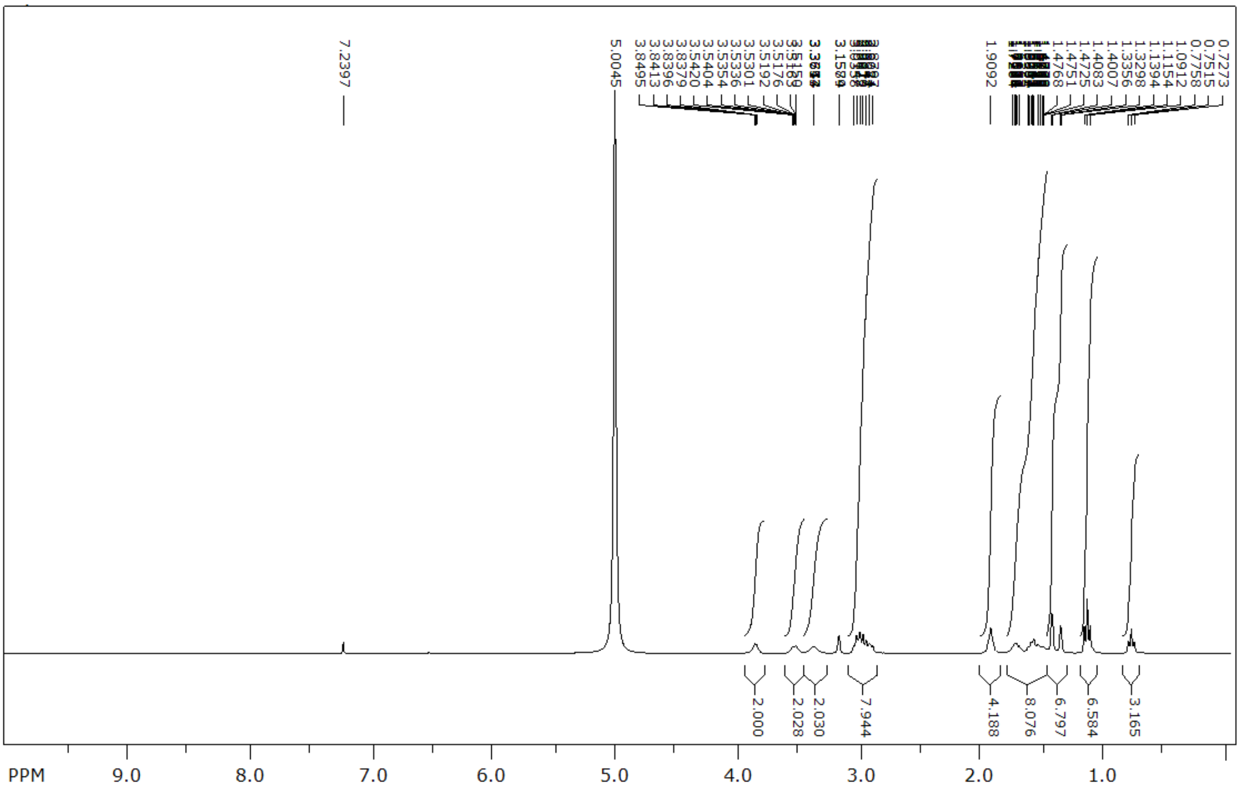

2.11. *2-(2-carboxyethyl)-2-ethyl-5,5-dimethyl-4-(pyrrolidin-1-yl)-2,5-dihydro-1H-imidazol-1-oxyl (**25**) (300 MHz; $CDCl_3$–CD_3OD, reduced with Zn/CF_3COOH in CD_3OD, 65 °C)*

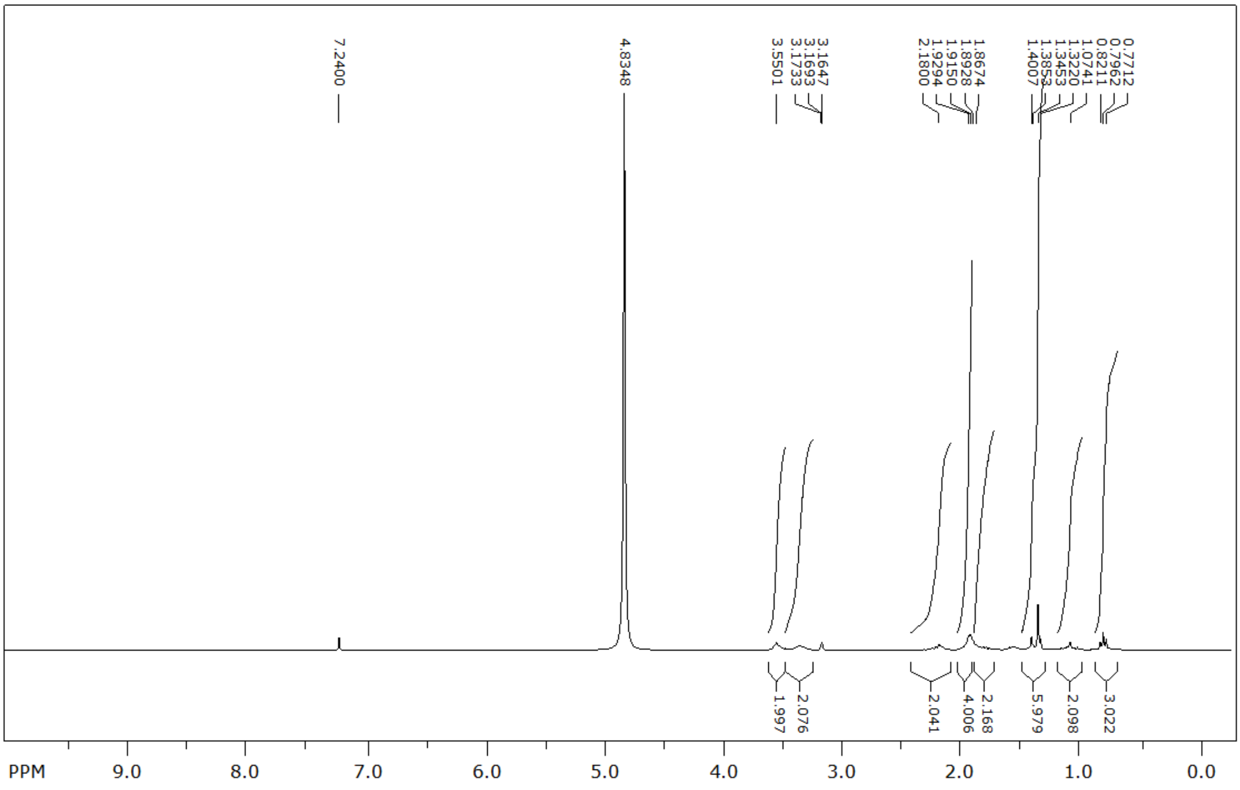

*2.12. 1-(4-(1,3-dioxolan-2-yl)phenyl)-N-methylmethanamine (**26**) (300 MHz; $CDCl_3$)*

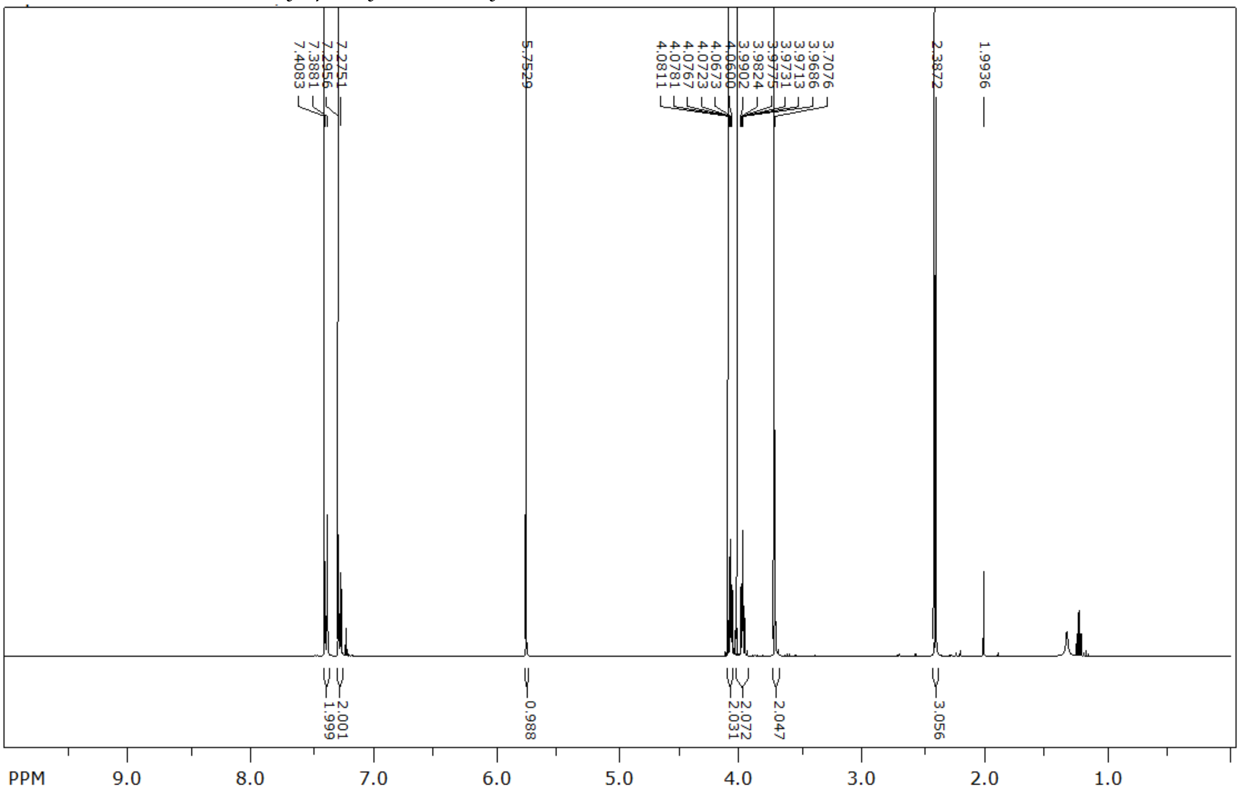

*2.13. 5-((4-(1,3-dioxolan-2-yl)benzyl)(methyl)amino)-4,4-dimethyl-2-(pyridin-4-yl)-4H-imidazole 3-oxide (**30**) (400 MHz; $CDCl_3$)*

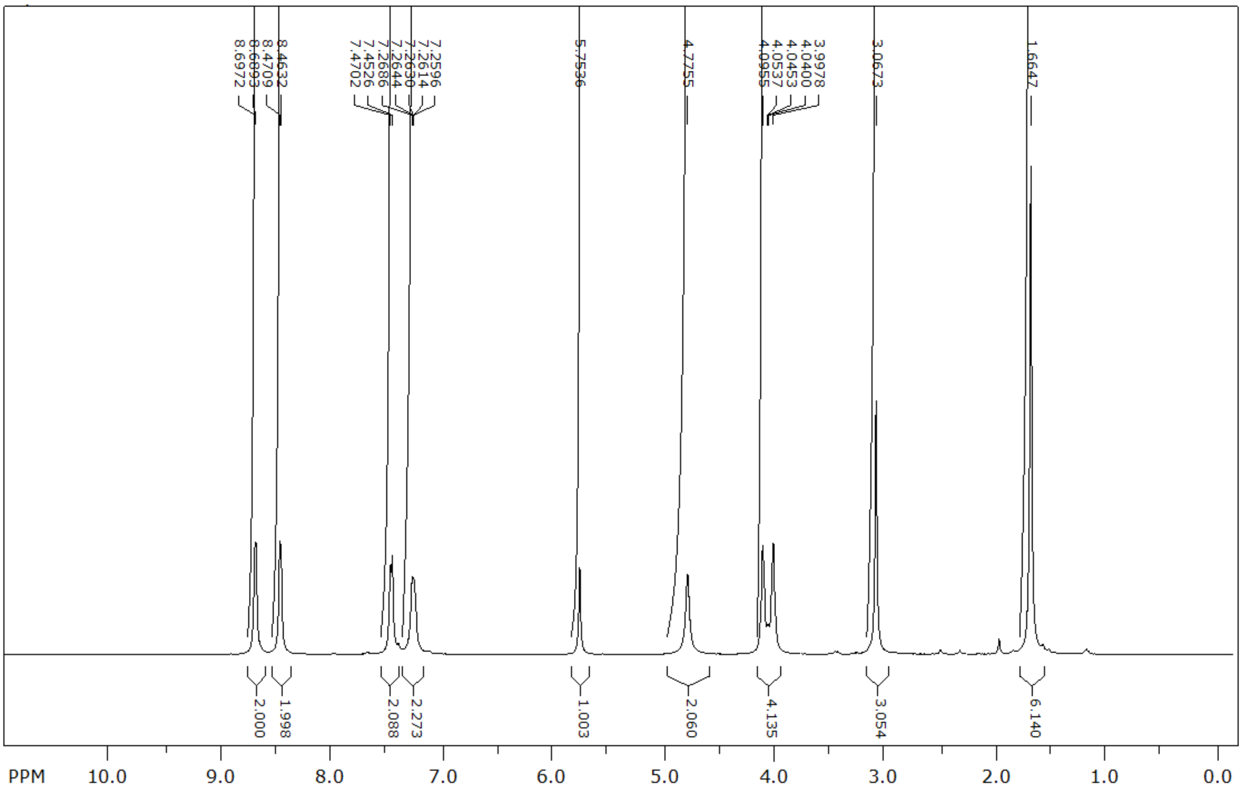

2.14. *2-Ethyl-4-((4-(hydroxymethyl)benzyl)(methyl)amino)-5,5-dimethyl-2-(pyridin-4-yl)-2,5-dihydroimidazol-1-oxyl (**34**) (400 MHz; $CDCl_3$, CD_3OD, reduced with Zn/CF_3COOH in CD_3OD)*

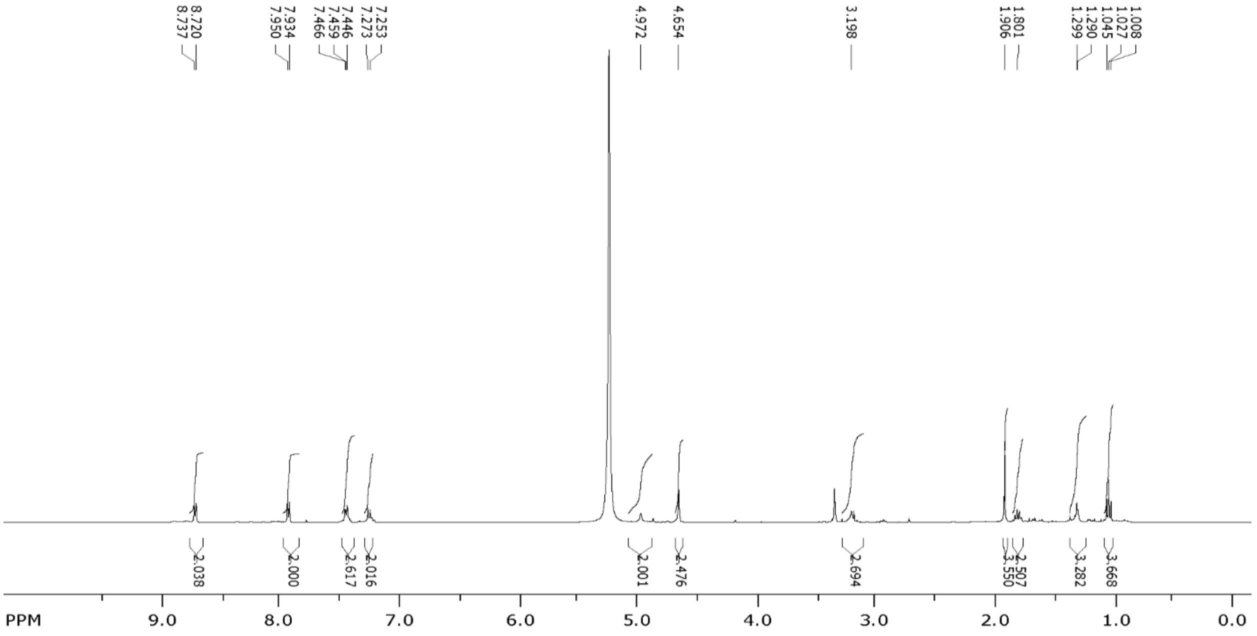

3. ^{13}C NMR Spectra

3.1. *Tetraisopropyl but-3-yne-1,1-diyldiphosphonate(**6**)(75 MHz, $CDCl_3$)*

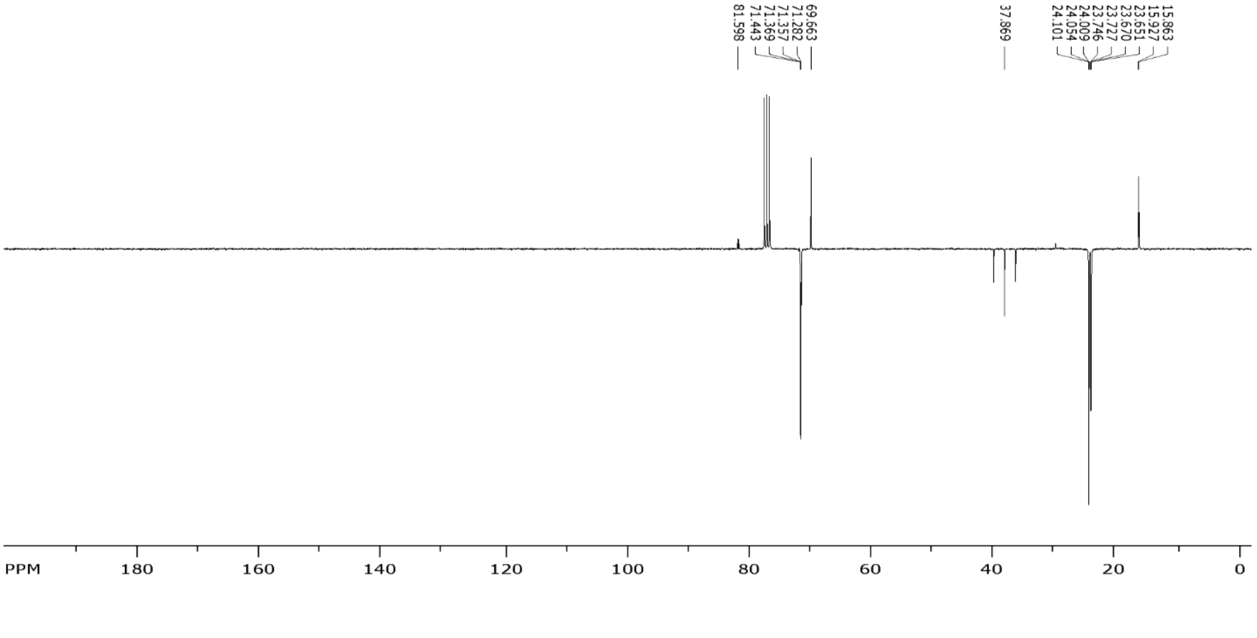

*3.2. 1-(4-(1,3-dioxolan-2-yl)phenyl)-N-methylmethanamine (**26**) (75 MHz; CDCl3)*

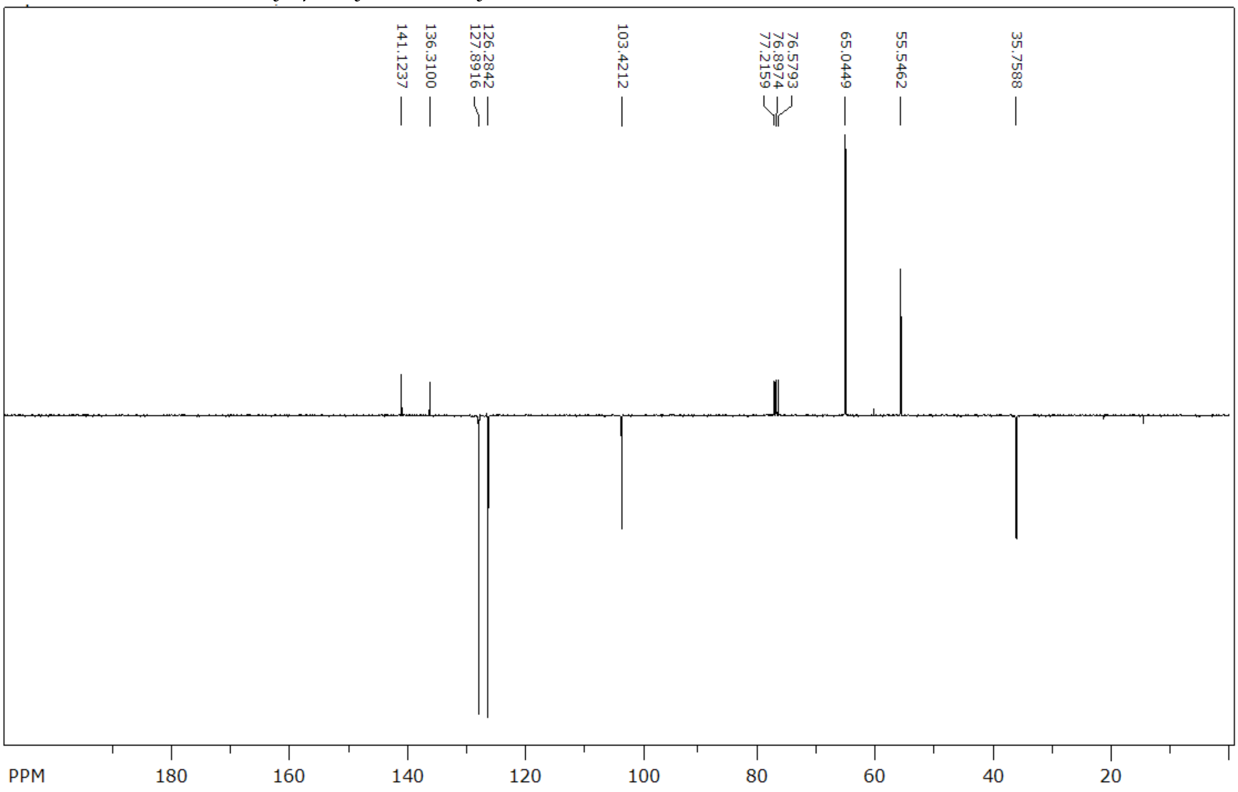

*3.3. 5-((4-(1,3-dioxolan-2-yl)benzyl)(methyl)amino)-4,4-dimethyl-2-(pyridin-4-yl)-4H-imidazole 3-oxide (**30**) (75 MHz; CDCl3)*

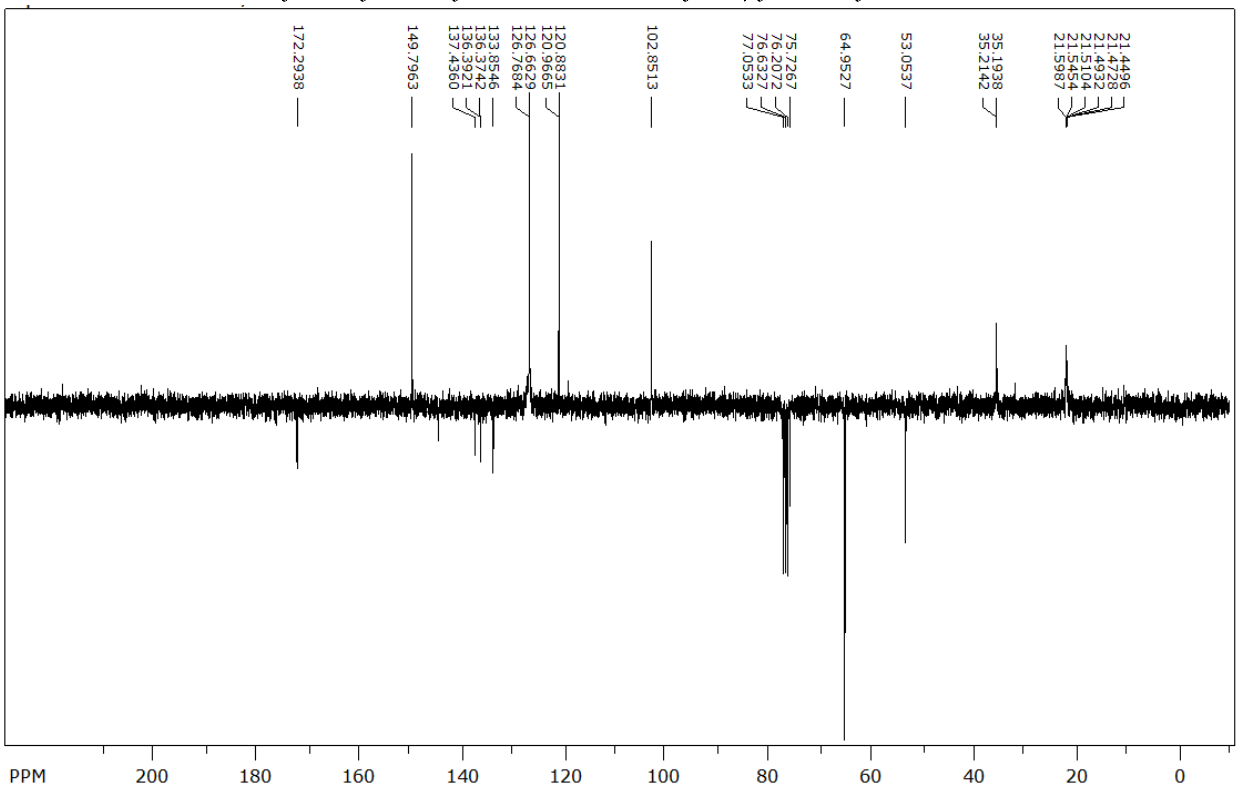

4. Titration Data.

*4.1. 2-(4-(Azidomethyl)phenyl)-2,5,5-triethyl-4-pyrrolidino-2,5-dihydro-1H-imidazol-1-oxyl (**5**)*

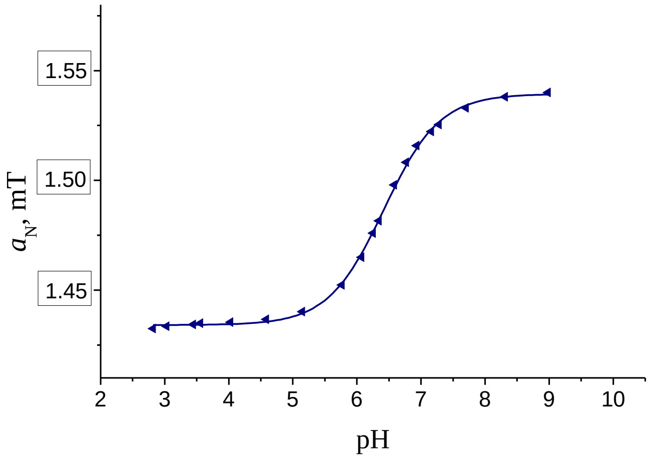

*4.2. 2-(4-((4-(2,2-Bis(diisopropoxyphosphoryl)ethyl)-1H-1,2,3-triazol-1-yl)methyl)phenyl)-2,5,5-triethyl-4-pyrrolidino-2,5-dihydro-1H-imidazol-1-oxyl (**7**)*

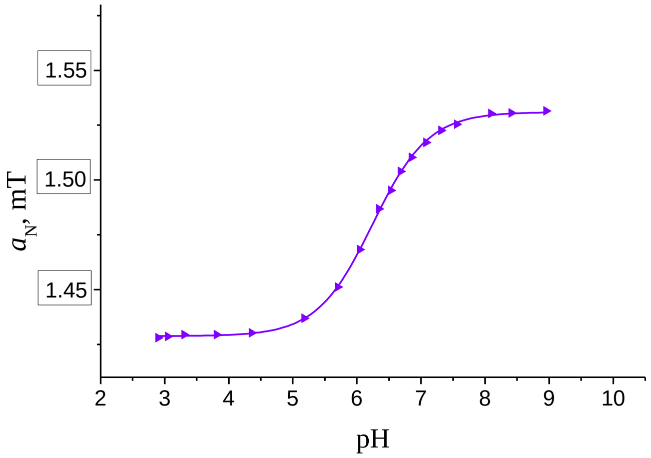

*4.3. 2,5,5-Triethyl-2-(4-ethynylphenyl)-4-pyrrolidino-2,5-dihydro-1H-imidazol-1-oxyl (**11**)*

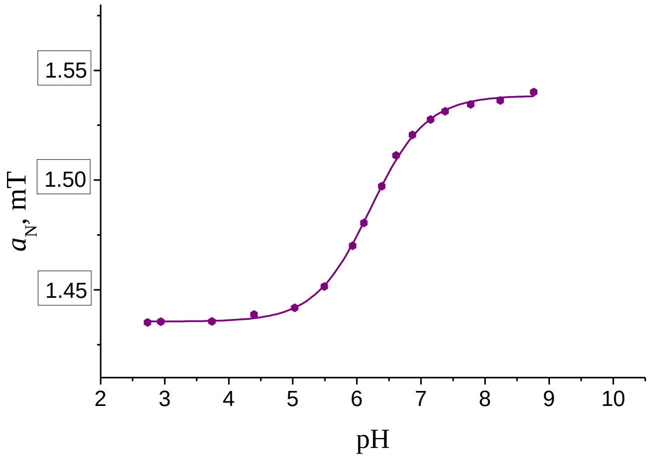

*4.4 2-(4-Carboxyphenyl)-2,5,5-triethyl-4-pyrrolidino-2,5-dihydro-1H-imidazol-1-oxyl (**12**)*

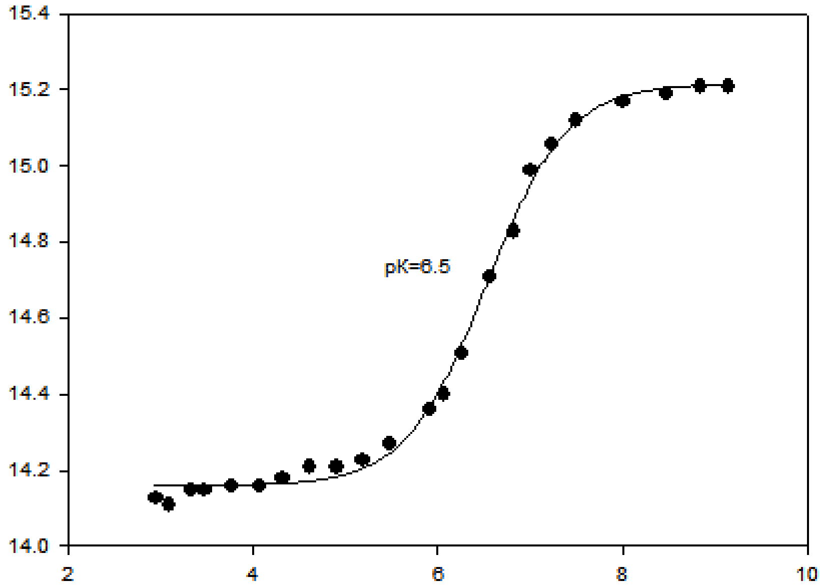

*4.5. 2-(4-((2,5-Dioxopyrrolidinooxy)carbonyl)phenyl)-2,5,5-triethyl-4-pyrrolidino-2,5-dihydro-1H-imidazol-1-oxyl (**15**)*

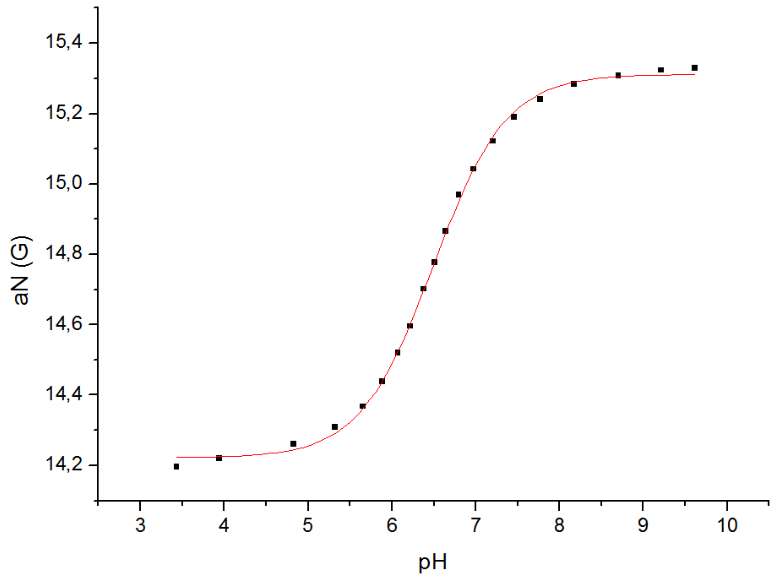

*4.6. 2-(4-((3-Carboxypropanoyloxy)methyl)phenyl)-2,5,5-triethyl-4-pyrrolidino-2,5-dihydro-1H-imidazol-1-oxyl (**16**)*

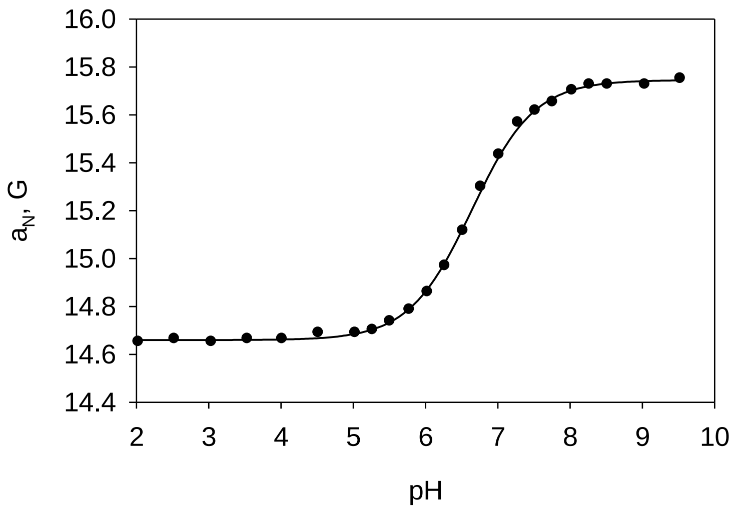

*4.7. 2-Allyl-2-ethyl-5,5-dimethyl-4-(pyrrolidino)-2,5-dihydroimidazol-1-oxyl (**18a**)*

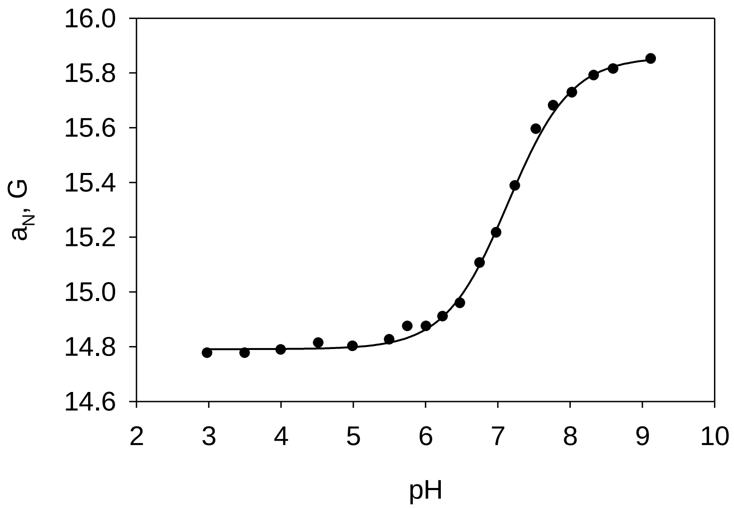

*4.8. 2-Ethyl-5,5-dimethyl-2-(pent-4-enyl)-4-(pyrrolidino)-2,5-dihydroimidazol-1-oxyl (**18b**)*

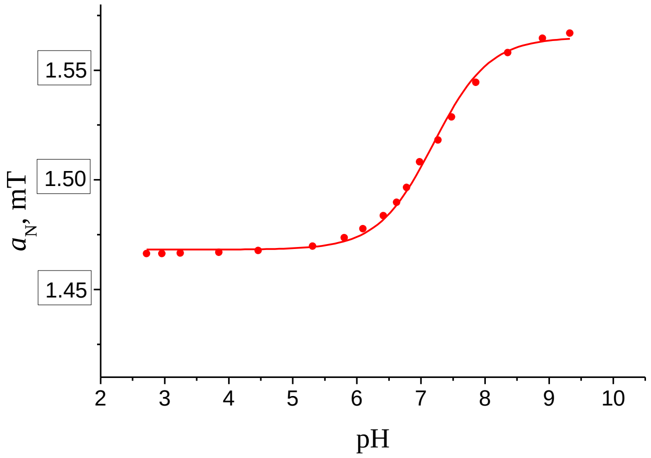

*4.9. 2-Ethyl-2-(3-hydroxypropyl)-5,5-dimethyl-4-(pyrrolidino)-2,5-dihydroimidazol-1-oxyl (**19a**)*

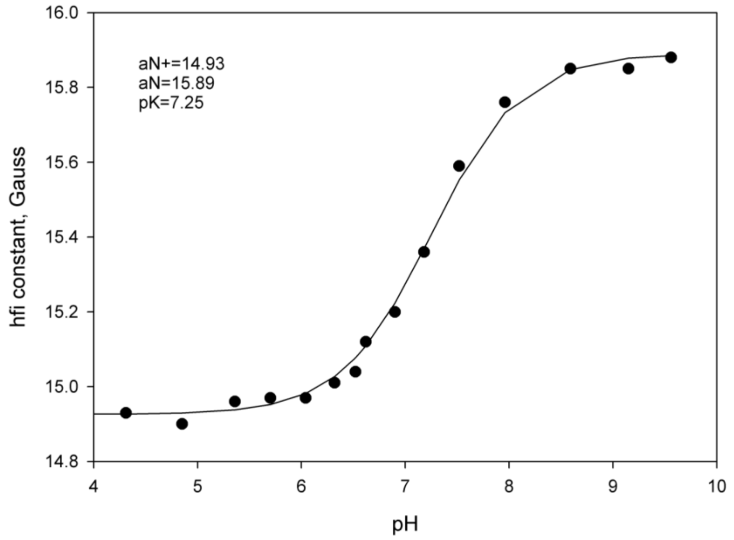

*4.10. 2-Ethyl-2-(5-hydroxypentyl)-5,5-dimethyl-4-(pyrrolidino)-2,5-dihydroimidazol-1-oxyl (**19b**)*

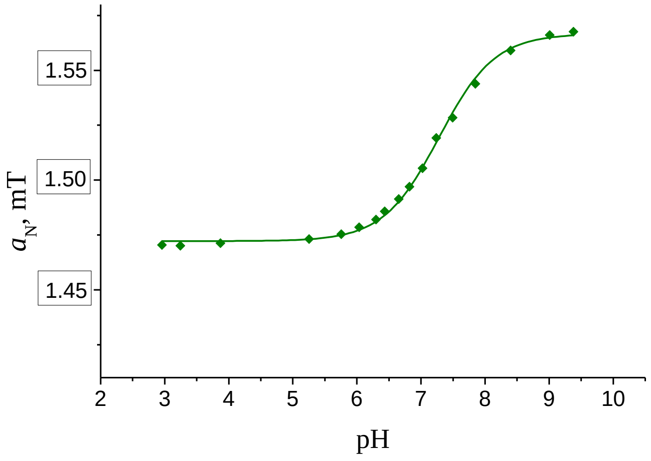

*4.11. 2-(3-(1H-Imidazole-1-carbonyloxy)propyl)-2-ethyl-5,5-dimethyl-4-(pyrrolidino)-2,5-dihydro-1H-imidazol-1-oxyl (**20a**)*

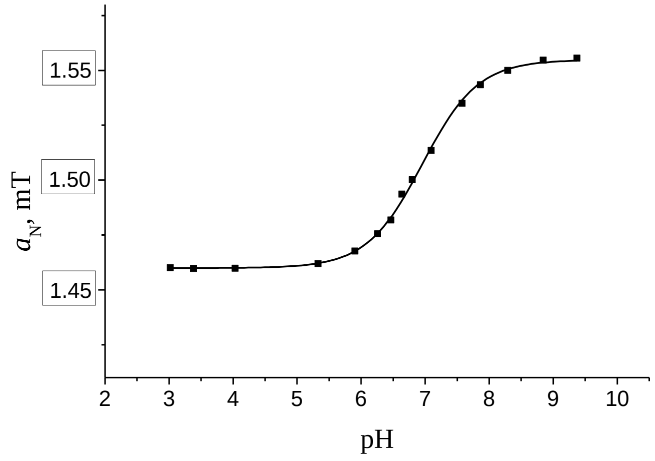

*4.12. 2-(3-(3-(Diethylamino)propylcarbamoyloxy)propyl)-2-ethyl-5,5-dimethyl-4-(pyrrolidino)-2,5-dihydro-1H-imidazol-1-oxyl (**21**)*

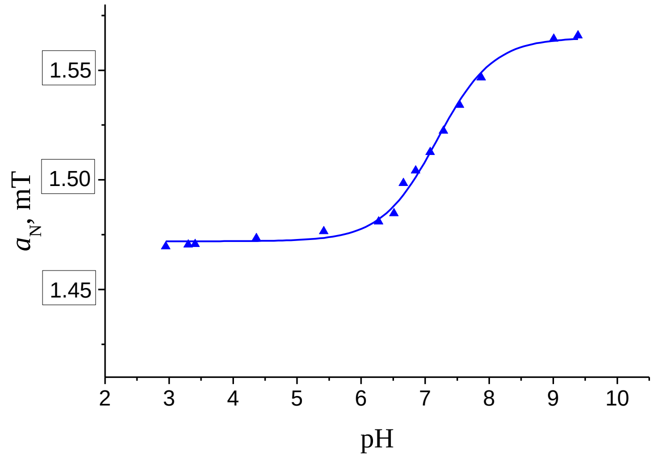

*4.13. 2-(3-Carboxypropyl)-2-ethyl-5,5-dimethyl-4-(pyrrolidino)-2,5-dihydro-1H-imidazol-1-oxyl (**22**)*

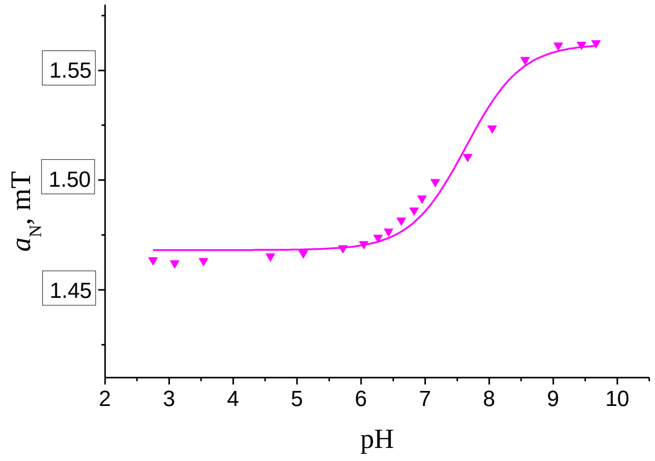

4.14. 2-(2-(1,3-dioxolan-2-yl)ethyl)-2-ethyl-5,5-dimethyl-4-(pyrrolidin-1-yl)-2,5-dihydro-1H-imidazol-1-oxyl (23)

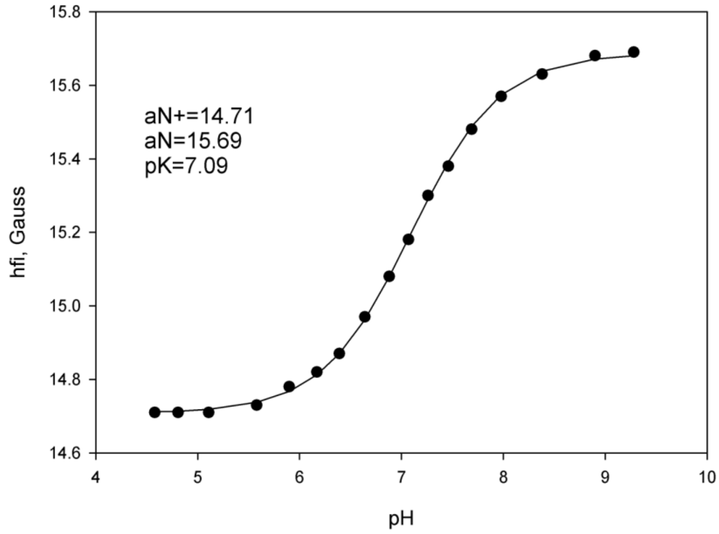

*4.15. 2-(2-carboxyethyl)-2-ethyl-5,5-dimethyl-4-(pyrrolidin-1-yl)-2,5-dihydro-1H-imidazol-1-oxyl (***25***)*

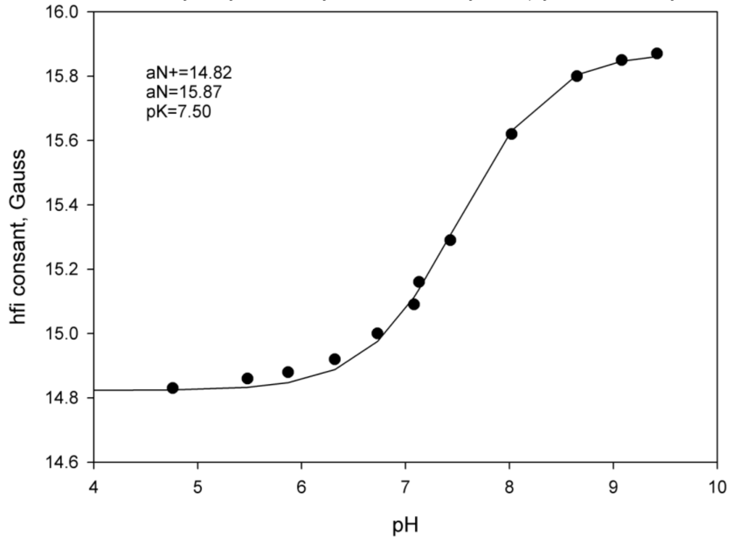

4.16. 2-ethyl-4-((4-formylbenzyl)(methyl)amino)-5,5-dimethyl-2-(pyridin-4-yl)-2,5-dihydro-1H-imidazol-1-oxyl (32)

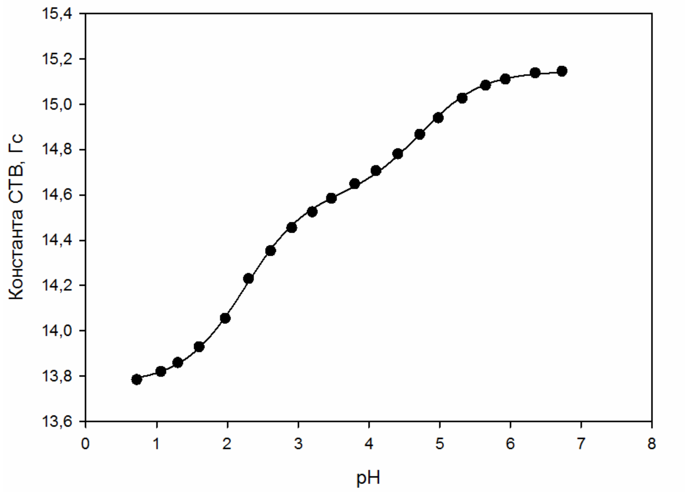

*4.17. 4-((4-carboxybenzyl)(methyl)amino)-2-ethyl-5,5-dimethyl-2-(pyridin-4-yl)-2,5-dihydro-1H-imidazol-1-oxyl (***33***)*

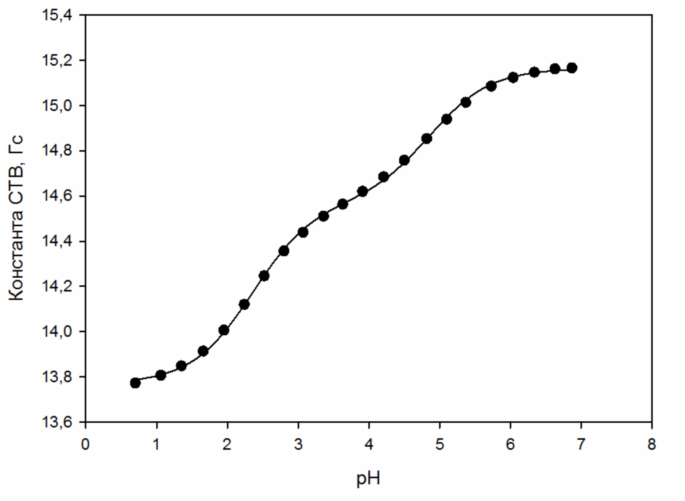

4.18. *2-ethyl-4-((4-(hydroxymethyl)benzyl)(methyl)amino)-5,5-dimethyl-2-(pyridin-4-yl)-2,5-dihydro-1H-imidazol-1-oxyl* (**34**)

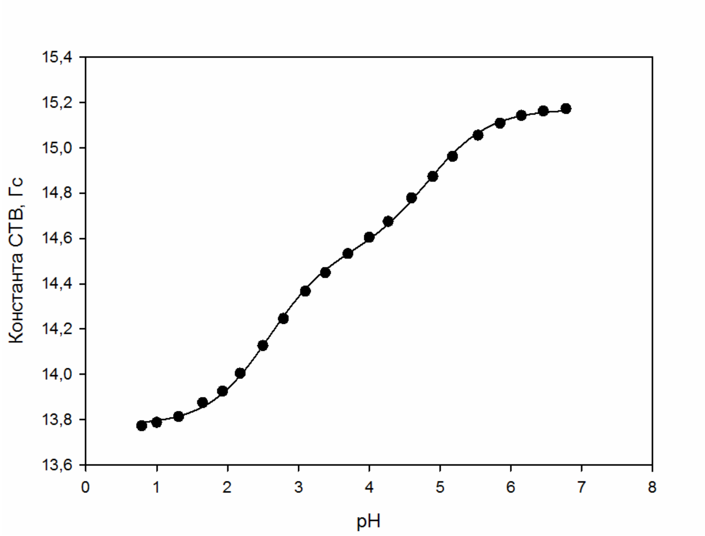

4.19. *4-((4-(((2,5-dioxopyrrolidin-1-yl)oxy)carbonyl)benzyl)(methyl)amino)-2-ethyl-5,5-dimethyl-2-(pyridin-4-yl)-2,5-dihydro-1H-imidazol-1-oxyl* (**35**)

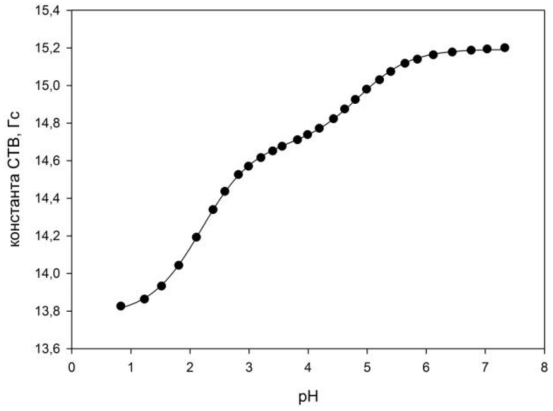

5. X-Ray Data for Nitroxide 11.

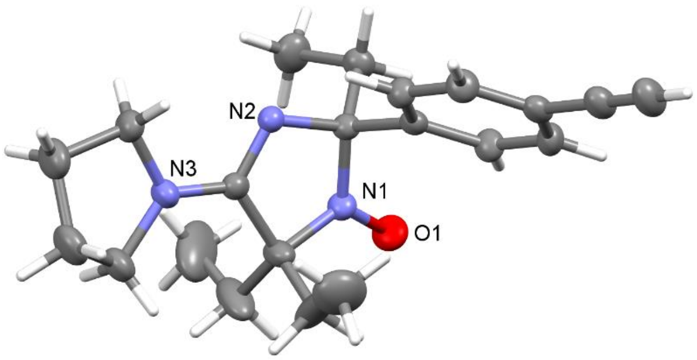

Figure S1. The molecular structure of 2,5,5-triethyl-2-(4-ethynylphenyl)-4-pyrrolidino-2,5-dihydro-1H-imidazol-1-oxyl (**11**) (minor position of disordered geminal ethyl group is omitted).

Crystallographic data for **11**: $C_{21}H_{28}N_3O$, *M* 338.46, monoclinic, $P2_1/c$, *a* 8.7388(3), *b* 15.2737(6), *c*14.5589(6) Å, β 100.656(2)º*V* 1909.7(1) Å^3, *Z* 4, D_{calcd} 1.177,g·cm^{-3}, μ(Mo-$K\alpha$) 0.073 mm^{-1}, F(000) 732, (θ 2.67 – 25.36°, completeness 99.9%), T 200(2) K, orange needle, (1.00 × 0.32 × 0.14) mm^3, transmission 0.782 − 0.862, 18099 measured reflections in index range -10<=h<=10, -18<=k<=18, -17<=l<=17, 3513 independent (R_{int} 0.0351), 243 parameters, R_1 0.0434 (for 2820 observed $I>2\sigma(I)$), wR_2 0.1154 (all data), GOOF 1.025, largest diff. peak and hole 0.27 and -0.28 e.A^{-3}.
